# *Trans*-vaccenic acid reprograms CD8^+^ T cells and anti-tumour immunity

**DOI:** 10.1038/s41586-023-06749-3

**Published:** 2023-11-22

**Authors:** Hao Fan, Siyuan Xia, Junhong Xiang, Yuancheng Li, Matthew O. Ross, Seon Ah Lim, Fan Yang, Jiayi Tu, Lishi Xie, Urszula Dougherty, Freya Q. Zhang, Zhong Zheng, Rukang Zhang, Rong Wu, Lei Dong, Rui Su, Xiufen Chen, Thomas Althaus, Peter A. Riedell, Patrick B. Jonker, Alexander Muir, Gregory B. Lesinski, Sarwish Rafiq, Madhav V. Dhodapkar, Wendy Stock, Olatoyosi Odenike, Anand A. Patel, Joseph Opferman, Takemasa Tsuji, Junko Matsuzaki, Hardik Shah, Brandon Faubert, Shannon E. Elf, Brian Layden, B. Marc Bissonnette, Yu-Ying He, Justin Kline, Hui Mao, Kunle Odunsi, Xue Gao, Hongbo Chi, Chuan He, Jing Chen

**Affiliations:** 1 Department of Hematology and Medical Oncology, Emory University, Atlanta, GA USA; 2 Winship Cancer Institute, Emory University, Atlanta, GA USA; 3 Department of Medicine, The University of Chicago, Chicago, IL USA; 4 Department of Chemistry, The University of Chicago, Chicago, IL USA; 5 Department of Radiology and Imaging Sciences, Emory University, Atlanta, GA USA; 6 Department of Immunology, St Jude Children’s Research Hospital, Memphis, TN USA; 7 Department of Systems Biology, Beckman Research Institute of City of Hope, Duarte, CA USA; 8 The Ben May Department for Cancer Research, The University of Chicago, Chicago, IL USA; 9 Department of Obstetrics and Gynecology, The University of Chicago, Chicago, IL USA; 10 Department of Medicine, University of Illinois Chicago, Chicago, IL USA; 11 Present Address: Department of Human Cell Biology and Genetics, Southern University of Science and Technology School of Medicine, Shenzhen, China; 12 Present Address: Department of Surgery, University of Michigan, Ann Arbor, MI USA

**Keywords:** Cancer metabolism, Immunosurveillance

## Abstract

Diet-derived nutrients are inextricably linked to human physiology by providing energy and biosynthetic building blocks and by functioning as regulatory molecules. However, the mechanisms by which circulating nutrients in the human body influence specific physiological processes remain largely unknown. Here we use a blood nutrient compound library-based screening approach to demonstrate that dietary *trans*-vaccenic acid (TVA) directly promotes effector CD8^+^ T cell function and anti-tumour immunity in vivo. TVA is the predominant form of *trans*-fatty acids enriched in human milk, but the human body cannot produce TVA endogenously^1^. Circulating TVA in humans is mainly from ruminant-derived foods including beef, lamb and dairy products such as milk and butter^2,3^, but only around 19% or 12% of dietary TVA is converted to rumenic acid by humans or mice, respectively^4,5^. Mechanistically, TVA inactivates the cell-surface receptor GPR43, an immunomodulatory G protein-coupled receptor activated by its short-chain fatty acid ligands^6–8^. TVA thus antagonizes the short-chain fatty acid agonists of GPR43, leading to activation of the cAMP–PKA–CREB axis for enhanced CD8^+^ T cell function. These findings reveal that diet-derived TVA represents a mechanism for host-extrinsic reprogramming of CD8^+^ T cells as opposed to the intrahost gut microbiota-derived short-chain fatty acids. TVA thus has translational potential for the treatment of tumours.

## Abstract (web summary)

A screen of nutrient-derived compounds identified *trans*-vaccenic acid as a promoter of effector T cell function, and functional assays demonstrate that this occurs via inactivation of GPR43 on T cells.

## Main

Diet-related nutritional availability represents a crucial element in the environmental influences that have shaped human physiology during millions of years of human evolution, which has witnessed substantial dietary changes that are associated with physiological and pathological adaptions in human^9^. However, despite extensive studies on links between diet and human health and disease, little is known about how the circulating diet-derived nutrients affect specific human physiological and pathological processes. The main reason is that mechanistic studies to decipher such links are difficult owing to the vast diversity of foods and the high complexity of diet metabolism at organismal levels. We thus approached this dilemma by assembling a blood nutrient compound library and performed screens to initially identify dietary nutrients that influence anti-tumour immunity. Here, we report that dietary TVA (also known as (11*E*)-octadec-11-enoic acid), promotes tumour-infiltrating and cytotoxic functions of effector CD8^+^ T cells, leading to enhanced anti-tumour immunity in vivo.

Our integrated temporal genomics and protein phosphorylation analyses reveal that the cell-surface receptor GPR43 is a target of TVA. GPR43 (also known as free fatty acid receptor 2 (FFAR2)) is a short-chain fatty acid (SCFA)-binding G-protein-coupled receptor (GPCR) that is activated by its agonists—including acetate, propionate and butyrate—which are SCFAs produced during dietary fibre fermentation by resident bacteria in gut microbiota^10^. GPR43 couples with both Gα_i_ and Gα_q_ (ref. ^11^). GPR43 is suggested to be involved in anti-inflammation by mediating functions of its SCFA agonists including propionate^6,7^. Although it has been reported that SCFAs require GPR41 and GPR43 to establish memory CD8^+^ T cells with optimized recall responses^8^, GPR41 and GPR43 have also been shown to be dispensable for SCFAs to promote CD8^+^ T cell anti-tumour activity^12,13^. However, whether GPR43 functions as a sensor of long-chain fatty acids in the regulation of effector CD8^+^ T cell function remains unknown. Here, we show that TVA—a long-chain fatty acid—attenuates Gα_i_-coupling GPR43 activity and leads to increased cAMP levels, which antagonizes the overall negative effects of SCFAs on cAMP to enhance effector CD8^+^ T cell function.

## Nutrients that enhance T cell function

**Fig. 1:**
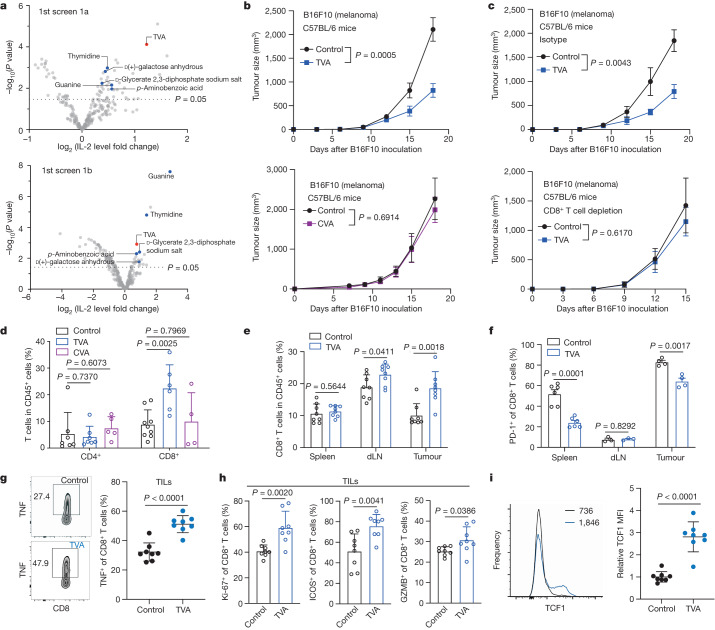
Dietary TVA enhances anti-tumour immunity through effector CD8^+^ T cells. **a**, Scatter plot showing a summary of the initial screens (*n* = 4) to identify nutrients that enhance Jurkat T cell activation (1a, top) or reverse PD-L1–PD-1 mediated PD-1^+^ Jurkat T cell exhaustion induced by co-cultured H596 (PD-L1^+^) human lung cancer cells (1b, bottom). Fold change of IL-2 was obtained by comparing IL-2 production in the treated group to the control group. **b**, Effect of TVA-enriched diet (top) (*n* = 8) or CVA-enriched diet (bottom) (*n* = 10) on B16F10 tumour growth in C57BL/6 mice. **c**, Effect of TVA-enriched diet on B16F10 tumour growth in C57BL/6 mice treated with isotype control (top) or depleting CD8 (bottom) antibodies (*n* = 5). **d**, The percentage of CD4^+^ (control and TVA *n* = 7, CVA *n* = 5) and CD8^+^ (control *n* = 9, TVA *n* = 6, CVA *n* = 4) T cells among intratumoral CD45^+^ cells. **e**, The percentage of CD8^+^ T cells among spleen, dLN and intratumoral CD45^+^ cells (*n* = 8). **f**, PD-1 expression among CD8^+^ T cells in spleen (*n* = 6), dLN (*n* = 3) and tumour (*n* = 4). **g**, Flow cytometry-based quantification of TNF-positive cells among intratumoral CD8^+^ T cells after in vitro stimulation with phorbol myristate acetate (PMA) and ionomycin (*n* = 8). **h**, Quantification of Ki-67^+^ (left), ICOS^+^ (middle) and GZMB^+^ (right) cells among intratumoral CD8^+^ T cells (*n* = 8). **i**, Flow cytometry and quantification of TCF1 expression among intratumoral CD8^+^ T cells (*n* = 8). MFI, mean fluorescence intensity. Data are mean ± s.e.m (**b**,**c**) or mean ± s.d. (**d**–**i**). Two-way ANOVA (**b**,**c**) or Student’s two-sided unpaired *t-*test (**a**,**d**–**i**). Source Data

**Extended Data Fig. 1:**
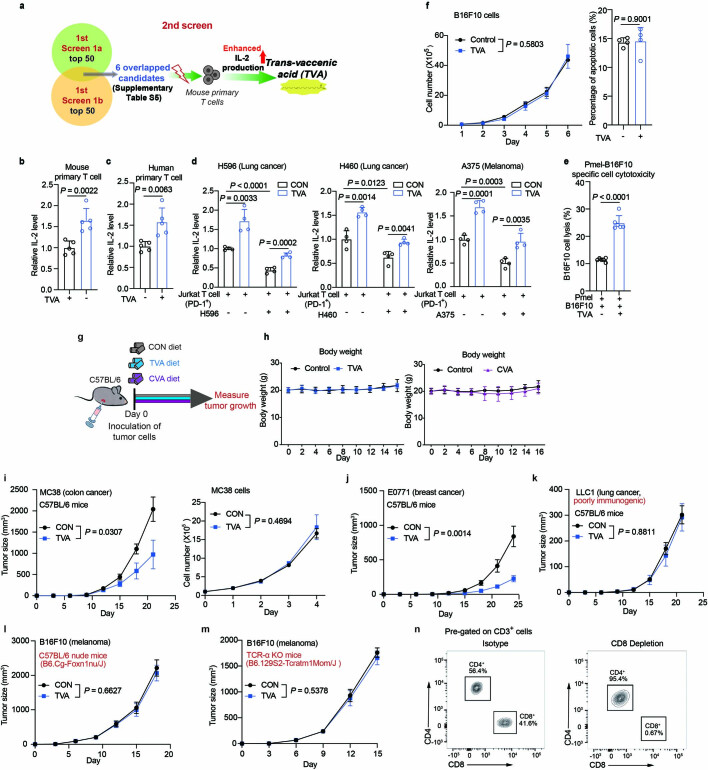
TVA’s anti-tumor immunity effects are mediated through T cells. **a**, The 2^nd^ screen strategy: top 50 candidates from 1^st^ screens 1a and 1b were combined. The resulting 6 overlapped candidates were subjected to analysis of IL-2 levels using mouse primary T cells, revealing TVA as a top candidate that activates T cells. The figure was generated using BioRender. **b**, **c**, Effect of treatment with 20 µM TVA on IL-2 levels in mouse primary T cells (**b**) and human primary T cells (**c**) (*n* = 5). **d**, Effect of treatment with 20 µM TVA on PD-L1/PD-1 mediated Jurkat T cell exhaustion induced by co-cultured H596 (*left*) and H460 (*middle*) human lung cancer cells, or A375 human melanoma cells (*right*), assessed by IL-2 production levels (*n* = 4). **e**, Effect of treatment with 20 µM TVA on cytotoxicity of B16F10 cells by co-cultured Pmel cells in vitro (*n* = 5). **f**, Effect of treatment with 20 µM TVA on B16F10 cell proliferation (*left*) (*n* = 3) or apoptosis (*right*) (*n* = 4). **g**, Schematic depicting experimental design for in vivo mouse model. The figure was generated using BioRender. **h**, Body weight changes of B16F10 tumor-bearing C57BL/6 mice fed with TVA diet (*left*) or CVA diet (*right*) (*n* = 10). **i**, Effect of treatment with 20 µM TVA on MC38 cell proliferation (*left*) (*n* = 3) or TVA-enriched diet on colon cancer MC38 (*right*) (*n* = 8). **j**, **k**, Effect of TVA-enriched diet on breast cancer E0771 (**j**) and lung cancer LLC1 (**k**) tumor growth in C57BL/6 mice (*n* = 8). **l**, **m**, Effect of TVA-enriched diet on B16F10 tumor growth in nude mice (**l**) or TCR-α KO mice (**m**) (*n* = 10). **n**, CD8^+^ T cell depletion efficiency checked by flow cytometry (*n* = 8). Data are mean ± SD (**b**-**f**) or mean ± s.e.m (**h**-**m**). Student’s two-sided unpaired *t* test (**b**-**f**) or two-way ANOVA (**h**-**m**). Source Data

There are around 633 circulating ‘nutrients’ (Supplementary Table 1), including inorganic compounds, organic metabolites, lipids, dietary supplements and proteins. Our blood nutrient library for cell-based screens contains 255 compounds (Supplementary Table 2) that are commercially available. We excluded proteins that have been extensively studied and some supplements that only function at the organismal level, such as fish oils and herbs. The reported physiological range of serum levels and working levels (approximately two times the highest reported serum level) in the screens of individual nutrients are included in Supplementary Table 2. The initial screen (1a) was used to identify nutrients that enhance activation of Jurkat T cells stimulated by CD3 and CD28 antibodies (anti-CD3/CD28) (Fig. 1a, top). Screen 1b was used to identify nutrients that rescue PD-L1–PD-1 dependent exhaustion of Jurkat T cells stably expressing PD-1 induced by co-cultured PD-L1-expressing human H596 lung cancer cells (Fig. 1a, bottom). The results of the two screens are shown in Supplementary Tables 3 and 4. Six overlapping top candidates were further examined in the second screen using mouse primary T cells, and CD4^+^ or CD8^+^ T cells (Extended Data Fig. 1a and Supplementary Table 5). TVA was ranked top and was further confirmed to enhance IL-2 production from mouse and human primary T cells (Extended Data Fig. 1b,c) and rescue PD-L1–PD-1-dependent exhaustion of Jurkat T cells induced by co-cultured PD-L1-expressing human cancer cells (Extended Data Fig. 1d), as indicated by reversed inhibition of IL-2 production.

## Dietary TVA enhances anti-tumour immunity

We found that TVA treatment enhanced the cytotoxicity of mouse melanoma B16F10 cells mediated by co-cultured mouse pmel-1-specific T cells (Extended Data Fig. 1e), whereas TVA did not alter B16F10 cell proliferation or apoptosis (Extended Data Fig. 1f), suggesting that TVA functions through T cells. In addition, the tumour growth potential of immunogenic B16F10 cells was significantly attenuated in syngeneic mice fed with TVA-enriched diet, compared to mice fed with control diet (Fig. 1b, top and Extended Data Fig. 1g). By contrast, the control group fed with either *cis*-vaccenic acid (CVA)-enriched diet or control diet did not show differences in tumour growth potential of B16F10 cells (Fig. 1b, bottom). The TVA and CVA diets did not alter body weights of mice (Extended Data Fig. 1h). Similar results were obtained using immunogenic MC38 colon cancer or E0771 breast cancer cells in syngeneic mice, but not in mice inoculated with poorly immunogenic LLC1 lung cancer cells (Extended Data Fig. 1i–k). Furthermore, TVA diet had minimal effects on B16F10 tumour growth in syngeneic immune-deficient nude mice, and in T cell receptor-α knockout mice (Extended Data Fig. 1l,m), or mice whose CD8^+^ T cells were depleted with CD8α antibodies (Fig. 1c; depletion efficiency shown in Extended Data Fig. 1n). Together, these data indicate that TVA promotes anti-tumour immunity through regulation of CD8^+^ T cells.

## TVA enhances CD8^+^ T cell function

**Extended Data Fig. 2:**
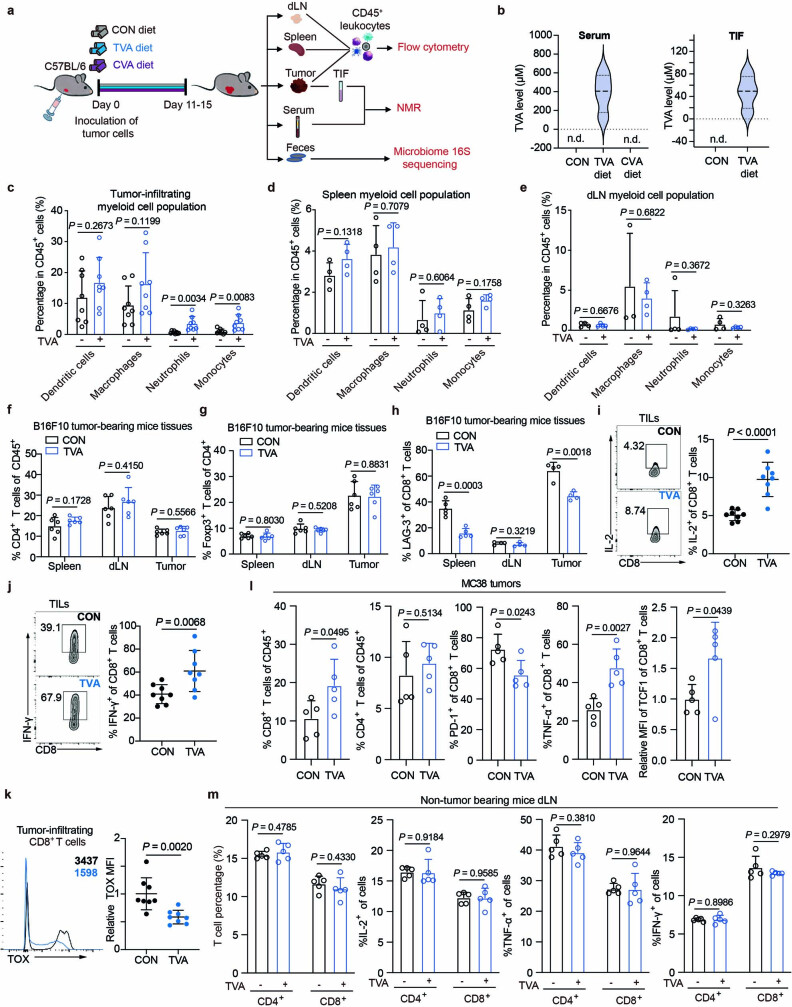
TVA shows distinct effect among leukocytes populations. **a**, Schematic depicting experimental design for in vivo tumor-bearing mouse model fed with indicated diets and samples collection. The figure was generated using BioRender. **b**, TVA levels in B16F10 tumor-bearing mice serum (*left*) and TIF (*right*) were measured by ^1^H nuclear magnetic resonance (NMR) spectroscopy (*n* = 4). **c**, Quantification of the percentage of dendritic cells, macrophages, neutrophils, and monocytes among tumor CD45^+^ cells (*n* = 8). **d**, **e**, Quantification of the percentage of dendritic cells, macrophages, neutrophils, and monocytes among spleen (**d**) or dLN (**e**) CD45^+^ cells (*n* = 4). f, Quantification of the percentage of CD4^+^ T cells among spleen, dLN, and intratumoral CD45^+^ cells (*n* = 6). **g**, Quantification of the percentage of CD4^+^Foxp3^+^ T_reg_ cells among spleen, dLN, and intratumoral CD4^+^ cells (*n* = 6). **h**, Quantification of LAG-3 expression among CD8^+^ T cells in spleen (*n* = 5), dLN, and tumor (*n* = 4). For gating strategies for flow cytometry analysis of spleen and dLN lymphocytes, see Supplementary Fig. 1. **i**, **j**, Flow cytometry and quantification of IL-2 (**i**) and IFN-γ (**j**) levels among intratumoral CD8^+^ T cells after in vitro phorbol myristate acetate (PMA)/ionomycin stimulation (*n* = 8). **k**, Flow cytometry and quantification of TOX expression among intratumoral CD8^+^ T cells (*n* = 8). **l**, Quantification of the percentage of CD8^+^ and CD4^+^ T cells among intratumoral CD45^+^ cells (*left two*), and quantification of PD-1, TNF-α, TCF1 expression among CD8^+^ TILs (*right three*) in MC38 tumors (*n* = 5). **m**, Quantification of the percentage of CD4^+^ and CD8^+^ T cells among dLN CD45^+^ cells (*left*), and quantification of IL-2, TNF-α, IFN-γ expression among dLN CD45^+^ cells (*right three*) in non-tumor-bearing mice (*n* = 5). Data are mean ± SD (**b**-**m**). Student’s two-sided unpaired *t* test (**b**-**m**). Source Data

We next analysed B16F10 tumours 11–15 days after implantation, when tumours were similar in volume (Extended Data Fig. 2a). Quantitative NMR-based measurement revealed that the mean serum TVA concentration is approximately 386 μM, and the mean TVA concentration in tumour interstitial fluid (TIF) is approximately 48 μM (Extended Data Fig. 2b). Flow cytometry analyses revealed that TVA diet, but not control CVA diet, results in an increased CD8^+^ T cell population in tumour-infiltrating lymphocytes (TILs). By contrast, the CD4^+^ T cell population in TILs was not altered (Fig. 1d). TVA diet also markedly increased tumour-infiltrating populations of neutrophils and monocytes, but not dendritic cells or macrophages (Extended Data Fig. 2c). Further, TVA diet did not alter populations of these myeloid cells in spleens or draining lymph nodes (dLNs) (Extended Data Fig. 2d,e).

The TVA diet had no effects on conventional CD4^+^ T cell or CD4^+^FOXP3^+^ regulatory T (T_reg_) cell (Extended Data Fig. 2f,g) populations in spleens, dLNs and tumours from B16F10 tumour-bearing mice. By contrast, TVA diet resulted in a larger percentage of CD8^+^ T cells as a fraction of the CD45^+^ leukocytes infiltrated in B16F10 tumours and dLNs but not spleens (Fig. 1e). Dietary TVA reduced exhaustion of CD8^+^ T cells in tumours and spleens, but not in dLNs, as indicated by decreased expression levels of the exhaustion markers PD-1 (Fig. 1f) and LAG-3 (Extended Data Fig. 2h). Further analysis of tumour-infiltrating CD8^+^ T cells with additional representative markers revealed that dietary TVA promotes CD8^+^ T cell function with increased levels of cytokines including IL-2, IFNγ (Extended Data Fig. 2i,j) and TNF (Fig. 1g), the proliferation marker Ki-67, the co-stimulatory receptor ICOS and the cytolytic molecule GZMB (Fig. 1h), as well as the stem-like CD8^+^ T cell survival marker TCF1 (ref. ^14^) (Fig. 1i) but reduced expression level of the exhaustion marker TOX (Extended Data Fig. 2k). Similar results were obtained using tumour-infiltrating CD8^+^ T cells isolated from MC38 tumours (Extended Data Fig. 2l). By contrast, the TVA diet had no effects on CD4^+^ and CD8^+^ T cell population and function in non-tumour-bearing mice (Extended Data Fig. 2m), suggesting that the effects of dietary TVA on T cells probably depends on the induction of immune responses. Together, these results suggest that dietary TVA promotes accumulation and function of tumour-infiltrating CD8^+^ T cells.

**Extended Data Fig. 3:**
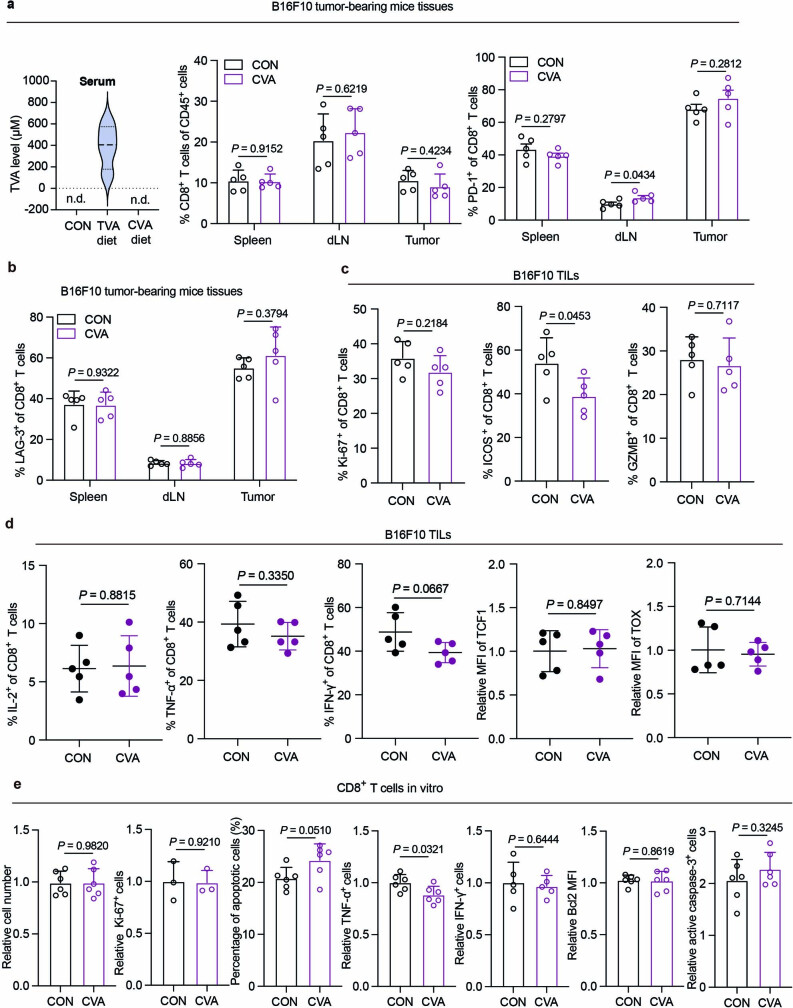
Dietary CVA has no significant effect on CD8^+^ T cell function in tumor-bearing mice. **a**, With CVA diet, TVA levels in B16F10 tumor-bearing mice serum (*left*) were measured by ^1^H nuclear magnetic resonance (NMR) spectroscopy (*n* = 4). Quantification of the percentage of CD8^+^ T cells among spleen, dLN, and intratumoral CD45^+^ cells (*n* = 5) (*middle*). Quantification of PD-1 expression among CD8^+^ T cells in spleen, dLN, and tumor (*n* = 5) (*right*). **b**, Quantification of LAG-3 expression among CD8^+^ T cells in spleen, dLN, and tumor (*n* = 5). **c**, Quantification of Ki-67- (*left*), ICOS- (*middle*), and GZMB-positive cells (*right*) among intratumoral CD8^+^ T cells (*n* = 5). **d**, Flow cytometry-based quantification of IL-2, TNF-α, or INF-γ-positive cells among intratumoral CD8^+^ T cells after in vitro phorbol myristate acetate (PMA)/ionomycin stimulation (*n* = 5, *left three*), flow cytometry and quantification of TCF1 and TOX expression among intratumoral CD8^+^ T cells (*n* = 5, *right two*). **e**, Mouse primary CD8^+^ T cells were isolated, activated, and treated with CVA, followed by analysis of CVA effect on cell functions. Relative cell number (*n* = 5), Ki-67-positive cells (*n* = 3), percentage of apoptotic cells (*n* = 6), relative TNF-α- (*n* = 6) or IFN-γ-positive cells (*n* = 5), Bcl2 level, and active caspase-3 level among CD8^+^ T cells (*n* = 6) were shown. Data are mean ± SD (**a**-**e**). Student’s two-sided unpaired *t* test (**a**-**e**). Source Data

**Extended Data Fig. 4:**
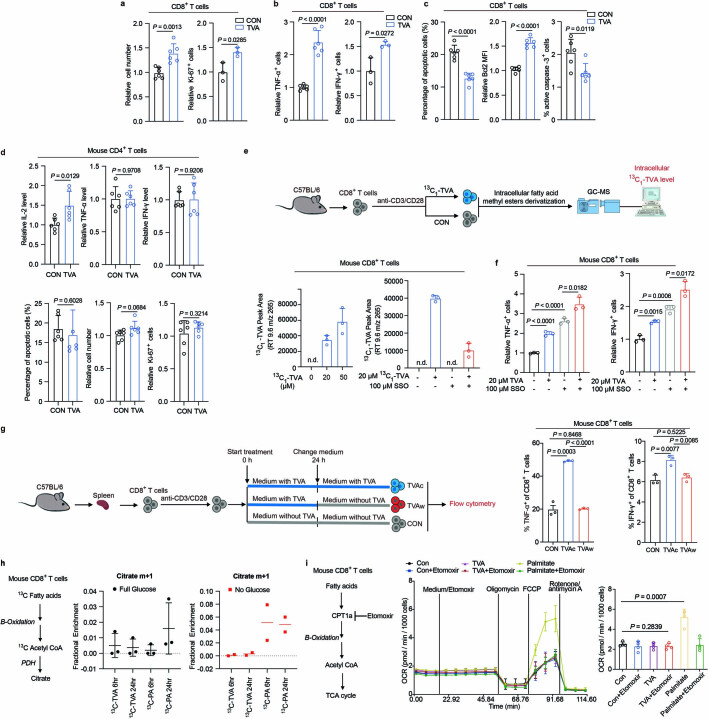
TVA functions in an extracellular manner. **a**, Relative cell number (*n* = 6) and Ki-67-positive cells among CD8^+^ T cells +/− TVA treatment (*n* = 3). **b**, Relative TNF-α- (*left*) (*n* = 6) or IFN-γ-positive cells (*right*) (*n* = 3) among CD8^+^ T cells +/− TVA treatment. **c**, Percentage of apoptotic cells (*left*), Bcl2 level (*middle*), and active caspase-3 level (*right*) among CD8^+^ T cells +/− TVA treatment (*n* = 6). **d**, Relative IL-2, TNF-α, and IFN-γ production among CD4^+^ T cells after phorbol myristate acetate (PMA)/ionomycin stimulation + /− TVA treatment in vitro (*upper three*), and apoptotic cell percentage among CD4^+^ T cells, relative cell number (*n* = 5), Ki-67-positive cells (*lower three*) (*n* = 6). **e**, Schematic depicting experimental setup for in vitro T cell model for ^13^C_1_-TVA metabolic flux analysis by GC-MS. The figure was generated using BioRender (*upper*). Detection of intracellular ^13^C-labled TVA level in mouse CD8^+^ T cells by GC-MS (*lower left*). Effect of CD36 inhibitor SSO on ^13^C-labled TVA uptake in mouse CD8^+^ T cells was accessed by GC-MS (*lower right*) (*n* = 3). **f**, Effect of TVA treatment with or without CD36 inhibitor SSO on production of TNF-α (*left*) or INF-γ (*right*) by mouse CD8^+^ T cells (*n* = 3). **g**, Schematic depicting experimental setup for in vitro T cell model for TVA “wash-off” assay (TVAc: CD8^+^ T cells were treated with 20 µM TVA for 48 h; TVAw: CD8^+^ T cells were treated with 20 µM TVA for 24 h and then changed to culture medium without TVA for another 24 h). The figure was generated using BioRender (*left*). Quantification of TNF-α- (*middle*) and IFN-γ-positive cells (*right*) after phorbol myristate acetate (PMA)/ionomycin stimulation among CD8^+^ T cells for TVA “wash-off” assay (*n* = 3). **h**, ^13^C fatty acid tracing experiment: Schematic illustrating routes of ^13^C entry from ^13^C-fatty acids (*left*), citrate enrichment in mouse CD8^+^ T cells after treated with ^13^C -TVA or ^13^C -palmitate acid (PA) under full glucose medium (*middle*) (*n* = 3), or under no glucose medium (*right*) (*n* = 2). **i**, Fatty acid oxidation measured by Seahorse: Schematic of fatty acid oxidation (FAO) in mouse CD8^+^ T cells (*left*), the impact of TVA or PA treatment on FAO via assessing changes in oxygen consumption (OCR) (*middle*), quantification of OCR (*right*) (*n* = 4). Data are mean ± SD (**a-h** *middle*, **i**). Student’s two-sided unpaired *t* test (**a**-**h** *middle*, **i**). Source Data

By contrast, CVA diet did not alter the circulating TVA levels in mice (Extended Data Fig. 3a, left), and CVA diet in vivo or CVA treatment in vitro had minimal effects on CD8^+^ T cell function (Extended Data Fig. 3a–e). Consistent with these findings, anti-CD3/CD28-stimulated primary CD8^+^ T cells treated with TVA in vitro increased in cell number, with increased expression of Ki-67, TNF and IFNγ (Extended Data Fig. 4a,b), but decreased apoptosis, increased BCL-2 expression and decreased active caspase-3 levels (Extended Data Fig. 4c). By contrast, TVA-treated primary CD4^+^ T cells in vitro showed increased IL-2 production but unaltered production of effector molecules including TNF and IFNγ, proliferation and apoptosis (Extended Data Fig. 4d). Collectively, these results suggest that TVA selectively enhances function of stimulated CD8^+^ T cells.

## TVA signals through a GPCR–CREB axis

We next determined whether TVA functions in an extracellular or intracellular manner. Quantitative mass spectrometry detected isotope-labelled [^13^C]TVA imported from the medium to mouse primary CD8^+^ T cells in a dose-dependent manner, whereas the CD36 inhibitor sulfosuccinimidyl oleate (SSO) reduced import of [^13^C]TVA (Extended Data Fig. 4e). However, SSO treatment did not affect TVA-enhanced CD8^+^ T cell activation (Extended Data Fig. 4f), and withdrawal of TVA from the culture medium abolished TVA-enhanced CD8^+^ T cell activation, suggesting that the effects of TVA are probably extracellular and reversible (Extended Data Fig. 4g). We then performed an isotope-tracing experiment to examine whether intracellular [^13^C]TVA is broken down by fatty acid β-oxidation and incorporated in citrate (Extended Data Fig. 4h). When primary mouse CD8^+^ T cells were cultured with full glucose and treated with [^13^C]TVA, there was no labelling of [^13^C]TVA to citrate, whereas ^13^C labelling of citrate was detected in cells treated with control [^13^C]palmitate. Similar results were obtained using cells under a glucose-deprivation condition. We also tested whether intracellular TVA is broken down to participate the TCA cycle (Extended Data Fig. 4i). TVA treatment did not alter the oxygen consumption rate (OCR) of CD8^+^ T cells in the presence or absence of the CPT1a inhibitor etomoxir, whereas the control palmitic acid treatment resulted in increased OCR that was abolished by etomoxir. Together, these data indicate that TVA primarily functions outside of CD8^+^ T cells, whereas intracellular TVA is not broken down for CD8^+^ T cell metabolism.

**Fig. 2:**
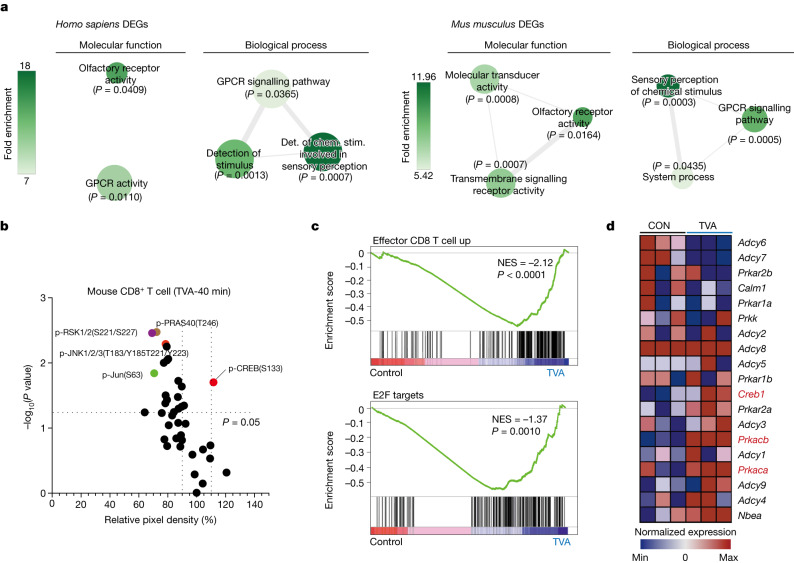
TVA has a regulatory function through a GPCR–CREB axis. **a**, Human or mouse primary CD8^+^ T cells were isolated, activated and treated with or without TVA for different durations, followed by integrated, temporal mechanistic studies. GO term enrichment graphs generated from KAS-seq differential analysis of *H. sapiens* (left) and *M. musculus* (right) CD8^+^ T cell gene bodies (treated with 20 μM TVA versus untreated). Specifically, gene bodies exhibiting differential single-stranded DNA (ssDNA) levels for all timepoints (cut-off for individual timepoints of *P* < 0.4 (*H. sapiens*) or *P* < 0.5 (*M. musculus*)) are shown. Colour indicates fold-enrichment and size of GO term circles denotes the number of differentially expressed genes (DEGs) from KAS-seq data for that term. *n* = 3. Det. of chem. stim., determination of chemical stimulation. **b**, Scatter plot of a phospho-antibody array representing relative pixel density after TVA treatment for 40 min versus corresponding −log_10_(*P* value). Phosphoproteins with relative pixel density greater than 110% or less than 90% with *P* < 0.05 are highlighted (*n* = 3). **c**, GSEA of upregulated effector CD8^+^ T cells (top) and E2F targets (bottom) induced by TVA treatment in CD8^*+*^ T cells (*n* = 3). NES, normalized enrichment score. **d**, Heat map showing relative expression of PKA–CREB pathway genes in CD8^+^ T cells comparing TVA treatment group to the control ones (*n* = 3). Student’s two-sided unpaired *t*-test (**b**). Nominal *P* values were adjusted by the Benjamini–Hochberg method (**a**,**c**).

**Extended Data Fig. 5:**
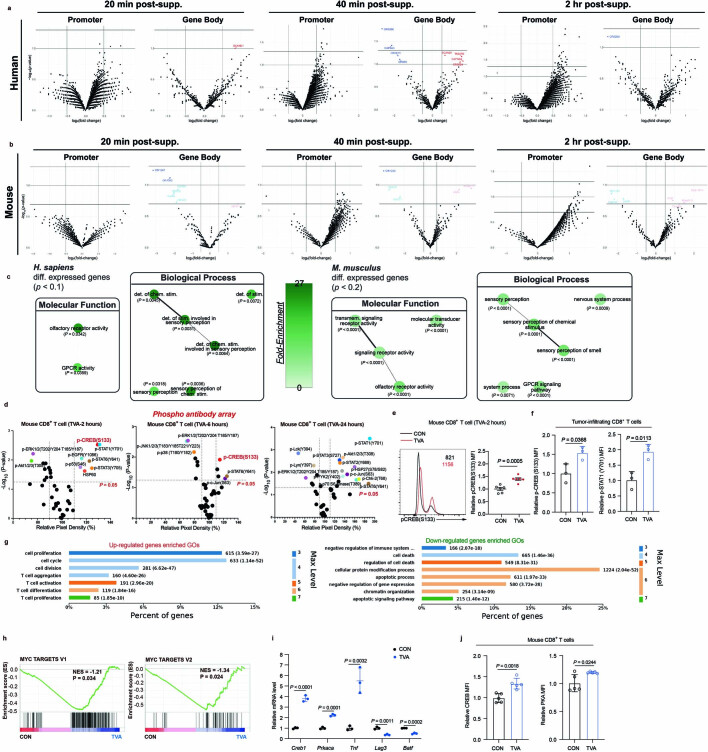
TVA activates a GPCR pathway in CD8^+^ T cells. **a**, **b**, Human (**a**) and mouse (**b**) Volcano plots depicting differential promoter and gene body ssDNA levels measured by KAS-seq in CD8^+^ T cell treated with 20 μM TVA for indicated timepoint (relative to untreated cells). Heavy horizontal lines correspond to p-values of 0.2 (*M. musculus* only), 0.1, and 0.05, while heavy vertical lines correspond to a log_2_|fold-change| value of 0.5 (*n* = 3). **c**, GO enrichment graphs for all differentially expressed gene bodies (at any timepoint) for the corresponding *P*-value cutoffs and species. Color and GO term circle size correspond to fold-enrichment and number of differentially-expressed gene bodies for that term, respectively (*n* = 3). **d**, Scatterplot of phosphor-antibody array representing relative pixel density after TVA treatment for indicated time versus corresponding −log_10_(P-value). Phosphor-proteins with relative pixel density >110% or <90%, meanwhile *P* < 0.05 as significant change were highlighted (*n* = 3). **e**, Flow cytometry (*left*) and quantification (*right*) of p-CREB(S133) level among CD8^+^ T cells treated with 20 µM TVA for 2 h (*n* = 6). **f**, Quantification of p-CREB (*left*), and p-STAT1 (*right*) levels among mouse tumor-infiltrating CD8^+^ T cells (*n* = 3). **g**, GO enrichment analysis of up-regulated genes (*upper*) and down-regulated genes (*lower*) by RNA-seq analysis of CD8^+^ T cells treated with TVA comparing to control (*n* = 3). **h**, GSEA of upregulated MYC targets V1 (*left*) and MYC target V2 (*right*) induced by TVA treatment in CD8^+^ T cells. NES, normalized enrichment score. **i**, Validation of some differentially expressed genes (DEGs) from RNA-seq analysis by RT-PCR (*n* = 3). **j**, Quantification of CREB (*left*), and PKA (*right*) levels in mouse CD8^+^ T cells treated with or without TVA (*n* = 5). Data are mean ± SD (**e**-**f**, **i**-**j**). Nominal P values were adjusted by the Benjamini–Hochberg method (**a**-**c**, **h**). Student’s two-sided unpaired *t* test (**d**-**f**, **i**-**j**). Source Data

We investigated the effects of TVA on human or mouse primary CD8^+^ T cells using integrated, temporal mechanistic studies, including: (1) the kethoxal-assisted single-stranded DNA sequencing (KAS-seq) approach^15^ to identify the initial (20 min–2 h) effect of TVA on cells by capturing global transcription dynamics; (2) phospho-antibody array to identify early (40 min–24 h) cellular signalling changes; and (3) the RNA-sequencing (RNA-seq) approach (at 24 h), for whole-transcriptome analysis. Functional enrichment of the top-ranking altered gene bodies from the genome-scale KAS-seq results (Extended Data Fig. 5a,b) revealed GPCR activities among the top-enriched ontologies in TVA-treated CD8^+^ T cells (Fig. 2a and Extended Data Fig. 5c). Consistent with this finding, we observed increased phosphorylation of the transcription factor CREB—a common downstream effector of GPCRs^16^—as early as 40 min after TVA treatment (Fig. 2b and Extended Data Fig. 5d; full phospho-antibody array data in Supplementary Table 6). We confirmed the increased phosphorylated CREB (p-CREB) level in mouse primary CD8^+^ T cells treated with TVA by flow cytometry analysis (Extended Data Fig. 5e). Phosphorylation levels of STAT1—which is important for efficient expansion of CD8^+^ T cells^17^—were also increased by TVA. Increased p-CREB and p-STAT1 levels in tumour-infiltrating CD8^+^ T cells in B16F10 tumours from syngeneic mice fed on TVA were confirmed by flow cytometry analysis (Extended Data Fig. 5f).

In addition, Gene Ontology (GO) enrichment analysis^18,19^ and global gene set enrichment analysis^20^ (GSEA) identified a set of upregulated signalling pathways that are responsible for the effects of TVA treatment on CD8^+^ T cells at 24 h, including T cell proliferation and activation, and a set of downregulated signalling pathways, including apoptosis (Extended Data Fig. 5g). Notably, TVA treatment enhanced expression of the genes enriched in effector CD8^+^ T cell function, E2F (Fig. 2c) and MYC pathways (Extended Data Fig. 5h), correlating with enhanced CD8^+^ T cell function and proliferation. TVA treatment also upregulated the expression of *Creb1* (which encodes CREB) and *Prkacb* and *Prkaca* (which encodes the catalytic subunits α and β, respectively, of cAMP-dependent protein kinase A (PKA)) (Fig. 2d), with increased mRNA and protein levels (Extended Data Fig. 5i,j), and *Tnf*, and downregulated *Lag3* and *Batf*, which are associated with T cell exhaustion (Extended Data Fig. 5i). Collectively, these results suggest that enhanced CD8^+^ T cell function induced by TVA is mediated through a GPCR–CREB pathway, with positive feedback augmenting expression of PKA and CREB at the gene level.

## TVA activates the cAMP–PKA–CREB pathway

**Extended Data Fig. 6:**
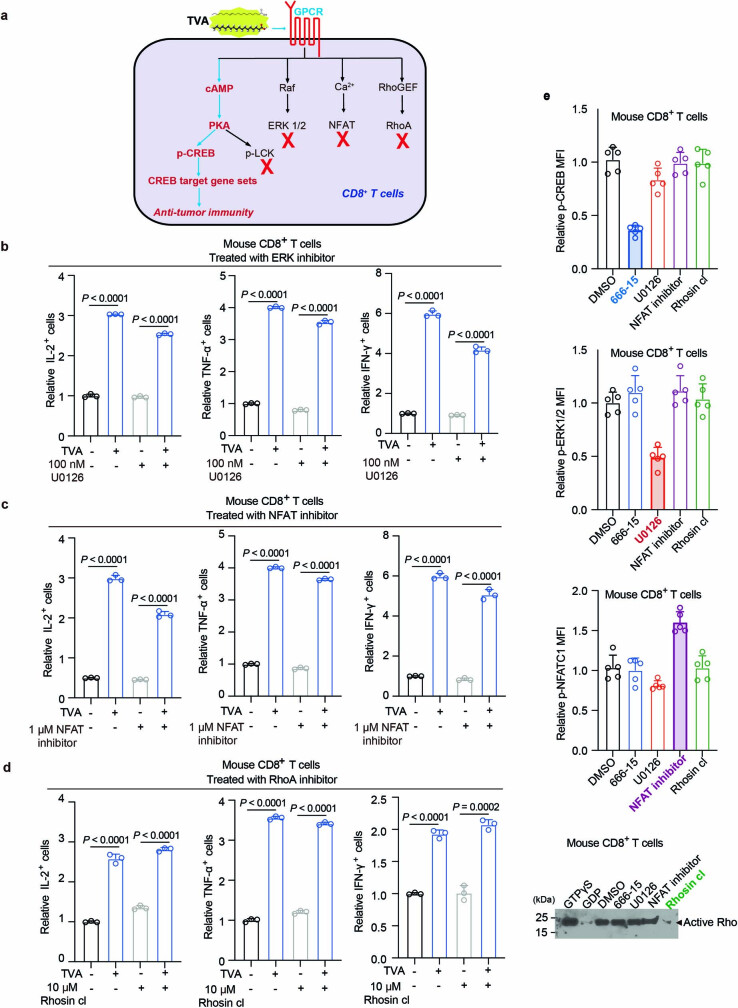
TVA functions through the cAMP-PKA-CREB axis but not other downstream effector pathways of GPCR. **a**, Summary of TVA-GPCR downstream signaling changes induced by TVA in CD8^+^ T cells. The red fork means no change. **b**, **c**, **d**, Effect of treatment with ERK inhibitor U0126 (**b**), NFAT inhibitor (**c**), or RhoA inhibitor Rhosin cl (**d**) on relative IL-2 (*left*), TNF-α (*middle*), and IFN-γ (*right*) levels in mouse CD8^+^ T cells treated with or without TVA (*n* = 3). **e**, Validation of CREB, ERK, NFAT, and RhoA inhibitors effects at the concentrations used, GTPγS (positive control) and GDP (negative control) were used to ensure the pull-down procedures worked properly (*n* = 5). For gel source data, see Supplementary Fig. 2. Data are mean ± SD (**b**-**e**). Student’s two-sided unpaired *t* test (**b**-**e**). Source Data

**Extended Data Fig. 7:**
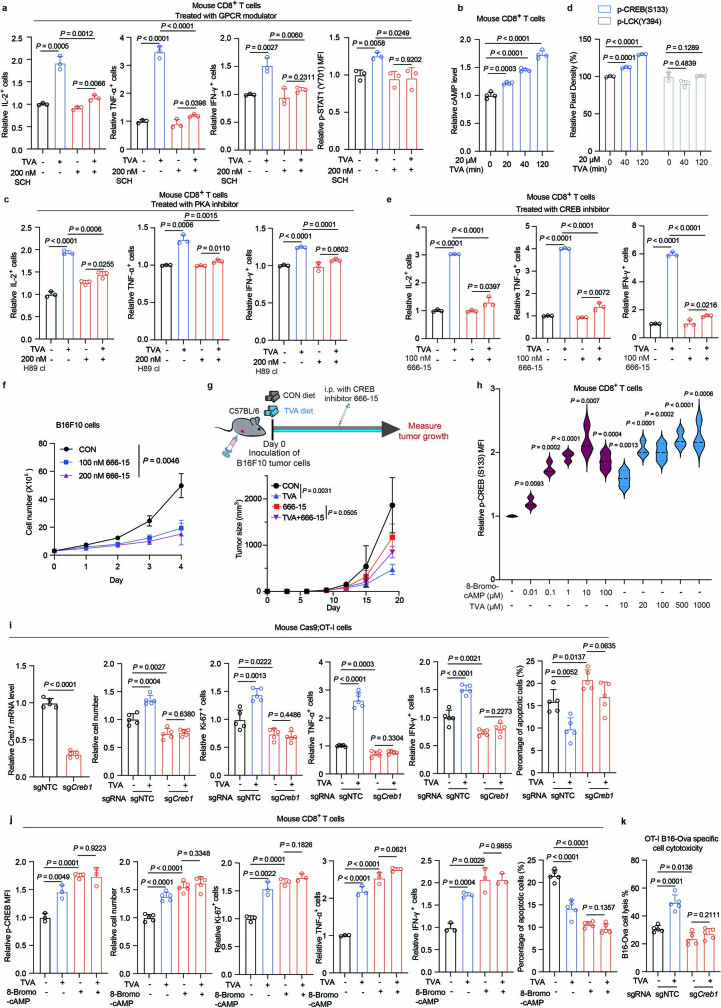
TVA requires the cAMP-PKA-CREB axis to enhance CD8^+^ T cell function. **a**, Effects of treatment with GPCR modulator SCH on TVA-dependent CD8^+^ T cell activation assessed by IL-2 (*left*), TNF-α (*middle left*), IFN-γ (*middle right*), and p-STAT1 (*right*) levels (*n* = 3). **b**, Effects of TVA treatment on cAMP levels of CD8^+^ T cells (*n* = 3). **c**, Effect of treatment with PKA inhibitor H89 cl on TVA-dependent CD8^+^ T cell activation assessed by IL-2 (*left*), TNF-α (*middle*), and IFN-γ (*right*) levels (*n* = 3). **d**, Effect of TVA treatment on p-CREB and p-LCK levels of CD8^+^ T cells (*n* = 3). **e**, Effects of treatment with CREB inhibitor 666-15 on TVA-dependent CD8^+^ T cell activation assessed by IL-2 (*left*), TNF-α (*middle*), and IFN-γ (*right*) levels (*n* = 3). **f**, Effect of treatment with CREB inhibitor 666-15 on B16F10 cell proliferation in vitro (*n* = 3). **g**, Schematic depicting experimental design for in vivo tumor-bearing mouse model fed with TVA diet and treated with CREB inhibitor. The figure was generated using BioRender (*upper*). Effects of treatment with CREB inhibitor 666-15 and / or TVA diet on B16F10 tumor growth in vivo (*lower*) (*n* = 5). **h**, Effect of treatment with different doses of cell permeable 8-Bromo-cAMP or TVA on p-CREB levels of CD8^+^ T cells (*n* = 3). **i**, Effect of knockout of *Creb1* with sgRNA on *Creb1* mRNA level, relative cell number, Ki67 level, TNF-α level, IFN-γ level, and apoptotic cell percentage in mouse Cas9;OT-I cells treated with or without TVA (*n* = 5). **j**, Effect of 8-Bromo-cAMP treatment on p-CREB level (*n* = 3), relative cell number (*n* = 5), Ki67 level (*n* = 3), TNF-α level (*n* = 3), IFN-γ level (*n* = 3), and apoptotic cell percentage in mouse CD8^+^ T cells treated with or without TVA (*n* = 5). **k**, Effect of knockout of *Creb1* with sgRNA on cell cytotoxicity in mouse Cas9;OT-I cells treated with or without TVA (*n* = 5). Data are mean ± SD (**a**-**e** and **h**-**k**) or mean ± s.e.m (**f**-**g**). Student’s two-sided unpaired *t* test (**a**-**e** and **h**-**k**) or two-way ANOVA (**f**-**g**). Source Data

We next sought to determine which pathways downstream of GPCRs are required for TVA function (the results are summarized in Extended Data Fig. 6a). Specific inhibitors targeting downstream effectors of GPCRs, including MEK1, NFAT and RHOA (Extended Data Fig. 6b–d) did not alter TVA-dependent CD8^+^ T cell activation. Effectiveness and selectivities of the inhibitors are shown in Extended Data Fig. 6e. By contrast, treatment with SCH-202676 hydrobromide, a sulphydryl-reactive compound that blocks agonist and antagonist binding to GPCRs, abolished TVA-enhanced activation of CD8^+^ T cells (Extended Data Fig. 7a). Consistent with this finding, TVA treatment increased levels of cAMP in CD8^+^ T cells (Extended Data Fig. 7b), and treatment with the PKA inhibitor H-89 abolished TVA-enhanced activation of CD8^+^ T cells (Extended Data Fig. 7c).

Downstream PKA effectors include CREB^21^ and the lymphocyte-specific tyrosine kinase LCK^22^, that are reported to positively and negatively regulate T cell activation, respectively. We found that TVA treatment promotes phosphorylation of CREB but not LCK (Extended Data Fig. 7d). In addition, treatment with the CREB inhibitor 666-15 markedly reduced TVA-enhanced activation of CD8^+^ T cells (Extended Data Fig. 7e). Next, we found that treatment with 666-15 alone reduced B16F10 cell proliferation and tumour growth (Extended Data Fig. 7f,g), and combined treatment with 666-15 and TVA diet resulted in a significant rescue of tumour growth potential of B16F10 syngeneic mice compared with mice treated with TVA diet alone to a similar level to that of mice treated with 666-15 alone (Extended Data Fig. 7g). These results suggest that CREB inhibition antagonizes the effects of dietary TVA on anti-tumour immunity.

Furthermore, treatment with cell-permeable 8-bromo-cAMP increased the amount of p-CREB in CD8^+^ T cells in a dose-dependent manner, similar to TVA (Extended Data Fig. 7h). CRISPR–Cas9-mediated knockout of CREB in OT-I cells attenuated TVA-enhanced cell proliferation, Ki-67 level, and TNF and IFNγ production, and attenuated the reduction in apoptosis in response to TVA (Extended Data Fig. 7i). Similarly, activation of mouse primary CD8^+^ T cells by 8-bromo-cAMP could not be further altered by TVA (Extended Data Fig. 7j). In addition, as shown in Extended Data Fig. 7k, *Creb1* knockout in OT-I cells abolished the TVA-enhanced cytotoxic effects of OT-I cells on co-cultured B16-OVA cells.

## The effects of TVA require CREB

**Fig. 3:**
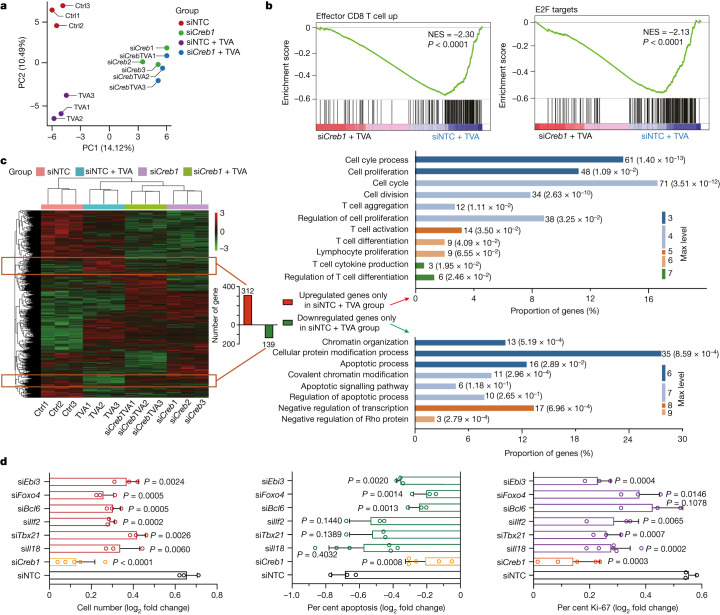
TVA’s effect on CD8^+^ T cells is primarily mediated through CREB and its target gene sets. **a**, Principal component analysis of genes from RNA-seq analysis of siRNA-mediated transient knockdown of *Creb1* (si*Creb1*) in CD8^+^ T cells with or without TVA treatment, compared to non-targeting control siRNA (siNTC) (*n* = 3). **b**, GSEA of upregulated effector CD8^+^ T cells (left) and E2F targets (right) with siNTC and TVA treatment compared with si*Creb1* and TVA treatment group. *n* = 3. Nominal *P* values were adjusted by the Benjamini–Hochberg method. **c**, Heat map of differentially expressed genes from RNA-seq analysis of si*Creb1* CD8^+^ T cells comparing with siNTC with or without TVA treatment. The up- or downregulated genes in the siNTC plus TVA group compared to the other three groups were gated with orange boxes (left) and enriched for GO analysis (right) (*n* = 3). **d**, *Creb1* target genes validation. log_2_ fold changes of cell number (left), apoptosis (middle) and Ki-67 expression (right) after TVA treatment in CD8^+^ T cells with individually transient knockdown of *Creb1* (*n* = 5), *Il18* (left, *n* = 3; middle and right, *n* = 6), *Tbx21*, *Ilf2*, *Bcl6*, *Foxo4* and *Ebi3* (*n* = 3). Data are mean ± s.d. Student’s two-sided unpaired *t*-test. Ctrl, control. Source Data

**Extended Data Fig. 8:**
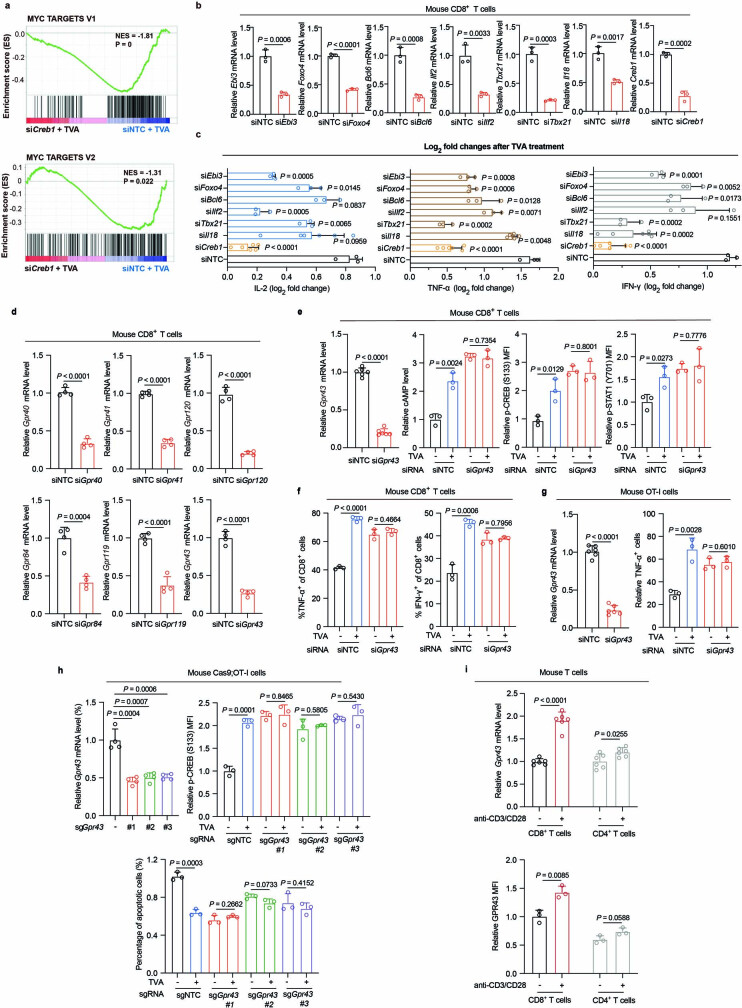
TVA-enhanced CD8^+^ T cell function requires the GPR43-CREB axis. **a**, GSEA of upregulated MYC targets V1 (*upper*) and MYC target V2 (*lower*) in siNTC with TVA treatment compared to si*Creb1* with TVA treatment. NES, normalized enrichment score. **b**, *Ebi3, Foxo4, Bcl6, Ilf2, Tbx21, IL18, and Creb1* knockdown efficiency checked by RT-PCR (*n* = 3). **c**, TVA-*Creb1* target genes validation: Log2 fold changes of IL-2 (*left*), TNF-α (*middle*), and IFN-γ (*right*) after TVA treatment in CD8^+^ T cells with individually transient knockdown of *Creb1*, *Il18* (*n* = 6), *Tbx21*, *Ilf2*, *Bcl6*, *Foxo4*, and *Ebi3* (*n* = 3). **d**, *Gpr40*, *Gpr41*, *Gpr120*, *Gpr84*, *Gpr119*, and *Gpr43* knockdown efficiency checked by RT-PCR (*n* = 4). **e**, *Gpr43* knockdown efficiency in CD8^+^ T cells was checked by RT-PCR (*left*) (*n* = 6). Effects of *Gpr43* transient knockdown on cAMP (*middle left*), p-CREB (*middle right*), and p-STAT1 (*right*) levels in mouse CD8^+^ T cells treated with or without TVA were shown (*n* = 3). **f**, Effects of *Gpr43* transient knockdown on TNF-α (*left*) and IFN-γ (*right*) levels in mouse CD8^+^ T cells treated with or without TVA (*n* = 3). **g**, *Gpr43* knockdown efficiency in OT-I cells was checked by RT-PCR (*left*). Effect of *Gpr43* transient knockdown on TNF-α level (*right*) in OT-I cells treated with or without TVA was shown (*n* = 3). **h**, Knockout efficiency of *Gpr43* in Cas9;OT-I cells was checked by RT-PCR (*upper left*) (*n* = 4). Effects of individually knockout of *Gpr43* with three sgRNAs on p-CREB level (*upper right*) and apoptosis (*lower*) in mouse Cas9;OT-I cells treated with or without TVA were shown (*n* = 3). **i**, Relative *Gpr43* mRNA level (*n* = 6) and protein level (*n* = 3) in CD8^+^ T cells and CD4^+^ T cells with or without anti-CD3/CD28 stimulation. Data are mean ± SD (**b**-**i**). Nominal P values were adjusted by the Benjamini–Hochberg method (**a**), Student’s two-sided unpaired *t* test (**b**-**i**). Source Data

We performed transcriptome-wide RNA-seq using CD8^+^ T cells with *Creb1-*knockdown control cells treated with non-targeting control (siNTC) short inhibitory RNA (siRNA) in the presence and absence of TVA. Principal component analysis revealed that cells treated with siRNA targeting Creb (si*Creb1*) in the presence and absence of TVA can be grouped together and are separated from the siNTC control cell group, or the group of control cells treated with TVA (Fig. 3a). GSEA analysis revealed that knockdown of CREB reverses TVA-dependent upregulation of gene sets related to effector CD8^+^ T cell function, and cell proliferation including E2F (Fig. 3b) and MYC pathways (Extended Data Fig. 8a).

To further identify targets downstream of TVA–CREB, we characterized genes that were upregulated or downregulated only in the siNTC plus TVA group compared with three other groups (Fig. 3c, left; full gene list is shown in Supplementary Table 7). In total, 312 upregulated genes were enriched in 11 GO categories, including cell cycle, cell proliferation, cell division, T cell aggregation, T cell activation, T cell differentiation and cytokine production (Fig. 3c, top right). By contrast, a total of 139 downregulated genes were enriched in 8 GO categories, including apoptosis and chromatin organization (Fig. 3c, bottom right). For functional validation, we selected four upregulated genes that are critical in cell proliferation and T cell function. These genes include *Il18* (associated with cell proliferation, T cell activation and cytokine production), *Ebi3* (associated with cell proliferation and T cell activation), *Tbx21*(associated with of T cell activation and cytokine production) and *Ilf2* (associated with cell proliferation, T cell activation and cytokine production). We also tested *Foxo4* and *Bcl6*, which are critical genes in apoptosis-related GO categories. Each of these six TVA–CREB target genes was knocked down by distinct siRNA probes in mouse CD8^+^ T cells (knockdown efficiency shown in Extended Data Fig. 8b), and in functional studies upon TVA treatment.

As shown in Fig. 3d and Extended Data Fig. 8c, knockdown of *Creb1* expression by si*Creb1* effectively reverses TVA-dependent changes in cell number and apoptosis, and levels of Ki-67, IL-2, TNF and IFNγ in CD8^+^ T cells, compared with control cells treated with siNTC. Knockdown of *Il18*, *Ebi3*, *Tbx21*, *Ilf2*, *Foxo4* or *Bcl6* partially reversed the TVA-dependent change in cell number (Fig. 3d, left). Knockdown of *Bcl6*, *Foxo4* or *Ebi3* partially reversed the TVA-dependent change in the apoptotic population of CD8^+^ T cells, whereas knockdown of *Il18*, *Tbx21* or *Ilf2* had no such effect (Fig. 3d, middle). Knockdown of *Il18*, *Tbx21*, *Ilf2* or *Ebi3* partially reversed TVA-dependent change of Ki-67 level, whereas knockdown of *Bcl6* or *Foxo4* did not have this effect (Fig. 3d, right). Knockdown of *Foxo4, Tbx21*, *Ilf2* or *Ebi3* partially reversed the TVA-dependent change in IL-2 level, whereas knockdown of *Bcl6* or *Il18* did not (Extended Data Fig. 8c, left). Knockdown of each of the six genes partially reversed the TVA-dependent change in TNF level (Extended Data Fig. 8c, middle). Finally, knockdown of *Bcl6*, *Il18, Foxo4, Tbx21* or *Ebi3* partially reversed the TVA-dependent change in IFNγ level, whereas knockdown of *Ilf2* had no effect (Extended Data Fig. 8c, right). Together, these results establish differentially functional contributions of diverse CREB target genes to distinct TVA-dependent changes in CD8^+^ T cells.

## TVA inactivates SCFA-binding GPR43

**Fig. 4:**
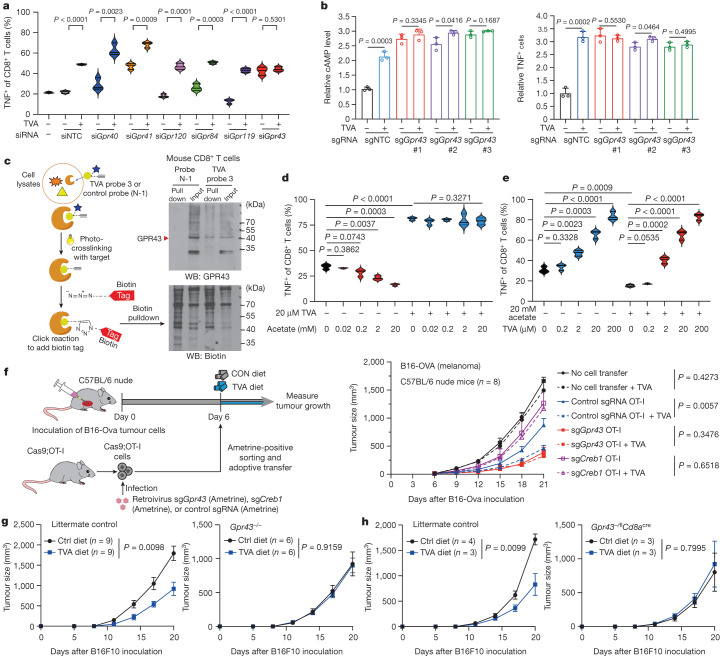
TVA inactivates SCFA-binding GPR43. **a**, Effects of knockdown of known fatty acid-binding GPCRs on TNF expression in mouse CD8^+^ T cells with or without TVA (*n* = 3). **b**, Effects of knockout of *Gpr43* with three different sgRNAs on cAMP level (left) and TNF expression (right) in mouse Cas9-expressing OT-I cells with or without TVA (*n* = 3). **c**, Schematic of experimental setup for the pull-down assay (left). Proteins labelled with biotin by control probe N-1 or TVA probe 3 were pulled down by streptavidin beads. Western blotting was performed with anti-GP43 (top right) and anti-biotin (bottom right) on biotin-labelled proteins (*n* = 2). Gel source data is shown in Supplementary Fig. 2. **d**, Effects of different doses of acetate on TNF expression in mouse CD8^+^ T cells with or without 20 µM TVA (*n* = 3). **e**, Effects of increasing concentrations of TVA on TNF expression in CD8^+^ T cells with or without 20 mM acetate (*n* = 3). **f**, Left, schematic of experimental design for *Gpr43*- or *Creb1*-knockout Cas9-expressing OT-I cells in adoptive cell therapy experiments. This schematic was generated using BioRender.com. Cas9-expressing OT-I cells transduced with non-targeting control sgRNA or sgRNA targeting *Gpr43* or *Creb1* were transferred into mice at day 6 after engraftment of B16-OVA followed by analyses of tumour size (right). Non-treatment control mice did not receive T cells (*n* = 8). **g**, Effect of TVA-enriched diet on B16F10 tumour growth in *Gpr43*^−/−^ (*n* = 6) or littermate control (*n* = 8) mice. **h**, Effects of TVA-enriched diet on B16F10 tumour growth in *Gpr43*^*−/fl*^*Cd8a*^*cre*^ (*n* = 3) or littermate control (control diet *n* = 4; TVA diet *n* = 3) mice are shown. Data are mean ± s.d. (**a**,**b**,**d**,**e**) or mean ± s.e.m. (**f**–**h**). Student’s two-sided unpaired *t*-test (**a**,**b**,**d**,**e**) or two-way ANOVA (**f**,**h**). Source Data

To identify the GPCR target of TVA, we screened the six known fatty acid-binding GPCRs, including GPR40, GPR41, GPR43, GPR84, GPR119 and GPR120 (ref. ^23^) by siRNA-mediated knockdown (Extended Data Fig. 8d). Of these, only knockdown of GPR43 abolished TVA-enhanced TNF levels in primary mouse CD8^+^ T cells (Fig. 4a). Further analysis confirmed that knockdown of GPR43 in primary mouse CD8^+^ T cells leads to increased cAMP, p-CREB and p-STAT1 levels (Extended Data Fig. 8e) as well as increased TNF and IFNγ (Extended Data Fig. 8f) in mouse primary CD8^+^ T cells that cannot be further activated by TVA treatment. Similar results were obtained using OT-I T cells with siRNA-mediated GPR43 knockdown (Extended Data Fig. 8g), and OT-I T cells with knockout of *Gpr43* by CRISPR–Cas9 (Fig. 4b and Extended Data Fig. 8h). These data suggest that GPR43 has a suppressive role in CD8^+^ T cell activation, and that TVA is likely to attenuate GPR43 function. These results are also consistent with previous reports linking activation of GPR43 with Gα_i_ coupling and decreased cAMP levels^7,10^. Consistent with these findings, stimulation with anti-CD3/CD28 resulted in a much larger increase in GPR43 mRNA and protein expression levels in CD8^+^ T cells than that in CD4^+^ T cells (Extended Data Fig. 8i). This may partly explain why activated CD8^+^ T cells are sensitive to TVA treatment.

**Extended Data Fig. 9:**
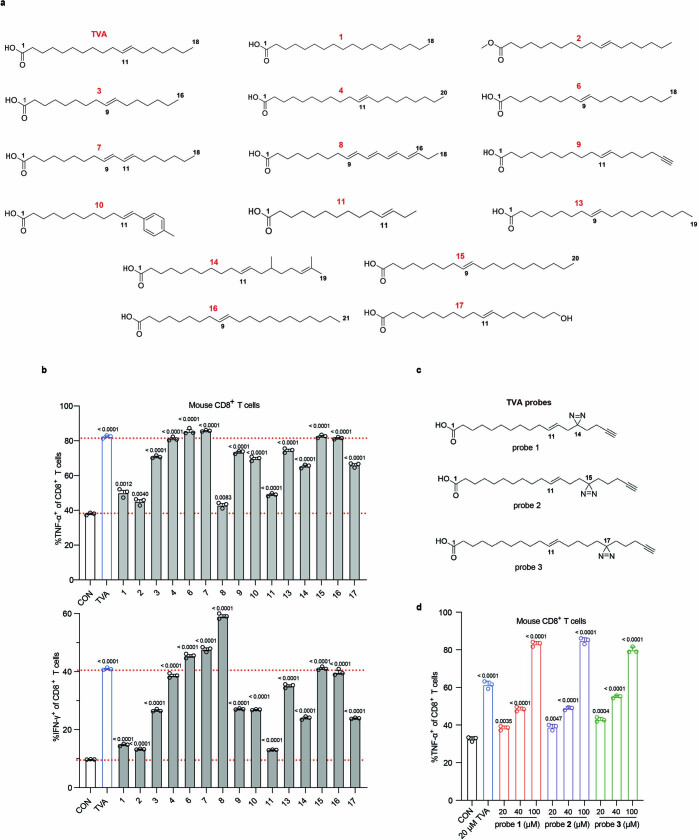
TVA derivatives and probes exhibit distinct bioactivity to enhance CD8^+^ T cell function. **a**, Chemical structures of TVA and 15 TVA derivatives. **b**, Effects of treatment with 20 µM TVA or TVA derivatives on TNF-α (*upper*) and IFN-γ (*lower*) levels in CD8^+^ T cells (*n* = 3). **c**, Chemical structures of the three synthetic designed photo-affinity probes of TVA. **d**, Effects of treatment with 20, 40, or 100 µM TVA probes on TNF-α level in mouse CD8^+^ T cells (*n* = 3). Data are mean ± SD (**b**, **d**). Student’s two-sided unpaired *t* test (**b**, **d**). Source Data

We next performed structure–activity relationship (SAR) studies on TVA. We designed 15 TVA derivatives (Extended Data Fig. 9a; synthetic procedures and NMR results of TVA derivatives and probes are presented in Supplementary Information) with changes in chain length, double bond position or number, tail group or terminal acid group, and compared them with TVA for bioactivity to enhance CD8^+^ T cell activation (Extended Data Fig. 9b). The results of the SAR studies revealed that the double bond (**1**), acid group (**2**) and a length of at least 16 carbons (**3**, **4**, **13**, **15** and **16**) were crucial for maintaining TVA bioactivity. In addition, shifting the position of the double bond from C11–C12 to C9–C10 (**6**), or adding a C9–C10 double bond (**7**) resulted in enhanced TVA bioactivity, suggesting that a C9–C10 double bond may be optimal for TVA bioactivity. The SAR results for **9**, **10**, **14** and **17** also suggest that the terminal chain can be modified, and the resulting TVA derivatives retained 70–80% bioactivity. We thus designed photo-affinity probes using TVA as a parental molecule to bear a photo-reactive diazirine group and a tail alkynyl group (Extended Data Fig. 9c). All three TVA probes enhanced CD8^+^ T cell activity in a dose-dependent manner (Extended Data Fig. 9d). We next performed a photo-affinity labelling study^24^ using mouse CD8^+^ T cells treated with TVA probe 3. The diazirine enables covalent photo-crosslinking to contacting protein residues and the alkynyl group enables linking the reporter (biotin) and probing TVA-binding protein target(s). Western blotting using biotin-labelled proteins pulled down by streptavidin beads demonstrated binding between the TVA probe and GPR43 (Fig. 4c).

**Extended Data Fig. 10:**
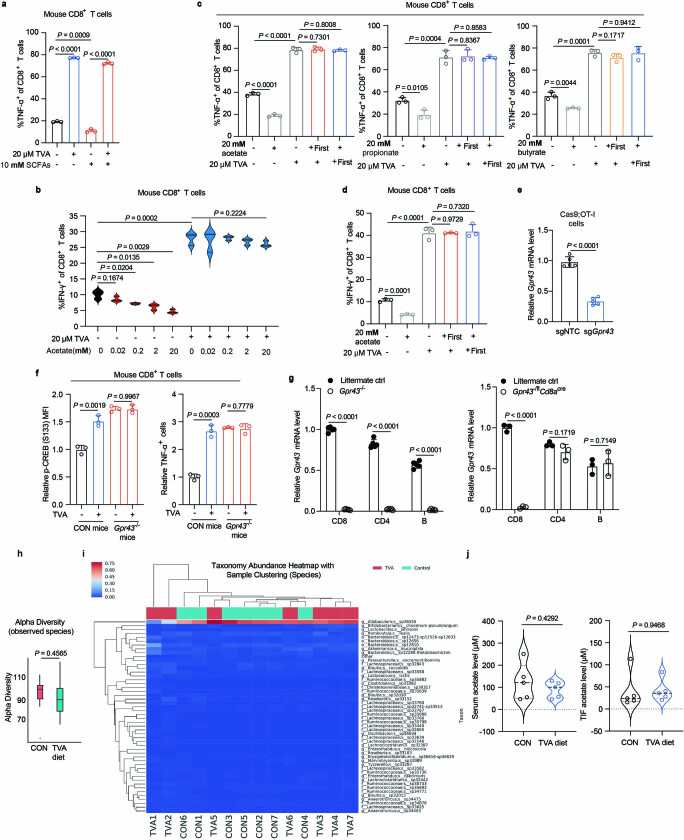
TVA inactivates GPR43 by overshadowing its SCFA agonists. **a**, Effect of treatment with 10 mM short chain fatty acids mix (mix of acetate, propionate, and butyrate) with or without 20 µM TVA on TNF-α expression in mouse CD8^+^ T cells (*n* = 3). **b**, Effects of synchronized treatment with different doses of acetate with or without 20 µM TVA on IFN-γ level in mouse CD8^+^ T cell (*n* = 3). **c**, Effects of sequential treatment with 20 mM acetate (*left*) or 20 mM propionate (*middle*) or 20 mM butyrate (*right*) and 20 µM TVA (acetate or propionate or butyrate first: acetate or propionate or butyrate for12 h first and then TVA added for another 12 h; TVA first: TVA for 12 h first and then acetate or propionate or butyrate added for another 12 h) on TNF-α level in CD8^+^ T cells (*n* = 3). **d**, Effects of subsequent treatment with 20 mM acetate and 20 µM TVA (acetate first: acetate for 12 h first and then TVA added for another 12 h; TVA first: TVA for 12 h first and then acetate added for another 12 h) on IFN-γ level in CD8^+^ T cells (*n* = 3). **e**, *Gpr43* knockout efficiency in Cas9;OT-I cells which were used for ACT experiment was checked by RT-PCR (*n* = 5). **f**, Effects of TVA treatment on p-CREB (*left*), and TNF-α level (*right*) in *Gpr43*^−/−^ or control mouse CD8^+^ T cells were shown (*n* = 3). **g**, Expression of *Gpr43* mRNA was examined in CD8^+^, CD4^+^ T cells, and B cells from *Gpr43*^−/−^ and littermate control mice (*left*) (*n* = 5) or from *Gpr43*^−/fl^*Cd8a*^cre^ and littermate control mice (*right*) (*n* = 3). **h**, TVA shows no significant effect on microbiota diversity. Box-and-whisker plot showing alpha diversity of gut feces microbiota in B16F10 tumor-bearing mice fed with control diet or TVA-enriched diet (*n* = 7), whiskers are min to max, central line is mean, bounds of box are mean ± SD. **i**, Heatmap showing the microbial composition of the samples at the species level with the top fifty most abundant species identified. Each row represents the abundance for each taxon, with the taxonomy ID shown on the right. Each column represents the abundance for each sample, with the sample ID shown at the bottom. Group information is indicated by the colored bar located on the top of each column. Hierarchical clustering was performed on samples based on Bray-Curtis dissimilarity. Hierarchical clustering was also performed on the taxa so that taxa with similar distributions are grouped together (*n* = 7 mice). **j**, Acetate levels in serum (*left*) and TIF (*right*) from B16F10 tumor-bearing mice were measured by ^1^H nuclear magnetic resonance (NMR) spectroscopy (*n* = 5). Data are mean ± SD (**a**-**h**, **j**). Student’s two-sided unpaired *t* test (**a**-**h**, **j**). Source Data

We next found that GPR43 SCFA agonists (at 20 mM) significantly decreased mouse CD8^+^ T cell activation, indicated by decreased TNF expression, whereas TVA at a 1,000× lower level (20 μM) was able to reverse SCFA-dependent suppression on CD8^+^ T cells (Extended Data Fig. 10a). In addition, increasing concentrations of SCFA acetate up to 20 mM attenuated CD8^+^ T cell activation, as assessed by reduced TNF (Fig. 4d) and IFNγ (Extended Data Fig. 10b) levels; this readout was effectively reversed by 20 μM TVA. By contrast, 2 μM TVA was sufficient to reverse the suppression of CD8^+^ T cells by acetate at 20 mM (Fig. 4e). Consistent with these findings, suppression of CD8^+^ T cells with reduced TNF (Extended Data Fig. 10c, left) and IFNγ (Extended Data Fig. 10d) levels by pre-treatment with 20 mM acetate could be reversed by 20 μM TVA. By contrast, enhanced CD8^+^ T cell activation by pre-treatment with 20 μM TVA could not be reversed with 20 mM acetate. Similar results were obtained using other SCFA agonists, including propionate and butyrate (Extended Data Fig. 10c, middle and right). These results suggest that TVA may bind to and inactivate GPR43 by antagonizing its SCFA agonists.

## GPR43 requirement for TVA activity

We first examined an adoptive cell therapy (ACT) mouse model using Cas9-expressing mouse OT-I T cells. Syngeneic mice inoculated with mouse B16F10 melanoma cells that express the cognate antigen (B16-OVA) were used as recipients for OT-I T cells to determine anti-tumour immunity (Fig. 4f, left). Adoptive transfer of OT-I cells with control single guide RNA (sgRNA) resulted in decreased tumour growth, which was further reduced with a TVA-enriched diet (Fig. 4f, right). By contrast, adoptive transfer of OT-I cells with CRISPR–Cas9-mediated *Gpr43* knockout (Extended Data Fig. 10e) led to markedly reduced tumour growth that could not be further attenuated by TVA diet (Fig. 4f, right). Consistent with these findings, adoptive transfer of OT-I cells with CRISPR–Cas9-mediated *Creb1* knockout led to attenuated anti-tumour effects in mice compared with control OT-I cells and did not respond to TVA diet (Fig. 4f, right).

We next used whole-body *Gpr43* knockout (*Gpr43*^−/−^) mice to perform syngeneic mouse model experiments with inoculated mouse B16F10 cells. Our in vitro study showed that primary mouse CD8^+^ T cells from *Gpr43*^−/−^ mice had increased p-CREB and TNF levels that were not further enhanced by TVA treatment (Extended Data Fig. 10f). Moreover, we found that GPR43 deficiency in *Gpr43*^*−/−*^ mice (confirmed in CD8^+^ and CD4^+^ T cells and in B cells by *Gpr43* mRNA levels; Extended Data Fig. 10g, left) abolished TVA diet-dependent reduction of the tumour growth observed in littermate control mice (Fig. 4g).

Finally, we used *Gpr43*^*−/fl*^*Cd8a*^*cre*^ mice with conditional *Gpr43* knockout in CD8^+^ T cells (confirmed in CD8^+^ T cells by *Gpr43* mRNA levels, with CD4^+^ T cells and B cells as controls; Extended Data Fig. 10g, right) to perform syngeneic mouse model experiments with mice inoculated with B16F10 cells. Conditional knockout of *Gpr43* in CD8^+^ T cells abolished TVA diet-dependent reduction of the tumour growth observed in littermate control mice. Together, these results demonstrated that TVA requires GPR43 in CD8^+^ T cells to enhance CD8^+^ T cell function and consequent anti-tumour immunity in vivo.

## TVA antagonizes effects of SCFAs on cAMP

**Extended Data Fig. 11:**
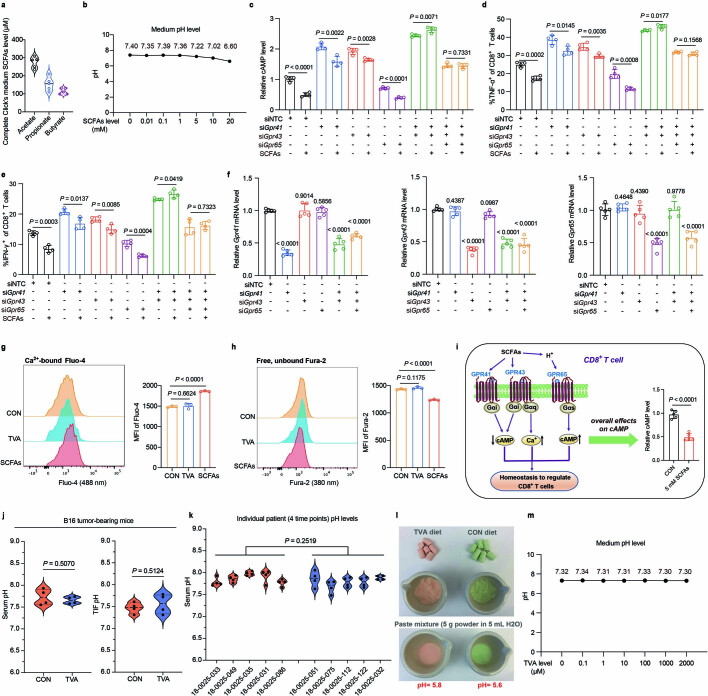
SCFAs affect mouse CD8^+^ T cell activity through GPR41, GPR43, and GPR65. **a**, SCFAs level in Click’s medium (n = 5). **b**, Medium pH level with different doses of SCFAs. **c**, Effects of *Gpr41*, *Gpr43*, and *Gpr65* transient knockdown on cAMP level in mouse CD8^+^ T cells treated with or without SCFAs (n = 4). **d**, Effect of *Gpr41*, *Gpr43*, and *Gpr65* transient knockdown on TNF-α level in mouse CD8^+^ T cells treated with or without SCFAs (n = 4). **e**, Effect of *Gpr41*, *Gpr43*, and *Gpr65* transient knockdown on IFN-γ level in mouse CD8^+^ T cells treated with or without SCFAs (n = 4). **f**, *Gpr41* (*left*), *Gpr43* (*middle*), and *Gpr65* (*right*) knockdown efficiency checked by RT-PCR (n = 5). **g**, Flow cytometry and quantification of Ca^2+^-bound Fluo-4 level (n = 3). **h**, Flow cytometry and quantification of free unbound Fura-2 level (n = 3). **i**, Working model of effect of SCFAs on cAMP level in CD8^+^ T cells (n = 5). The figure was generated using BioRender. **j**, pH level of B16F10 tumor-bearing mouse serum (*left*) and TIF (*right*) (n = 4). **k**, Serum pH level of 10 lymphoma patients that have undergone commercial CAR-T cell therapy. Each patient has blood collections at 4 different timepoints (detailed information can be found in Supplementary Table 8). **l**, pH levels of TVA and CON diet. **m**, Medium pH level with different doses of TVA. Data are mean ± SD (**a**, **c**-**k**). Student’s two-sided unpaired *t* test (**a**, **c**-**k**). Source Data

A TVA-enriched diet did not significantly alter the diversity and patterns of gut microbial distribution in mice (Extended Data Fig. 10h,i), or serum or TIF levels of gut microbiota-derived SCFA acetate (Extended Data Fig. 10j). The concentrations of acetate, propionate and butyrate in culture medium are in the range of 100–300 μM (Extended Data Fig. 11a), suggesting that SCFAs in the culture medium activate GPR43 in CD8^+^ T cells and predispose them to TVA-mediated inactivation. We thus compared the opposing effects of TVA and SCFAs on cAMP signalling in CD8^+^ T cells. We first examined whether SCFAs inhibit CD8^+^ T cell activity through GPR41 and GPR43, as well as the pH sensor GPR65 (ref. ^25^), since unlike long-chain fatty acids such as TVA, SCFAs are soluble in water and reduce the luminal pH. GPR65 is activated by low pH and signals through Gα_s_, increasing cAMP production and consequent CREB phosphorylation and activation^25^. Indeed, SCFAs in the culture medium reduced the pH in mouse primary CD8^+^ T cells (Extended Data Fig. 11b), accompanied by reduced cAMP, TNF and IFNγ expression (Extended Data Fig. 11c–e), whereas individual or combined knockdown of GPR41 or GPR43 (knockdown efficiency shown in Extended Data Fig. 11f) led to increased CD8^+^ T cell activity. This increase in CD8^+^ T cell activity could be partially decreased with additional SCFA treatment. By contrast, knockdown of GPR65 led to decreased CD8^+^ T cell activity, with additional SCFA treatment resulting in further reduction, whereas triple knockdown of GPR41, GPR43 and GPR65 completely abolished the SCFA-mediated effects on CD8^+^ T cells. Finally, SCFAs increased the intracellular Ca^2+^ level in CD8^+^ T cells, probably through Gα_q_ (Extended Data Fig. 11g,h). By contrast, TVA exhibited minimal effects in both assays. Together, SCFAs inhibit overall CD8^+^ T cell activity, consistent with our results showing that the negative regulation of cAMP mediated by GPR41 and GPR43 antagonizes the positive effects of GPR65 on cAMP levels (Extended Data Fig. 11i).

**Fig. 5:**
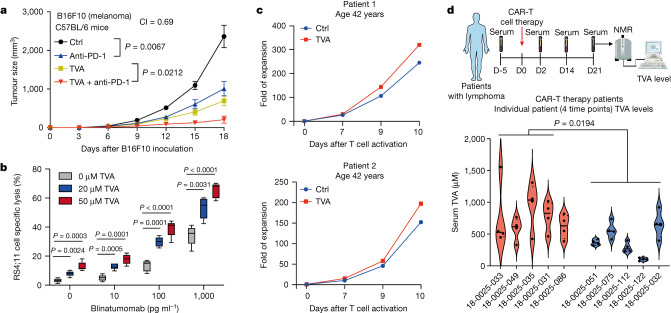
TVA augments the effectiveness of multiple T cell-based anti-cancer therapies. **a**, Effect of anti-PD-1 antibody on B16F10 tumour growth in C57BL/6 mice fed with TVA-enriched diet or control diet (*n* = 8). **b**, Box plots representing combined effect of blinatumomab and TVA at indicated concentrations on specific lysis of RS4;11 target cells in the presence of PBMCs by flow cytometry (*n* = 5). The central line is the mean, whiskers extend to minimum and maximum values and the box edges show mean ± s.d. **c**, Effects of treatment with 20 µM TVA on in vitro expansion of anti-CD19–CD28z CAR-T from patients with lymphoma. **d**, Top, schematic depicting experimental design for serum collection from patients with CAR-T cell therapy. This schematic was generated using BioRender.com. Bottom, violin plots showing serum TVA levels of 10 patients with lymphoma who have undergone commercial CAR-T cell therapy. Blood was collected from each patient at four different timepoints (detailed information can be found in Supplementary Table 8). Red violin plots represent the patients who have complete response to CAR-T cell therapy, and blue violin plots represent patients who have progressive disease under CAR-T cell therapy. Data are mean ± s.e.m (**a**) or mean ± s.d. (**b**,**d**). Two-way ANOVA (**a**) or Student’s two-sided unpaired *t-*test (**b**,**d**). Source Data

**Extended Data Fig. 12:**
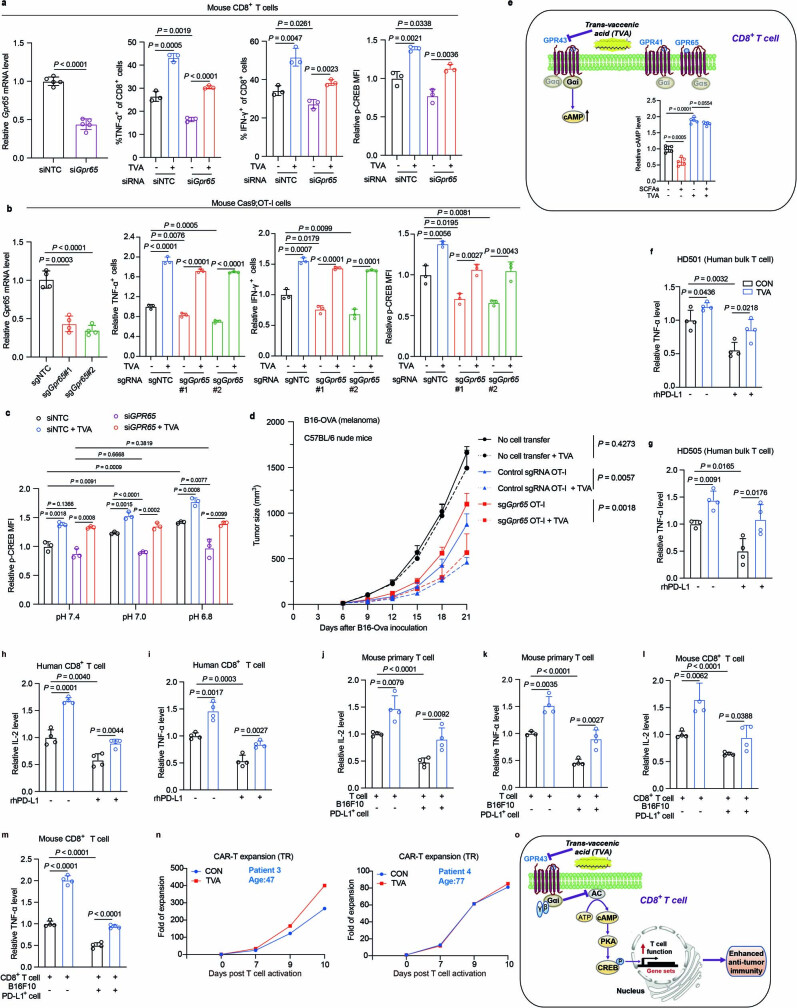
TVA-enhanced CD8^+^ T cell function independent of pH sensor GPR65. **a**, *Gpr65* knockdown efficiency in CD8^+^ T cells was checked by RT-PCR (*left*) (n = 5). Effect of *Gpr65* transient knockdown on TNF-α (*middle left*), IFN-γ (*middle right*), and p-CREB (*right*) levels (n = 3) in mouse CD8^+^ T cells treated with or without TVA were shown. **b**, Knockout efficiency of *Gpr65* in Cas9;OT-I cells was checked by RT-PCR (*left*) (n = 4). Effect of *Gpr43* knockout with two different sgRNAs on TNF-α (*middle left*), IFN-γ (*middle right*), and p-CREB (*right*) levels (n = 3) in mouse Cas9;OT-I cells treated with or without TVA were shown. **c**, Effects of *Gpr65* transient knockdown on p-CREB levels in mouse CD8^+^ T cells treated with or without TVA under different pH (n = 3). **d**, Cas9;OT-I cells (5 × 10^6^) transduced with non-targeting control sgRNA or sgRNA against *Gpr65* were transferred into mice at day 6 after engraftment of B16-OVA followed by analyses of tumor size. Non-treatment control mice received no transfer of T cells, control sgRNA OT-I and control sgRNA OT-I + TVA are the same as described in Fig. 4f because results shown in Fig. 4f and Extended Data Fig. 12d are from the same experiment (*n* = 8). **e**, Working model of effect of TVA on cAMP level in CD8^+^ T cells treated with SCFAs (n = 5). The figure was generated using BioRender. **f**, **g**, Effect of treatment with 20 µM TVA on human bulk T cells HD501 (**f**) and HD505 (**g**) exhaustion induced by purified PD-L1, assessed by IL-2 production levels (*n* = 4). **h**, **i**, Effect of treatment with 20 µM TVA on human CD8^+^ T cell exhaustion induced by purified PD-L1, assessed by IL-2 (**h**) and TNF-α (**i**) levels (*n* = 4). **j**, **k**, Effect of treatment with 20 µM TVA on mouse primary T cell exhaustion induced by co-cultured B16F10 cells expressing PD-L1 (B16F10 PD-L1^+^), assessed by IL-2 (**j**) and TNF-α (**k**) levels (*n* = 4). **l**, **m**, Effect of treatment with 20 µM TVA on mouse CD8^+^ T cells exhaustion induced by co-cultured B16F10 cells expressing PD-L1 (B16F10 PD-L1^+^), assessed by IL-2 (**l**) and TNF-α (**m**) levels (*n* = 4). **n**, Effect of treatment with 20 µM TVA on expansion of anti-CD19-CD28z CAR-T cells derived from lymphoma patients in vitro. **o**, Working model. The figure was generated using BioRender. Data are mean ± SD (**a**-**c**, **e**-**m**) or mean ± s.e.m (**d**). Student’s two-sided unpaired *t* test (**a**-**c**, **e**-**m**) or two-way ANOVA (**d**). Source Data

By contrast, we found that TVA diet does not alter the pH in serum and TIF samples from mice fed on control or TVA diets (Extended Data Fig. 11j), and no significant difference of pH was detected in human primary serum samples from a group of lymphoma patients with different serum levels of TVA (Extended Data Fig. 11k; detailed description in Fig. 5d). We also found that mixing TVA or control diets with water results in comparable pH values, and that TVA up to 2,000 μM does not significantly alter the pH values in culture medium (Extended Data Fig. 11l, m). Moreover, knockdown or CRISPR–Cas9-mediated knockout of *Gpr65* in primary mouse CD8^+^ T cells in vitro leads to decreased TNF, IFNγ and p-CREB levels but had no effects on TVA-enhanced CD8^+^ T cell function (Extended Data Fig. 12a,b), nor does changing pH of culture medium (Extended Data Fig. 12c). Lastly, in an ACT mouse model using Cas9-expressing OT-I T cells, CRISPR–Cas9-mediated *Gpr65* knockout resulted in decreased tumour growth, which, however, was further reduced by TVA diet (Extended Data Fig. 12d; note that top 4 control groups are from the same experiment described in Fig. 4f). These results demonstrate that TVA does not lower pH or signal through GPR65 to enhance CD8^+^ T cell function in vitro and in vivo, supporting our hypothesis that TVA inactivates GPR43 and antagonizes the overall negative effects of SCFAs on cAMP (Extended Data Fig. 12e).

## TVA augments T cell-based therapies

TVA treatment significantly reversed exhaustion of primary human bulk T cells and CD8^+^ T cells treated with purified recombinant PD-L1 (Extended Data Fig. 12f–i). Similar results were obtained using mouse primary T cells co-cultured with B16F10 cells overexpressing PD-L1 (Extended Data Fig. 12j–m). Moreover, dietary TVA combined with anti-PD-1 antibody, representative of immune checkpoint inhibitor therapy^26^, showed synergistic attenuation of B16F10 tumour growth (Fig. 5a). We next tested the effects of TVA on efficacy of blinatumomab, a bispecific T cell engager that targets CD19 on B cells and CD3 on T cells^27^. TVA significantly enhanced the in vitro killing efficiency of human peripheral blood mononuclear cells (PBMCs) on human B-ALL RS4;11 cells in the presence of blinatumomab in a dose-dependent manner (Fig. 5b). Furthermore, TVA increased in vitro expansion of chimeric antigen receptor (CAR) T cells derived from primary T cells from 3 patients with lymphoma aged between 42 and 47 years (Fig. 5c and Extended Data Fig. 12n, left). By contrast, cells from an older patient (age 77 years) did not respond to TVA treatment (Extended Data Fig. 12n, right), which might be owing to a lower starting cell number compared with the younger patients. Of note, in a retrospective clinical study, we found that serum TVA levels were higher in a group of patients with lymphoma that responded to CAR-T cell therapy than in non-responders (Fig. 5d; detailed timepoint information can be found in Supplementary Table 8). These findings align with the notion that dietary TVA may improve clinical responsiveness to T cell-based immunotherapies.

## Discussion

Our findings reveal a mechanism during human dietary evolution by which extraorganismal TVA reprograms CD8^+^ T cells through extrinsic regulation to inactivate GPR43, in contrast to gut microbiota-derived, intraorganismal SCFAs as GPR43 agonists (Extended Data Fig. 12o). Our study, by comprehensive evaluation of diet-derived nutrients, advances the identification and understanding of mechanistic links between diet and human physiology and pathology. Thus, despite the vast diversity of food and diet origins, studies that focus on individual nutrients can be used to identify the molecular mechanisms underlying nutritional influences on human health and disease, as well as effectiveness of therapies. The approach used in our study could have broad implications for future elucidation of previously unknown physiological and pathological roles of circulating nutrients in human health and disease.

As a natural food component, TVA has high translational potential as a dietary element in therapeutic approaches to improve clinical outcomes of diverse anti-cancer therapies such as immune checkpoint inhibitors, T cell engagers and CAR-T and T cell receptor T cell therapy. Epidemiological studies suggest that the circulating levels of TVA in humans are associated with lower adiposity, diabetes risk and systemic inflammation, but the effects of dietary TVA on the risk of cancer and cardiovascular diseases are unclear, although in mouse models of dyslipidaemia, a TVA-enriched diet exerts hypolipidemic effects by lowering circulating triglyceride levels^28–30^. Nevertheless, a comprehensive understanding of the interactive and collective influences of diverse dietary nutrients on cancer risk, development and therapy responses is crucial for diet choices. For example, consuming red meat may provide TVA for improved anti-tumour immunity, but a high intake of red meat has been positively associated with risk of many cancers, including breast, colorectal, colon and rectal cancer^31^. Thus, our studies support TVA supplementation as a more targeted and efficient way than dietary changes to benefit anti-tumour immunity.

The GPR43–CREB mechanism may be cell type-specific for CD8^+^ T cells. For example, although TVA treatment increased IL-2 production by CD4^+^ T cells, TVA did not alter the production of effector molecules including TNF and IFNγ, or proliferation/apoptosis of CD4^+^ T cells. Thus, the overall effects of TVA on CD4^+^ T cells are modest compared to those on CD8^+^ T cells, which may be related to the low expression of GPR43 in CD4^+^ T cells. Future studies are warranted to determine how GPR43 switches its downstream effector pathways in different immune cells, whether TVA regulates functions of other immune cells with high levels of GPR43 expression such as monocytes, neutrophils and marginal zone B cells^7,11,32,33^, and whether TVA similarly inactivates GPR43 for activation of the cAMP–PKA–CREB axis in these cells. Finally, since TVA is bulky compared with SCFAs, it is unlikely to compete with them for their binding pocket on GPR43. It may bind to a different site and function as a negative allosteric modulator. Further studies are warranted to elucidate the underlying structural and molecular mechanisms by which TVA inactivates GPR43.

## Methods

### Cell lines

All cell lines were authenticated by genomic short tandem repeat (STR) profiling at the University of Chicago Integrated Genomics Core (EIGC) upon purchase and at least annually as appropriate. Human T lymphocyte cell line Jurkat T was purchased from American Type Culture Collection (ATCC). Human Plat-E cells and mouse melanoma cancer cell line B16-OVA were provided by H.C. Human RS4;11 cells were provided by the laboratory of W.S. Mouse melanoma cancer cell line B16F10, breast cancer cell line E0771, and Lewis lung carcinoma cell line LLC1 were purchased from ATCC. Mouse colorectal adenocarcinoma cell line MC38 was purchased from Kerafast. Plat-E, B16-OVA, B16F10, E0771, and LLC1 cells were cultured in Dulbecco Modified Eagle Medium (DMEM) with 10% fetal bovine serum (FBS) (Sigma, F2442) and penicillin/streptomycin. Jurkat T cells were cultured in RPMI-1640 medium with 10% FBS and penicillin/streptomycin. All cells were cultured at 37 °C and 5% CO_2_. Cell line experiments were conducted and designed according to protocols approved by Institutional Biosafety Committee (IBC) of the University of Chicago.

### Primary cells

Cas9-expressing OT-I cells were isolated from the spleen and peripheral lymph nodes (provided by the laboratory of H.C.) of Cas9;OT-I mice^34^ by magnetic bead purification using EasySep Mouse naive CD8^+^ T Cell Isolation Kit according to the manufacturer’s instructions (Stem Cell Technologies). Cas9;OT-I cells were activated in vitro for 18 h with plate-bound anti-CD3 (10 μg ml^−1^; Biolegend) and anti-CD28 (5 μg ml^−1^; Biolegend) antibodies in Click’s medium at 37 °C and 5% CO_2_ incubator for further experiments.

### Mice

Animal experiments were conducted and designed according to protocols approved by the Institutional Animal Care and Use Committee of The University of Chicago. Mouse were housed and bred at the University of Chicago Animal Resource Center in specific pathogen-free conditions. Mice were on 12-hour light/dark cycles that coincide with daylight in Chicago, IL, USA. The housing facility was maintained at 20–25 °C and 30–70% humidity. C57BL/6 J (The Jackson Laboratory, JAX:000664; RRID:IMSR_JAX:000664), C57BL/6 nude (B6.Cg-Foxn1nu/J) (The Jackson Laboratory, JAX:000819; RRID:IMSR_JAX:000819), TCRα knockout (B6.129S2-Tcratm1Mom/J) (The Jackson Laboratory, JAX:002116; RRID:IMSR_JAX:002116), pmel-1 (B6.Cg-Thy1a/CyTg(TcraTcrb)8Rest/J) (The Jackson Laboratory, JAX:005023; RRID:IMSR_JAX:0050), OT-I (C57BL/6-Tg(TcraTcrb)1100Mjb/J) (The Jackson Laboratory, JAX:003831; RRID:IMSR_JAX:003831), *Cd8a-cre* (C57BL/6-Tg(Cd8a-cre)1Itan/J) (The Jackson Laboratory, JAX:008766; RRID:IMSR_JAX:008766) mice were purchased from The Jackson Laboratory. *Gpr43*^*−/−*^ and *Gpr43*^*fl/fl*^ mice were provided by B.L. Sex-and age-matched mice were used throughout the study at 7–12 weeks old, and both male and female mice were used. The genetically modified mice were viable and developed normally.

### Human samples

Serum from patients who had undergone commercial CAR-T cell therapy were from the University of Chicago cell therapy biobank. Supplementary Table 8 contains relevant patient information.

### Construction of PD-1^+^ Jurkat T cell line

Jurkat T cells were infected with pre-made lentivirus expressing human PD-1 (Gen Target, LVP1076-PBS) according to the manufacturer’s instructions. After infection for 24 h, cells were selected with 2 µg ml^−1^ puromycin to obtain PD-1^+^ Jurkat T cells. PD-1 expression level was checked using western blot.

### Circulating nutrient library screens

To construct circulating nutrient compound library for cell-based screening purposes, components such as antibodies that are difficult to distinguish due to a wide variety, and some supplements that only function at a whole organism level including fish oils and herbs were excluded. Physiological ranges of serum levels of individual nutrients are available in the human metabolome database (https://hmdb.ca/) and applied in the experimental design. For the initial screen (1a), Jurkat T cells were treated with nutrient library for 48 h and then activated with 2.5 μg ml^−1^ anti-CD3 and 0.5 μg ml^−1^ anti-CD28 antibodies for another 12 h, followed by measurement of IL-2 level in medium supernatant using an ELISA kit (Biolegend). For screen 1b, 1 × 10^5^ PD-1^+^ Jurkat T cells were co-cultured with 2 × 10^4^ H596 (PD-L1^+^) cells in a well of 96-well plate for 60 h, and then activated with 2.5 μg ml^−1^ anti-CD3 and 0.5 μg ml^−1^ anti-CD28 antibodies for another 12 h, followed by measurement of IL-2 level in medium supernatant using an ELISA kit. Please also refer to Supplementary Tables 1–4 for more details.

### Mouse tumour models

For C57BL/6 mice and the TCRα-knockout mice tumour model, mice were anaesthetized with isoflurane, shaved at the injection site, and then injected subcutaneously in the abdominal flank with 1 × 10^5^ B16F10, MC38 or LLC1 cells, or in the mammary gland with 2 × 10^5^ E0771 cells. C57BL/6 nude mice were injected subcutaneously in the abdominal flank with 1 × 10^5^ B16F10 cells. The tumour-bearing mice were assigned to TVA-enriched diet (1% TVA, special order from Research Diets), CVA-enriched diet (1% CVA, special order from Research Diets) or control diet (Research Diets) as of the day of tumour inoculation, with mice body weight monitored. Tumours were measured using a calliper every 2–3 days. Tumour volumes were calculated using the following formula: length × width × [(length × width) × 0.5] × π/6. Mice were euthanized at humane endpoints or day 11–15 for tissue collection.

For the 666-15 treatment mouse model, 6–8-week-old C57BL/6 mice were anaesthetized with isoflurane, shaved the injection site, and then injected subcutaneously in the abdominal flank with 1 × 10^5^ B16F10 cells for tumour development. The tumour-bearing mice were assigned to TVA-enriched diet or control diet as of the day of tumour inoculation. When the tumour volume reached approximately 100 mm^3^, the mice were treated with either vehicle or 666-15 at 20 mg kg^−1^. The 666-15 was dissolved in 1% *N*-methylpyrrolidone, 5% Tween-80 in H_2_O. The mice were treated once a day for 5 consecutive days per week for 2 weeks. Tumours were measured using a calliper every 2–3 days. Tumour volumes were calculated using the following formula: length × width × [(length × width) × 0.5] × π/6. Mice were euthanized at humane endpoints.

For the anti-PD-1 treatment mouse model, 6–8-week-old C57BL/6 mice were anaesthetized with isoflurane, shaved the injection site, and then injected subcutaneously in the abdominal flank with 1 × 10^5^ B16F10 cells for tumour development. The tumour-bearing mice were assigned to TVA-enriched diet or control diet as of the day of tumour inoculation. On day 3, 6, 9, 12 and 15, 250 µg anti-PD-1 (BioXCell) or IgG control (BioXCell) was injected intraperitoneally. Tumours were measured the tumours using a calliper every 2–3 days. Tumour volumes were calculated using the following formula: length × width × [(length × width)0.5] × π/6. Mice were euthanized at humane endpoints.

For the *Gpr43* and *Creb1*-knockout Cas9;OT-I cells adoptive transfer mouse model, 4 × 10^5^ B16-OVA cells were injected subcutaneously in the abdominal flank of 6–8-week-old C57BL/6 nude mice on day 0, and tumour were allowed to grow for 6 days. Target genes were knocked out in Cas9;OT-I cells, 5 × 10^6^ cells were transferred to tumour-bearing C57BL/6 nude mice on day 6 by tail vein injection. The tumour-bearing mice were assigned to TVA-enriched diet or control diet on day 6, tumours were measured using a calliper every 2–3 days. Tumour volumes were calculated using the following formula: length × width × [(length × width) × 0.5] × π/6. Mice were euthanized at humane endpoints.

For the *Gpr43*^−/−^ mouse tumour model, 6–8-week-old *Gpr43*^−/−^ or littermate control mice were anaesthetized with isoflurane, shaved at the injection site, and then injected subcutaneously in the abdominal flank with 1 × 10^5^ B16F10 cells for tumour development. The tumour-bearing mice were assigned to TVA-enriched diet or control diet as of the day of tumour inoculation, tumours were measured using a calliper every 2–3 days. Tumour volumes were calculated using the following formula: length × width × [(length × width) × 0.5] × π/6. Mice were euthanized at humane endpoints.

For the *Gpr43*^*−/fl*^*Cd8a*^*cre*^ mouse tumour model, *Gpr43*^−/−^, *Gpr43*^*fl/fl*^ and *Cd8a*^*cre*^ mice were used for breeding to get *Gpr43* *Cd8* conditional-knockout mice (*Gpr43*^*−/fl*^*Cd8a*^*cre*^). Six- to eight-week-old *Gpr43*^*−/fl*^*Cd8*^*cre*^ or littermate control mice were anaesthetized with isoflurane, shaved the injection site, and then injected subcutaneously in the abdominal flank with 1 × 10^5^ B16F10 cells for tumour development. The tumour-bearing mice were assigned to TVA-enriched diet or control diet as of the day of tumour inoculation, tumours were measured using a calliper every 2–3 days. Tumour volumes were calculated using the following formula: length × width × [(length × width) × 0.5] × π/6. Mice were euthanized at humane endpoints.

### Diet formula

Detailed information on the full compositions of the control, TVA- and CVA-enriched diets is summarized in Supplementary Table 9.

### Antibody-mediated T cell depletions

Five- to eight-week-old C57BL/6 mice were injected subcutaneously in the abdominal flank with 1 × 10^5^ B16F10 cells, and then injected intraperitoneally with six doses of depleting antibodies (anti-CD8α, BioXCell, clone 2.43) or isotype control (rat IgG2β isotype control, BioXCell, clone LTF-2) on days 1 (200 μg), 2 (200 μg), 4 (200 μg), 8 (200 μg), 12 (200 μg) and 16 (200 μg) relative to tumour injection (day 0). Cheek bleeds were collected and subjected to flow cytometry to check CD8^+^ T cell depletion efficiency on days 3, 12 and 18 using antibodies targeting non-competing CD8 epitopes (BV711 anti-mouse CD8α).

### Secreted cytokines level

Human or mouse T cell secreted IL-2, TNF and IFNγ were detected by ELISA MAX Standard Set (Biolegend) per the manufacturer’s instructions.

### Pmel-1 killing assay

Pmel-1 cells were isolated from a Pmel mouse and seeded at density of 1 × 10^6^ cells/well in 6-well plate (pre-coated with anti-CD3 and anti-CD28) for 18 h. One million activated Pmel-1 cells were then co-cultured with 2 × 10^5^ B16F10 cells for 24 h with or without 20 µM TVA. All cells in suspension were collected, stained with anti-mouse CD45 and PI, and analysed by flow cytometry.

### Cell proliferation assay

Cell proliferation assays were conducted by seeding 5 × 10^4^ cells in a 6-well plate. Cell numbers were recorded daily within 5 days using TC20 Automated Cell Counter (Bio-Rad).

### Extraction and quantification of TVA levels by NMR

TIF and serum from tumour-bearing animals were isolated as described^35^. In brief, tumours were dissected away from the euthanized animal, rinsed in a saline containing Petri dish, and then placed on top of a 20-μm mesh nylon filter (Spectrum Labs, 148134) that was affixed to the conical tube (Falcon, 1495949 A). After the conical tube lid was placed on top without screwing and taped using laboratory tape in place, tumour-containing conical tube was subjected to centrifugation at 4 °C for 10 min at 106*g* using a refrigerated clinical centrifuge. In total, 10–50 μl of fluid was obtained in the bottom of the conical tube. To prepare the mouse serum, once the tumours were dissected, blood was isolated from the mouse heart by cardiac puncture using a 1-ml 25 G TB syringe, allowed to clot by leaving it undisturbed at room temperature, and then centrifuged at 1,500*g* for 10 min in a refrigerated centrifuge. The resulting supernatant was designated serum. Human serum samples were obtained from the University of Chicago cell therapy biobank. The TIL and serum samples are lyophilized and subjected to quantification of TVA levels by ^1^H-NMR spectroscopy using a Bruker Ascend TM 600 spectrometer equipped with CryoProbeProdigy. In a typical procedure, 350 µl deuterated methanol (methanol-d4) with 0.03% tetramethylsilane (Oakwood Chemical) was added to a lyophilized sample to dissolve TVA. After being vortexed, the sample was then centrifuged at 10,000*g* for 5 min to collect the supernatant. ^1^H-NMR spectrum of the supernatant (250 µl) in a 335-pp Precision 3 mm NMR tube (Wilmad-Lab Glass) was acquired with delay time (d1) set to 2 s for 3,072 scans. TVA concentration was calculated based on the integral of peak at 1.97 ppm and the tetramethylsilane internal standard.

### CD45^+^ tumour-infiltrating leukocyte isolation

Tumour tissue were dissected from euthanized tumour-bearing mice, minced into small pieces ( ≤ 2 mm) using a scalpel in a dish, and then transferred to a 14-ml round-bottom tube containing 5 ml tumour digestion medium (500 μl collagenase/hyaluronidase solution, 750 μl 1 mg ml^−1^ DNase I solution, and 3.75 ml RPMI-1640 medium). After incubation at 37 °C for 25 min on a shaking platform, the digested tumour tissues were transferred into a 70-μm mesh nylon strainer on a 50 ml conical tube, pushed through the strainer using the rubber end of a syringe plunger, and rinsed with the recommended medium. After centrifugation at 300*g* for 10 min at room temperature with the brake on low, the resulting cell pellets were added 10 ml of ammonium chloride solution for incubation at room temperature for 5 min, followed by centrifugation at 300*g* for 10 min at room temperature with the brake on low. The resulting cell pellets were re-suspended at 1–10 × 10^6^ cells per ml in PBS and then subjected to CD45^+^ tumour-infiltrating leukocytes isolation by magnetic bead purification using EasySep Mouse TIL (CD45) Positive Selection Kit according to the manufacturer’s instructions (Stemcell Technologies).

### Mouse TIL isolation

Freshly excised mouse tumour tissues were minced into small pieces (volume smaller than 1 mm^3^) by scissors in PBS, digested by Collagenase IV (1 mg ml^−1^) and DNase I (200 U ml^−1^) at 37 °C for 20 min in PBS with gentle rocking. The digested tumour tissues were added 5 times volume 0.01 M EDTA, filtered into a new tube through 200 mesh screen (100 μm strainer), and then centrifuged at 300*g* for 5 min at room temperature. The resulting cell pellets were re-suspended with 3 ml PBS, laid gently on 3 ml lymphocytes isolation liquid (pre-warmed to room temperature), and subjected to density gradient centrifugation (300*g*, 30 min, room temperature, accelerate 6, decelerate 2). The middle layer of cells was moved carefully to a new tube, added PBS to 15 ml, and centrifuged (300*g*, 10 min, room temperature). The cell pellets (lyse red cells if necessary) were designated TILs and used for the following experiments.

### Mouse spleen lymphocyte isolation

Mouse spleens were disrupted with syringe plunger in 1 ml PBS in a 40-μm strainer filtered to a 15-ml tube, washed with PBS and centrifuged at 300*g* for 5 min. The resulting cell pellets were re-suspended the with 2 ml red cell lysis buffer (Invitrogen), incubated at room temperature for 10 min, and centrifuged at 300*g* for 5 min after adding 13 ml PBS. The resulting cell pellets were designated splenocytes and used for following experiments.

### Mouse dLNs lymphocyte isolation

dLNs were disrupted with syringe plunger in 1 ml PBS in a 40 μm strainer filtered to a 15-ml tube, washed with PBS, and centrifuged at 300*g* for 5 min. The resulting cell pellets were designated LN lymphocytes and used for following experiments.

### Primary CD8^+^ or CD4^+^ T cell isolation and activation

Mouse primary CD8^+^ or CD4^+^ T cells were isolated from the spleen and peripheral lymph nodes of C57BL/6 mice by magnetic bead purification using EasySep Mouse CD8^+^ or CD4^+^ T Cell Isolation Kit according to the manufacturer’s instructions (Stemcell Technologies). Human primary CD8^+^ T cells were isolated from PBMC (Zen-Bio) by human CD8^+^ T Cell Isolation Kit according to the manufacturer’s instructions (Stemcell Technologies). Isolated primary CD8^+^ or CD4^+^ T cells were activated in vitro for 18 h with plate-bound anti-CD3 (10 μg ml^−1^; Biolegend) and anti-CD28 (5 μg ml^−1^; Biolegend) antibodies in Click’s medium at 37 °C and 5% CO_2_ incubator. Activated CD8^+^ or CD4^+^ T cells were ready for further experiments. A naive CD8^+^ T cells control was maintained in Click’s medium containing 10 ng ml^−1^ IL-7 (BioLegend).

### Flow cytometry

Mouse primary cells isolated from tumour, spleen and dLNs were stained with fluorescent antibodies and analysed by flow cytometry.

For experiments with live/dead criteria, cells were first stained with Fixable Viability Dyes (FVD) (Thermo Fisher Scientific) according to the manufacturer’s instructions. Subsequent surface marker staining was performed in Flow Cytometry Staining Buffer (Thermo Fisher Scientific). Intracellular staining for flow panels containing nuclear proteins was performed using the eBioscience FoxP3/Transcription Factor Staining Buffer Set (Thermo Fisher Scientific) according to the manufacturer’s instructions. For intracellular staining of cytoplasmic proteins, the Fixation/Permeabilization Solution Kit (BD Biosciences) was used according to the manufacturer’s instructions.

For phospho-antibody staining, cells were incubated with FVD (cell viability dye) for 15 min at room temperature in a tube, re-suspended with 200 μl pre-warmed 1× BD Phosflow Lyse/Fix buffer directly into the tube, and incubated at 37 °C for 10–15 min, followed by centrifugation at 300*g* for 5 min. The resulting cell pellets were washed once with FACS buffer, permeabilized with 200 μl of BD Phosflow Perm Buffer III for 45 min on ice, and centrifuged at 300*g* for 5 min. The cell pellets were washed again with FACS buffer, centrifuged at 300*g* for 5 min, and incubated with antibodies in FACS buffer for 45 min-1hour at room temperature.

Mouse anti-CD11b antibody was used for myeloid cell (CD11b^+^) marker. After gated with CD11b^+^ cells, anti-F4/80 and Gr1 antibodies were used for macrophage (Gr1^−^F4/80^+^) markers, anti-CD11c antibody was used for dendritic cells (CD11c^+^) marker, anti-CD16 and CD63 antibodies were used for neutrophils (CD16^−^CD63^+^) markers, anti-CD14 antibody was used for monocytes (CD14^+^) marker.

Data were collected on BD LSR-Fortessa 4–15 flow cytometer or Attune NxT 4–14 and analysed using FlowJo v10.4.

### Antibodies

Rat anti-IgG2b isotype (BioXCell, BE0090; clone LTF-2; RRID:AB_1107780); mouse anti-CD8α (BioXCell, BE0061; clone 2.43; RRID:AB_1125541); mouse anti-PD-1 (BioXCell, BE0146; clone RMP1-14; RRID:AB_10949053); mouse PerCP/Cyanine5.5 anti-Ki-67 Antibody (Biolegend, 652423; clone 16A8; RRID:AB_2629530, 1:200); Brilliant Violet 605 anti-T-bet Antibody (Biolegend, 644817; clone 4B10; RRID:AB_11219388, 1:200); mouse APC anti-CD223 (LAG-3) Antibody (Biolegend, 125209; clone C9B7W; RRID:AB_10639935, 1:200); mouse Brilliant Violet 650 anti-CD223 (LAG-3) Antibody (Biolegend, 125227; clone C9B7W; RRID: AB_2687209, 1:200); mouse PerCP/Cyanine5.5 anti-CD366 Antibody (Biolegend, 134012; clone B8.2C12; RRID:AB_2632736, 1:200); PE anti-TCF1 (TCF7) Antibody (Biolegend, 655207; clone 7F11A10; RRID:AB_2728491, 1:200); human/mouse/rat FITC anti-CD278 (ICOS) Antibody (Biolegend, 313505; clone C398.4 A; RRID:AB_416329, 1:200); human/mouse FITC anti-GZMB Recombinant Antibody (Biolegend, 372205; clone QA16A02; RRID:AB_2687029, 1:200); mouse PE/Cyanine5 anti-CD69 Antibody (Biolegend, 104509; clone H1.2F3; RRID:AB_313112, 1:200); FITC anti-mouse CD63 Antibody (Biolegend, 143919; clone NVG-2; RRID:AB_2876488, 1:200); mouse APC anti-CD152 Antibody (Biolegend, 106309; clone UC10-4B9; RRID:AB_2230158, 1:200); mouse APC anti-CD279 (PD-1) Antibody (Biolegend, 135209; clone 29 F.1A12; RRID:AB_2251944, 1:200); mouse PE/Cyanine5 anti-CD4 Antibody (Biolegend, 100409; clone GK1.5; RRID:AB_312694, 1:200); mouse Brilliant Violet 421 anti-IL-2 Antibody (Biolegend, 503825; clone JES6-5H4; RRID:AB_10895901, 1:200); mouse APC anti-CD45.2 Antibody (Biolegend, 109813; clone 104; RRID:AB_389210, 1:200); mouse APC anti-IFNγ Antibody (Biolegend, 505810; clone XMG1.2; RRID:AB_315404, 1:200); human/mouse PE/Cyanine7 anti-Granzyme B Recombinant Antibody (Biolegend, 372213; clone QA16A02; RRID:AB_2728380, 1:200); mouse PerCP/Cyanine5.5 anti-TNF Antibody (Biolegend, 506321; clone MP6-XT22; RRID:AB_961435, 1:200); mouse Brilliant Violet 711 anti-CD8a Antibody (Biolegend, 100747; clone 53-6.7; RRID:AB_11219594, 1:200); mouse Brilliant Violet 421 anti-FOXP3 Antibody (Biolegend, 126419; clone MF-14; RRID:AB_2565933, 1:200); mouse APC anti-CD3 Antibody (Biolegend, 100235; clone 17A2; RRID:AB_2561455, 1:200); FITC anti-BCL-2 (Biolegend, 633503; clone BCL/10C4; RRID:AB_2028392, 1:200); mouse APC anti-CD98 (4F2) (Biolegend, 128211; clone RL388; RRID:AB_2750544, 1:200); mouse FITC anti-F4/80 Recombinant Antibody (Biolegend, 157309; clone QA17A29; RRID:AB_2876535, 1:200); mouse APC anti-Ly-6G (Gr1) Antibody (Biolegend, 127613; clone 1A8; RRID:AB_1877163, 1:200); mouse/human APC anti-CD11b Antibody (Biolegend, 101211; clone M1/70; RRID: AB_312794, 1:200); mouse PerCP anti-CD11c Antibody (Biolegend, 117325; clone N418; RRID: AB_893236, 1:200); Alexa Fluor 647 anti-mouse CD16 Antibody (Biolegend, 158021; clone S17014E; RRID: AB_2904300, 1:200); PE/Cyanine5 anti-mouse CD28 Antibody (Biolegend, 102108; clone 37.51; RRID: AB_312873, 1:200); mouse PE/Cyanine7 anti-CD14 Antibody (Biolegend, 123315; clone Sa14-2; RRID:AB_10641133, 1:200); mouse/human PE anti-Ki-67 Antibody (Biolegend, 151210; clone 11F6; RRID:AB_2716008, 1:200); PE anti-LCK Phospho (Tyr394) (Biolegend, 933103; clone A18002D; RRID:AB_2820203, 1:200); PE TOX Monoclonal Antibody (TXRX10) (Thermo Fisher Scientific, 12-6502-82; clone TXRX10; RRID:AB_10855034, 1:200); APC Phospho-CREB (Ser133) Recombinant Rabbit Monoclonal Antibody (Thermo Fisher Scientific, MA5-36992; clone CREBS133-4D11; RRID:AB_2896927, 1:200); rabbit PE Active Caspase-3 (Thermo Fisher Scientific, BDB561011; clone C92-605; RRID:AB_2033931, 1:200); rabbit PE Phospho-Stat1 (Tyr701) Recombinant Monoclonal Antibody (Thermo Fisher Scientific, MA5-37039; clone Stat1Y701-3E6; RRID:AB_2896974, 1:200); GPR43 Polyclonal Antibody (Thermo Fisher Scientific, PA5-111780; clone N/A; RRID:AB_2857189, 1:500); Biotin Monoclonal Antibody (Z021) (Thermo Fisher Scientific, 03-3700; clone Z021; RRID:AB_2532265, 1:1,000); PKA C-α Antibody (Cell Signaling Technology, 4782 S; clone N/A; RRID:AB_2170170, 1:1,000); rabbit Stat1 (D1K9Y) monoclonal antibody (Cell Signaling Technology, 14994 S; clone D1K9Y; RRID:AB_2737027, 1:1,000); mouse monoclonal anti-β-actin antibody (Sigma-Aldrich, A1978; clone AC-15; RRID:AB_476692, 1:1,000); goat anti-mouse IgG (H + L) Secondary Antibody,HRP (Thermo Fisher Scientific, 31430; clone N/A; RRID:AB_228307, 1:1,000); goat anti-rabbit I clone gG (H + L) Secondary Antibody, HRP (Thermo Fisher Scientific, 31460; clone N/A; RRID:AB_228341, 1:1,000); goat Polyclonal IFNα/β R1 Antibody (Novus, AF3039-SP; clone N/A; RRID:AB_664107); hamster Monoclonal TNF RI/TNFRSF1A Antibody (Novus, MAB430-SP; clone 55R170; RRID:AB_2208782).

### Microbiome 16S sequencing

Gut faeces of control and TVA group (7 samples per group) B16F10 tumour-bearing mice were collected at day 16, and then subjected to microbiome 16S sequencing by Zymo Research. In brief, The ZymoBIOMICS-96 MagBead DNA Kit (Zymo Research) was used to extract DNA using an automated platform. Bacterial 16S ribosomal RNA gene targeted sequencing was performed using the Quick-16S NGS Library Prep Kit (Zymo Research). The bacterial 16S primers amplified the V3-V4 region of the 16S rRNA gene. The sequencing library was prepared using an innovative library preparation process in which PCR reactions were performed in real-time PCR machines to control cycles and therefore limit PCR chimera formation. The final PCR products were quantified with quantitative PCR fluorescence readings and pooled together based on equal molarity. The final pooled library was cleaned with the Select-a-Size DNA Clean & Concentrator (Zymo Research), then quantified with TapeStation (Agilent Technologies) and Qubit (Thermo Fisher Scientific). The ZymoBIOMICS Microbial Community Standard (Zymo Research) was used as a positive control for each DNA extraction, if performed. The final library was sequenced on Illumina MiSeq with a v3 reagent kit (600 cycles). The sequencing was performed with 10% PhiX spike-in. For Bioinformatics Analysis, unique amplicon sequences variants were inferred from raw reads using the DADA2 pipeline^36^. Potential sequencing errors and chimeric sequences were also removed with the Dada2 pipeline. Chimeric sequences were also removed with the DADA2 pipeline. Taxonomy assignment was performed using Uclust from Qiime v.1.9.1 with the Zymo Research Database, a 16S database that is internally designed and curated, as reference. Composition visualization, alpha-diversity, and beta-diversity analyses were performed with Qiime v.1.9.1 (ref. ^37^).

### [^13^C_1_]TVA metabolic flux analysis by gas chromatography–mass spectrometry

One million activated mouse primary CD8^+^ T cells were cultured for 24 h in RPMI-1640 medium containing 0, 20, 50 μM [^13^C_1_]TVA (Sigma), rinsed with 0.9% saline solution, and lysed with lysis buffer (400 µl of cold methanol, 300 µl of 0.88% (w/v) KCl). Lysed cells were scraped off the plate into a glass vial, 800 µl of cold dichloromethane was added and vortexed for 15 min at 4 °C, followed by centrifugation at maximum speed for 10 min at 4 °C. Samples were kept at room temperature after centrifugation to form two distinct phases. In total, 650 µl of the bottom dichloromethane fraction was transferred into a new glass tube and dried with nitrogen gas to obtain a lipid fraction pellet. FAME Derivatization was performed as previously described^38^. In brief, the dried lipid pellet was dissolved in 100 µl of toluene, added 200 µl of 2% sulfuric acid in methanol for incubation at 50 °C overnight and then added 500 µl of 5% NaCl to extract twice with 500 µl hexane. FAME cleanup was applied if analysing animal tissues (FAME Cleanup: a Florisil column was pre-conditioned with 3 ml of hexane, added methyl ester, and eluted twice with 1 ml isohexane-diethyl ether (95:5 v/v), with drying down in between elutions). Otherwise, samples were dried down under nitrogen and re-suspended in 50 µl of hexane to load onto gas chromatography–mass spectrometry (GC–MS). Derivatized samples were injected into a GC–MS using DB-FastFAME column (Agilent Technologies, G3903-63011) installed in an Agilent GC system. TVA-FAME has retention time of 9.6 min and *m*/*z* of 264 and 292, 13C1-TVA-FAME has retention time of 9.6 min and *m*/*z* 265. Metabolite levels and mass isotopomer distributions of derivatized fragments were analysed with an in-house MATLAB script, which integrated the metabolite fragment ions and corrected for natural isotope abundances.

### Cell culture treatment

Mouse primary CD8^+^ T cells were isolated, activated, and subjected to further treatment. Treatment with SSO was performed by incubating cells with 100 μM SSO for 24 h (ref. ^39^). For inhibitor and modulator treatments, SCH-202676 (200 nM), 666-15 (100 nM), H-89 dihydrochloride (200 nM), rhosin HCl (10 μM), NFAT inhibitor (1 μM), U0126 (100 nM), ruxolitinib (100 nM), SCFA mix (10 mM), acetate (0.02, 0.2, 2, 20 mM), butyrate (0.02, 0.2, 2, 20 mM) or propionate (0.02, 0.2, 2, 20 mM) were added to medium synchronized with 20 μM TVA^40–44^ for 24 h. For TVA washing experiment, mouse CD8^+^ T cells were treated with TVA for 24 h, washed off, and then cultured for another 24 h in medium with or without TVA. For 8-Bromo-cAMP and TVA different doses treatment experiment, activated mouse CD8^+^ T cells were treated with 8-Bromo-cAMP (0.01, 0.1, 1, 10, 100 μM) or TVA (10, 20, 100, 500, 1,000 μM) for 24 h, cells were collected for p-CREB level detection. For human T cell and CD8^+^ T cell rhPD-L1 assay, activated cells were treated with 0.5 μg ml^−1^ recombinant human PD-L1 (R&D) for 72 h in the presence or absence of 20 μM TVA, followed by ELISA detection of IL-2 and TNF level.

### KAS-seq and data analysis

Mouse and human CD8^+^ T cells were isolated, activated, and treated with or without 20 μM TVA for 20 min, 40 min, and 2 h. 500 mM N3-kethoxal was then supplemented into the medium followed by brief, vigorous shaking to fully homogenize the solution. The 6-well plates were then moved into the cell incubator (37 °C, 5% CO_2_) for 10 min to promote labelling of genomic ssDNA. Cell suspensions were then transferred to 15 ml conical tubes and centrifuged at 300*g* for 5 min at 4 °C. The supernatant medium was discarded, and genomic DNA was then extracted. The ssDNA with N3-kethoxal label was biotinylated, enriched, and fragmented following the established KAS-seq protocol^15,45^. Dual index libraries were constructed for high throughput sequencing using an Accel-NGS Methyl-seq DNA library kit and then sequenced at the Genomics Facility (University of Chicago) via Illumina NovaSeq 6000 (SP flowcell, 100 bp cassette) in two separate runs. Raw sequencing data under each condition was then catenated from the two runs, and adapter trimming; read deduplication and mapping; and differential KAS-seq analysis (comparing TVA-treated to untreated cells) was performed following the KAS-pipe analysis pipeline^15,45^. Volcano plots were subsequently generated in RStudio. For GO enrichment analysis, a list of gene bodies exhibiting differential ssDNA levels following TVA treatment was generated for each timepoint. *M. musculus* and *H. sapiens* CD8^+^ T cell KAS-seq data were assessed separately. The list of differentially expressed gene bodies was then submitted for GO enrichment and visualization, which was performed via the Gene Ontology project^18,19^ and REVIGO^46^ and Cytoscape^47^, respectively.

### Phospho-antibody array

To analyse signaling pathways, mouse primary CD8^+^ T cells were isolated, activated, and treated with TVA for indicated time, followed by Phospho Antibody Array (R&D Systems) according to the manufacturer’s instructions. The quantification for pixel density in each spot of the array was carried out by subtracting background signals from the spot intensity using software ImageJ (ImageJ, RRID: SCR_003070).

### Quantitative real-time PCR

Total RNA was extracted with TRIzol Reagent (Invitrogen) and then used for synthesizing the first strand cDNA with PrimeScript 1st strand cDNA Synthesis Kit (Takara) according to the manufacturer’s instructions. Quantitative RT–PCR was conducted with iTaq Universal SYBR Green Supermix (Bio-Rad).

### RNA-mediated interference with Accell siRNA

Mouse primary CD8^+^ T cells were isolated and cultured in replete medium (RPMI-1640 medium or Click’s medium containing 15% FBS, 55 µM 2-mercaptoethanol, 2 mM glutamine, penicillin/streptomycin, and either PHA, CD3 or IL-2) for 24 h, followed by incubation with Accell delivery mix (Accell siRNA Delivery Media (Horizon Discovery) with 1 μM siRNA, 20 IU ml^−1^ IL-2 and 1% FBS) for 72 h. Cells were collected for subsequent function analysis as well as depletion efficiency validation using RT–PCR.

### RNA sequencing

To check the effect of TVA treatment on gene expression of mouse CD8^+^ T cells, primary mouse CD8^+^ T cells were isolated, activated, and then treated with or without 20 μM TVA (TVA group versus control group) for 24 h, followed by RNA extraction using the PureLink RNA Mini Kit as the manufacturer’s instructions. RNA samples in triplicate were used for Illumina Next Generation Sequencing. To check effect of *Creb1* knockdown on TVA-dependent CD8^+^ T cell gene expression changes, mouse primary CD8^+^ T cells were isolated, activated, and then transfected with siNTC or si*Creb1* using Accell siRNA method, followed by treatment with or without 20 μM TVA for 24 h. RNA samples from four groups (siNTC, siNTC+TVA, si*Creb1*, si*Creb1* + TVA) were extracted and used for Illumina Next Generation Sequencing. Data processing and analysis were performed as previous described^48^.

### cAMP level

Mouse primary CD8^+^ T cells were isolated, activated, and then treated with 20 μM TVA for 0, 20 min, 40 min, and 2 h. cAMP-Screen Cyclic AMP Immunoassay System (Fisher Scientific Company) was used to check cAMP level according to the manufacturer’s instructions.

### [^13^C]Fatty acid tracing in vitro

Activated mouse primary CD8^+^ T cells were cultured for 6 or 24 h in Click’s medium with or without glucose, containing either 20 μM ^13^C_1_-TVA or 20 μM _13_C_1_-palmitate acid. The cells were then rinsed with 0.9% saline solution and lysed using lysis buffer (500 μl of 80% cold methanol). Samples were either freeze/thawed in N_2_/Ice or sonicated. Following this, 400 μl of dichloromethane was added and vortexed for 60 s. The mixture was then set on ice for 10 min to initiate partition, followed by centrifugation at 2,000*g* for 15 min at 4 °C. The polar (top) layer was carefully removed into a separate labelled tube and frozen, while the organic layer was dried down using N_2_ stream. After drying, 40 μl of methoxyamine (10 mg ml^−1^) in pyridine was added to each tube of dried, extracted metabolites. The solids were then re-suspended with sonication/vortexing and centrifuged briefly to remove insoluble debris. The pyridine solution was then transferred to autoinjector vials containing an insert and cooked at 70 °C for 10–15 min. Subsequently, 80 μl of TBDMS was added, briefly vortexed, and then cooked for 1 h at 70 °C. Finally, the samples were loaded for injection onto the GC–MS.

### Seahorse fatty acid oxidation assay

Mouse primary CD8^+^ T cells were isolated, activated, and then treated with 20 μM TVA or palmitic acid. The impact of TVA or palmitic acid treatment on FAO via assessing changes in OCR was checked by Seahorse XF Long Chain Fatty Acid Oxidation Stress Test Kit (Agilent Seahorse XF Sub OX) according to the manufacturer’s instructions.

### Ca^2+^ level of CD8^+^ T cells

Mouse primary CD8^+^ T cells were isolated, activated, and then treated with TVA or SCFAs for 24 h. Bound and unbound Ca2+ levels were measured by Fluo-4 kit (Thermo Fisher Scientific) and Fura-2 kit (Thermo Fisher Scientific) according to the manufacturer’s instructions.

### CRISPR editing of mouse OT-I cells

CRISPR editing of mouse primary CD8^+^ T cells was performed as previously described^34^. In brief, LMPd-sgNTC-mPGK-Ametrine, LMPd-sgGPR43#1-mPGK-Ametrine, LMPd-sgGPR43#2-mPGK-Ametrine, and LMPd-sgGPR43#3-mPGK-Ametrine were generated and co-transfected with pCL-Eco (Addgene, 12371) into Plat-E cells using TransIT-LT1 transfection reagent to produce retrovirus. Virus harvest medium was changed 18 h after transfection and then collected 48 h later. Primary Cas9-expressing OT-I (Cas9;OT-I) cells were derived from the spleen and peripheral lymph nodes of Cas9;OT-I mice by magnetic bead purification using EasySep Mouse T Cell Isolation Kit according to the manufacturer’s instructions (Stemcell Technologies). In total, 2–5 million Cas9;OT-I cells were activated, placed into one well of 24-well plate, and supplemented with retrovirus supernatant containing 10 μg ml^−1^ polybrene, followed by spin infection (800*g*, brake 3) for 3 h. After infection, cells were moved to 37 °C cell culture incubator for another 2 h and then changed with new complete Click’s medium (containing 20 IU ml^−1^ human IL-2, 2.5 ng ml^−1^ mouse IL-7, 25 ng ml^−1^ mouse IL-15) for 72 h. Cells were harvested, sorted with flow cytometry to enrich virus-transduced cells (Ametrine-positive), and subjected to subsequent function analysis as well as knockout efficiency validation using reverse transcription with PCR.

### Pull-down assay to identify the crosslinked protein-TVA complexes

Mouse primary CD8^+^ T cells were isolated and activated. 10 million cells were collected, pelleted, and re-suspended in 0.3 ml ice-cold PBS containing EDTA-free protease and phosphatase inhibitor cocktail (Thermo Fisher Scientific, A32961), followed by sonication on ice. Control probe N-1 or TVA Probe 3 was added and incubated at 37 °C for 2 h under dark conditions. After probe labelling, the sample was irradiated under 365 nm UV light for 6 min on ice, diluted with 1% SDS and sonicated on ice. The proteome concentration was determined using Pierce BCA Protein Assay Kit (Thermo Fisher Scientific) on a microplate reader (Bio-Rad) and normalized to 1.5 mg ml^−1^. 700 µl protein sample was conjugated with a biotin tag by click chemistry (100 μM biotin-azide, 100 μM TBTA, 1 mM CuSO_4_ and 1 mM TCEP) in the dark at room temperature for 1 h. The sample was added 4 volumes of acetone, chilled to −20 °C, vortexed, and then incubated for 3 h at −20 °C to completely precipitate the proteins and remove unreacted biotin-azide. The sample was centrifuged at 17,000*g* for 15 min at 4 °C to pellet the precipitated proteins. The resulting protein pellets were resolved in 1% SDS by sonication, PBS was added to dilute the concentration of SDS to 0.2% and then added 60 µl pre-washed high-capacity streptavidin C1 beads, followed by incubation overnight at 4 °C with rotation. The beads were washed 3 times with 0.1% SDS in PBS and once with PBS, 2× SDS sample buffer was added and boiled for western blot analysis of TVA-binding proteins.

### Co-culture assay with Blinatumomab

The RS4;11 target cells were stained using the CellTrace Far Red Cell Proliferation Kit (Invitrogen, C34564), and then co-cultured for 24 h with PBMC in flat bottom 24-well plate at a 1:5 ratio (2 × 10^5^ target cells to 10^6^ PBMC) at indicated concentrations of Blinatumomab (0, 10, 100, 1,000 pg ml^−1^) derived from the leftover of infusions (provided by the laboratory of W.S.). The cell mixture was re-suspended in a flow cytometry staining buffer, stained using a Fixable Viability Dye780 (R&D), and then analysed by flow cytometry.

### CAR-T cell expansion assay

In total, 63,000 anti-human CD19-CD28z-GFP CAR-T cells (provided by the laboratory of J.K.) were cultured in T cell expansion medium (StemCell) with IL-7 (5 ng ml^−1^) and IL-15 (5 ng ml^−1^) in the presence or absence of 20 μM TVA. Medium was changed to fresh medium (with IL-7 and IL-15 in the presence or absence of 20 μM TVA) in Day 2, 5, 7 and 9. Cell number was counted on the days 7, 9 and 10.

### Quantification and statistical analysis

All statistical analyses were performed using GraphPad Prism 9. Unpaired two-sided Student’s *t*-test was subjected to data statistical analysis, except a two-way ANOVA test was performed for cell proliferation assay and tumour growth analysis. *P* values less than 0.05 were considered significant. Data with error bars represent mean ± s.d., except for cell proliferation and tumour growth curves which represent mean ± s.e.m. All data figures are representative of at least three independent experiments, or two independent experiments (Extended Data Fig. 4h, right). All attempts at replication were successful.

### Reporting summary

Further information on research design is available in the Nature Portfolio Reporting Summary linked to this article.

## Online content

Any methods, additional references, Nature Portfolio reporting summaries, source data, extended data, supplementary information, acknowledgements, peer review information; details of author contributions and competing interests; and statements of data and code availability are available at 10.1038/s41586-023-06749-3.

### Supplementary information

Supplementary Information This file contains Supplementary Figs. 1 and 2. Supplementary Fig. 1: Gating strategies for flow cytometry analysis of intratumoral immune cells from tumours (**a**) and immune cells from spleen or dLN (**b**). Supplementary Fig. 2: Uncropped immunoblot images with size marker indications.

Reporting Summary

Supplementary Table 1 Summary of human circulating nutrients (633 in total).

Supplementary Table 2 Circulating nutrient library for screens.

Supplementary Table 3 1st screen 1a summary.

Supplementary Table 4 1st screen 1b summary. Student’s two-sided unpaired *t*-test.

Supplementary Table 5 Summary of second screen results.

Supplementary Table 6 Phospho-antibody array- TVA 40 min, 2 h, 6 h, 24 h. Student’s two-sided unpaired *t*-test.

Supplementary Table 7 DEGs only in siNTC + TVA group.

Supplementary Table 8 CAR-T therapy patient sample information.

Supplementary Table 9 Diet formula of control, TVA and CVA diets.

### Source data

Source Data Fig. 1

Source Data Fig. 3

Source Data Fig. 4

Source Data Fig. 5

Source Data Extended Data Fig. 1

Source Data Extended Data Fig. 2

Source Data Extended Data Fig. 3

Source Data Extended Data Fig. 4

Source Data Extended Data Fig. 5

Source Data Extended Data Fig. 6

Source Data Extended Data Fig. 7

Source Data Extended Data Fig. 8

Source Data Extended Data Fig. 9

Source Data Extended Data Fig. 10

Source Data Extended Data Fig. 11

Source Data Extended Data Fig. 12

## Data Availability

The 16S amplicon sequencing data have been deposited at the Gene Expression Omnibus (GEO) with the accession number GSE202266. The KAS-seq data have been deposited at the GEO repository with the accession number GSE202730. The RNA-seq data have been deposited at the GEO repository with the accession number GSE202276 and GSE202274. All data supporting the findings of this study are available within the Article and its Supplementary Information. Source data are provided with this paper.
