# Supplementary material for: Trans-vaccenic acid reprograms CD8+ T cells and anti-tumour immunity
Source: Nature. 2023 Nov 22;623(7989):1034–43. doi: 10.1038/s41586-023-06749-3 (PMC10686835; doi:10.1038/s41586-023-06749-3)
Supplement: Supplementary file 1 — This file contains Supplementary Figs. 1 and 2. Supplementary Fig. 1: Gating strategies for flow cytometry analysis of intratumoral immune cells from tumours (a) and immune cells from spleen or dLN (b). Supplementary Fig. 2: Uncropped immunoblot images with size marker indications. [file 41586_2023_6749_MOESM1_ESM.pdf]

nature portfolio

https://doi.org/10.1038/s41586-023-06749-3

Supplementary information

Trans-vaccenic acid reprograms $CD8^+$ T cells and anti-tumour immunity

In the format provided by the
authors and unedited

Supplementary Figure 1. Gating strategies for flow cytometry analysis

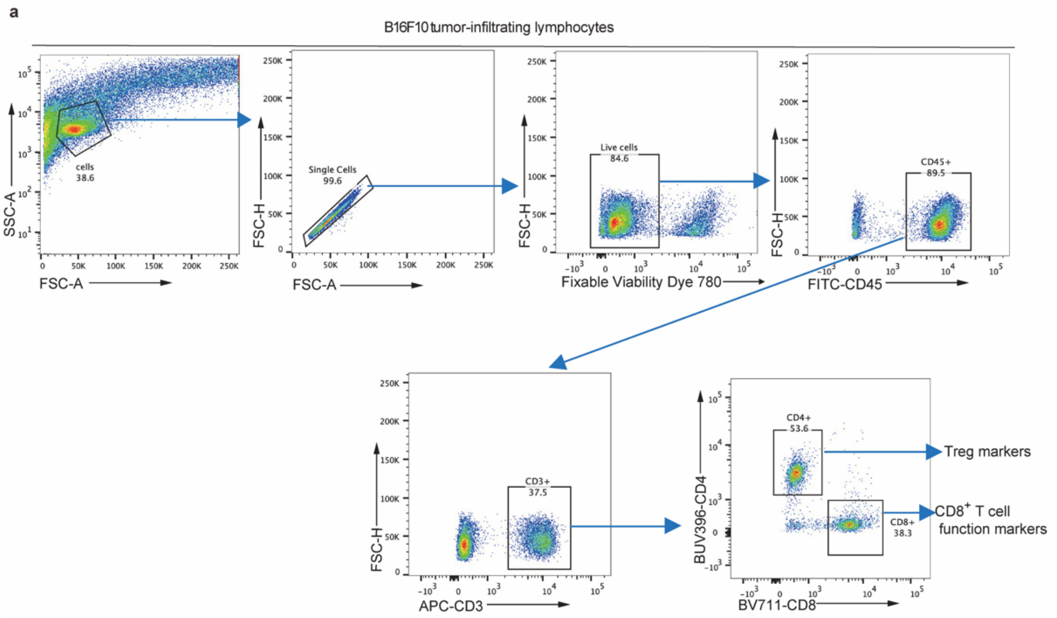

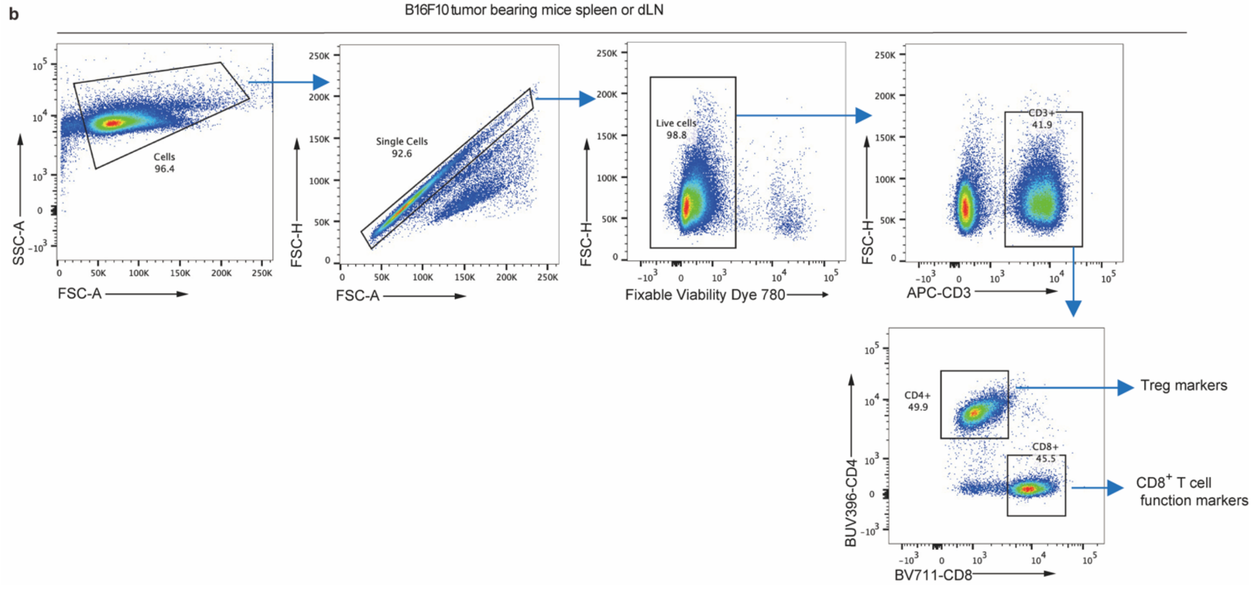

Supplementary Figure 2. Uncropped immunoblot images with size marker indications

Fig. 4c

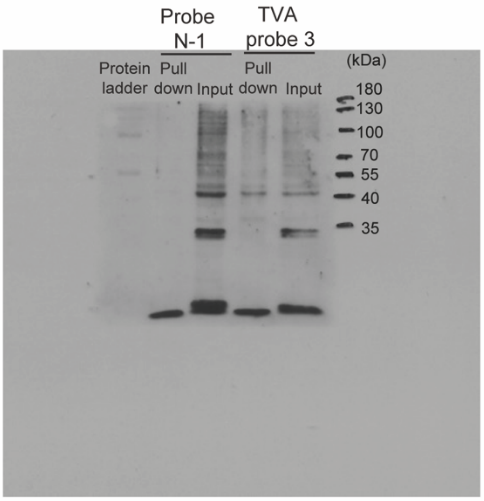

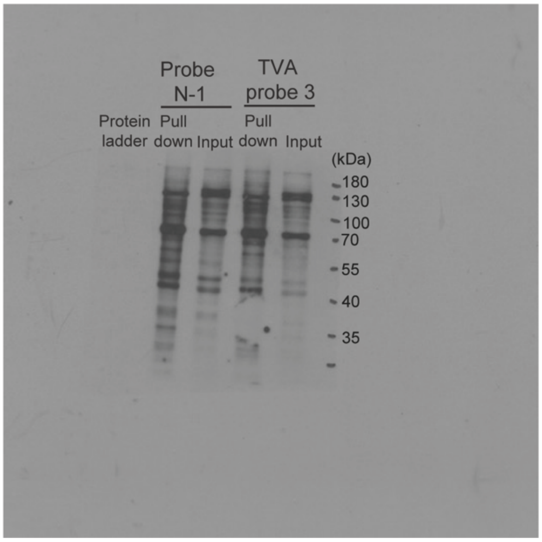

Extended Data Fig. 6e

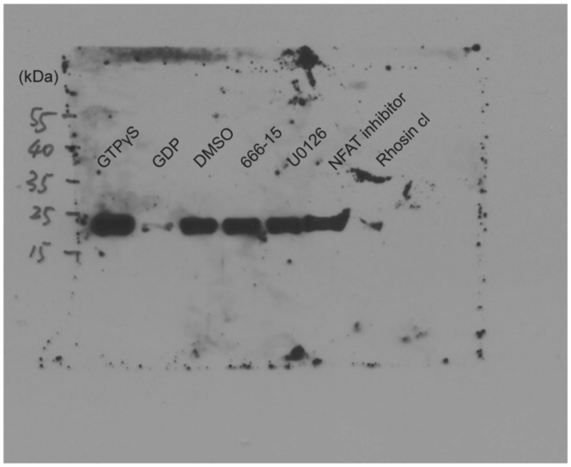

TVA derivatives and probes synthetic procedures

Synthesis of compound 9

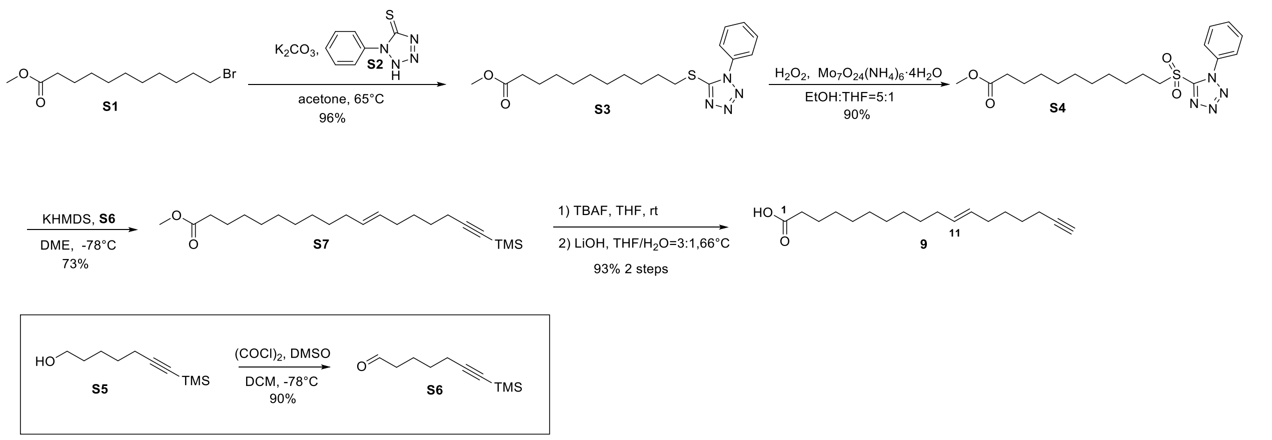

Scheme 1. Synthesis of compound 9

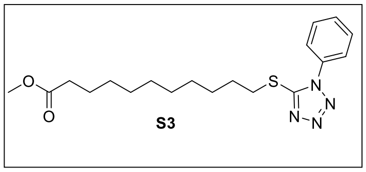

The bromide **S1** (1.0 g, 3.6 mmol) was added to a stirred solution of 1- phenyl-1H-tetrazol-5-thiol **S2** (1.14 g, 6.4 mmol) and potassium carbonate (1.0 g, 7.2 mmol) in acetone (20 mL) at room temperature. The mixture was heated to 65°C and stirred for 2.5 h, then cooled to room temperature, the mixture was filtered, then the solvent was evaporated, and the residue was diluted with a mixture of DCM (100 mL) and water (20 mL). The organic layer was separated, and the aqueous layer was extracted with the DCM (2×50 mL). The combined organic layers were dried over $NaSO_4$ and evaporated. Silica gel column chromatography (EtOAc: hexane =1:6) gave a white solid (1.4 g, 96%). Obtained characterization data were in agreement with those published in the literature[1].

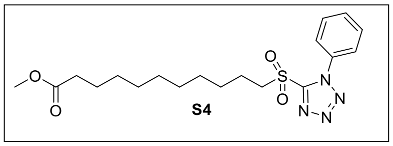

To a solution of sulfide **S3** (500 mg, 1.2 mmol) in EtOH/THF = 5:1 (12 mL) was added a solution of $(NH4)_6Mo7O_{24}\cdot 4H_2O$ (293.8 mg, 0.23 mmol) in H_2O_2 (2.6 mL, 30%) at 0 °C. The reaction was stirred at room temperature overnight and quenched with H_2O (5.0 mL). The mixture was extracted with DCM (3×20 mL). The combined organic phases were dried over Na_2SO_4 and evaporated. Purification by silica gel column chromatography (EtOAc: hexane = 1:5) gave a white solid (448 mg, 90%). Obtained characterization data were in agreement with those published in the literature[1].

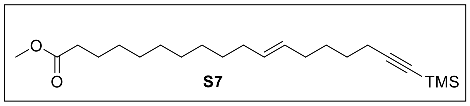

To a stirred solution of the **S4** (100 mg, 0.25 mmol) in DME (1.6 mL) at -78°C under nitrogen was added dropwise the KHMDS (0.5 M in toluene, 0.54 mL, 0.27 mmol). The mixture was then stirred for 30 min before addition of the aldehyde **S6** (prepared as reported, 45 mg, 0.25 mmol). After stirring for a further 1 h at -78°C the reaction mixture was quenched with sat. NH_4Cl (1.0 mL), then the mixture was extracted with EtOAc (3×10 mL). The combined organic phases were dried over Na_2SO_4 and evaporated. Purification by silica gel column chromatography (EtOAc: hexane = 100:1) gave a colourless oil (65 mg, 73%). ^{1}H NMR (400 MHz, $CDCl_3$) δ 5.38 (q, *J* = 5.1 Hz, 2H), 3.66 (s, 3H), 2.29 (t, *J* = 7.5 Hz, 2H), 2.21 (t, *J* = 6.9 Hz, 2H), 1.97 (h, *J* = 5.7 Hz, 4H), 1.60 (m, 2H), 1.56 – 1.37 (m, 4H), 1.28 (m, 12H), 0.14 (s, 9H). ^{13}C NMR (101 MHz, $CDCl_3$) δ 174.5, 130.9, 129.9, 107.8, 84.4, 51.6, 34.3, 32.7, 32.1, 29.7, 29.6, 29.5, 29.4, 29.3, 29.3, 28.9, 28.2, 25.1, 19.9, 0.3.

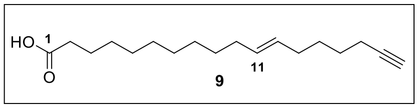

To a solution of sulfide **S7** (21 mg, 0.057 mmol) in THF (2.0 mL) was added a solution of TBAF (1.0 M in THF, 86 µL, 0.086 mmol) at room temperature. The reaction was stirred at room temperature for 30 min then quenched with H_2O (2.0 mL). The mixture was extracted with EtOAc (3×10 mL). The combined organic phases were dried over Na_2SO_4 and concentrated in vacuo to yield the product which was used directly in the follow reaction. The above product was dissolved in THF: H_2O=3:1 (0.8 mL), then LiOH (14 mg, 0.34 mmol) was added into the above solution, heated the mixture to 66°C and stirred for 3 h, cooled to room temperature, 2M HCl was added to the mixture to adjust the pH to 2, then the mixture was extracted with EtOAc (3×5.0 mL). The combined organic phases were dried over Na_2SO_4 and evaporated. Purification by silica gel column chromatography (EtOAc: hexane = 1:5) gave a white solid (15 mg, 93% for 2 steps). ^{1}H NMR (400 MHz, $CDCl_3$) δ 5.48 – 5.27 (m, 2H), 2.35 (t, *J* = 7.5 Hz, 2H), 2.18 (td, *J* = 6.9, 2.7 Hz, 2H), 2.08 – 1.91 (m, 5H), 1.69 – 1.58 (m, 2H), 1.57 – 1.40 (m, 4H), 1.39 – 1.27 (m, 12H). ^{13}C NMR (101 MHz, $CDCl_3$) δ 180.2, 131.0, 129.8, 84.8, 68.3, 34.2, 32.7, 32.1, 29.8, 29.7, 29.6, 29.5, 29.4, 29.3, 29.2, 28.8, 28.1, 24.8, 18.4. HRMS-ESI (m/z): calcd. for $C_{18}H_{30}O_2H^+$ $[M+H]^+$: 279.2319, found 279.2317.

Synthesis of compound 10

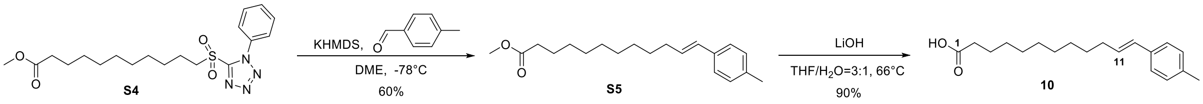

Scheme 2. Synthesis of compound 10

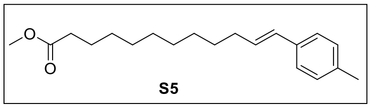

To a stirred solution of the **S4** (50 mg, 0.12 mmol) in DME (1.6 mL) at -78°C under nitrogen was added dropwise the KHMDS (0.5 M in toluene, 0.27 mL, 0.14 mmol) The mixture was then stirred for 30 min before addition of the p-tolualdehyde (16 mg, 0.27 mmol). After stirring for a further 1 h at -78°C the reaction mixture was quenched with sat. NH_4Cl (1.0 mL), then the mixture was extracted with EtOAc (3×10 mL). The combined organic phases were dried over Na_2SO_4 and evaporated. Purification by silica gel column chromatography (EtOAc: hexane = 60:1) gave a colorless oil (22 mg, 60%).

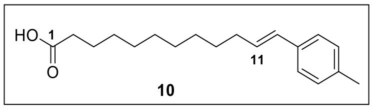

The above product **S5** (12 mg, 0.039mmol) was dissolved in THF:H_2O=3:1 (0.8 mL), then LiOH (7.0 mg, 0.16 mmol) was added into the above solution, heated the mixture to 66°C and stirred for 3 h, cooled to room temperature, 2M HCl was added to the mixture to adjust the pH to 2, then the mixture was extracted with EtOAc (3×5.0 mL). The combined organic phases were dried over Na_2SO_4 and evaporated. Purification by silica gel column chromatography (EtOAc: hexane = 1:6) gave a white solid (10 mg, 90%). ^{1}H NMR (400 MHz, $CDCl_3$) δ 7.23 (d, *J* = 7.9 Hz, 2H), 7.09 (d, *J* = 7.8 Hz, 2H), 6.34 (d, *J* = 15.8 Hz, 1H), 6.16 (dt, *J* = 15.8, 6.9 Hz, 1H), 2.36 (d, *J* = 7.6 Hz, 2H), 2.32 (d, *J* = 2.9 Hz, 3H), 2.18 (q, *J* = 6.5 Hz, 2H), 1.63 (p, *J* = 7.4 Hz, 2H), 1.45 (p, *J* = 7.0 Hz, 2H), 1.30 (s, 10H). ^{13}C NMR (101 MHz, $CDCl_3$) δ 179.6, 136.6, 135.3, 130.3, 129.7, 129.3, 125.9, 34.1, 33.2, 29.9, 29.6, 29.6, 29.4, 29.3, 29.2, 24.8, 21.3. HRMS-ESI (m/z): calcd. for $C_{19}H_{28}O_2H^+$ $[M+H]^+$: 289.2162, found 289.2149.

Synthesis of compound 11

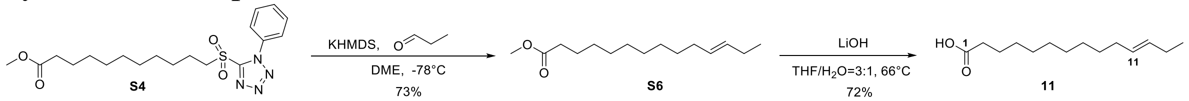

Scheme 3. Synthesis of compound 11

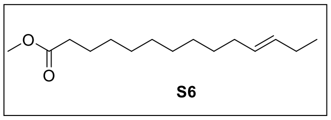

To a stirred solution of the **S4** (100 mg, 0.25 mmol) in DME (1.6 mL) at -78°C under nitrogen was added dropwise the KHMDS (0.5 M in toluene, 0.54 mL, 0.28 mmol) The mixture was then stirred for 30 min before addition of the propionaldehyde (16 mg, 0.27 mmol). After stirring for a further 1 h at -78°C the reaction mixture was quenched with sat. NH_4Cl (1.0 mL), then the mixture was extracted with EtOAc (3×10 mL). The combined organic phases were dried over Na_2SO_4 and evaporated. Purification by silica gel column chromatography (EtOAc: hexane = 1:60) gave a colorless oil (43 mg, 73%).^{1}H NMR (400 MHz, $CDCl_3$) δ 5.49 – 5.30 (m, 2H), 3.66 (s, 3H), 2.29 (t, *J* = 7.5 Hz, 2H), 1.98 (dqd, *J* = 14.8, 6.9, 5.1 Hz, 4H), 1.61 (h, *J* = 7.5 Hz, 2H), 1.38 – 1.20 (m, 12H), 1.02 – 0.85 (m, 3H). ^{13}C NMR (101 MHz, $CDCl_3$) δ 174.5, 132.0, 129.5, 51.6, 34.3, 32.7, 29.8, 29.6, 29.5, 29.4, 29.3, 29.3, 25.7, 25.1, 14.1. HRMS-ESI (m/z): calcd. for $C_{15}H_{28}O_2Na^+$ $[M+Na]^+$: 263.1982, found 263.1978.

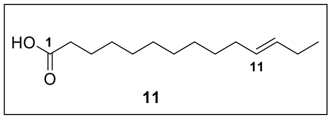

The above product **S6** (28 mg, 0.12mmol) was dissolved in THF:H_2O=3:1 (1.2 mL), then LiOH (20 mg, 0.46 mmol) was added into the above solution, heated the mixture to 66°C and stirred for 3 h, cooled to room temperature, 2M HCl was added to the mixture to adjust the pH to 2, then the mixture was extracted with EtOAc (3×5.0 mL). The combined organic phases were dried over Na_2SO_4 and evaporated. Purification by silica gel column chromatography (EtOAc : hexane = 1:10 - 1:5 - 1:1) gave a white solid (20 mg, 72%).^{1}H NMR (400 MHz, $CDCl_3$) δ 5.50 – 5.31 (m, 2H), 2.35 (t, *J* = 7.5 Hz, 2H), 1.98 (dq, *J* = 13.9, 7.2 Hz, 4H), 1.63 (p, *J* = 7.3 Hz, 2H), 1.40 – 1.26 (m, 12H), 0.96 (t, *J* = 7.4 Hz, 3H).

^{13}C NMR (101 MHz, $CDCl_3$) δ 180.4, 132.0, 129.5, 34.2, 32.7, 29.9, 29.8, 29.6, 29.5, 29.4, 29.3, 29.2, 25.7, 24.8, 14.1. . HRMS-ESI (m/z): calcd. for $C_{14}H_{26}O_2H^+$ $[M+H]^+$: 249.1825, found 249.1821.

Synthesis of compound 13

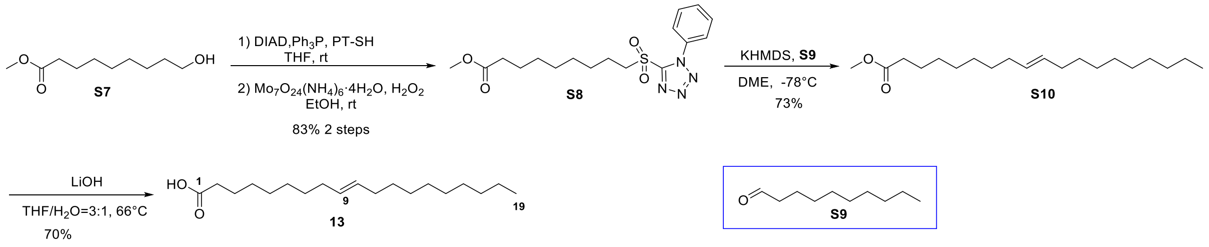

Scheme 4. Synthesis of compound 13

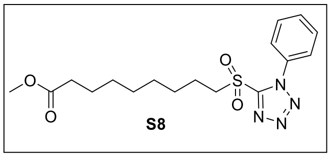

A solution of PT-SH (568 mg, 3.2 mmol), PPh_3 (836 mg, 3.2 mmol) and alcohol **S7** (500 mg, 2.7 mmol) in THF (13 mL) and DEAD (644 mg, 3.2 mmol) was added. The resulting solution was stirred for 3 h at room temperature. The resulting solution was diluted with EtOH (20 mL), cooled to 0°C and $(NH4)_6Mo7O_{24}·4H_2O$ (641 mg, 0.51 mmol) in H_2O_2 (5.8 mL, 30%) were added. The resulting yellowish solution was allowed to warm to room temperature and stirred for 10 h. Water (20 mL) was added and the whole mixture was extracted with EtOAc (3x100 mL). The combined organic layers were washed with brine (20 mL), dried over Na_2SO_4, filtered and the solvents were evaporated under reduced pressure. Purification by silica gel column chromatography (EtOAc : hexane = 1:5) gave a white solid (900 mg, 83% for 2 steps). ^{1}H NMR (400 MHz, $CDCl_3$) δ 7.70 (ddt, *J* = 7.2, 3.4, 2.5 Hz, 2H), 7.65 – 7.56 (m, 3H), 3.81 – 3.70 (m, 2H), 3.66 (s, 3H), 2.30 (t, *J* = 7.5 Hz, 2H), 2.01 – 1.89 (m, 2H), 1.68 – 1.44 (m, 4H), 1.33 (qd, *J* = 8.8, 4.5 Hz, 6H). ^{13}C NMR (101 MHz, $CDCl_3$) δ 174.3, 153.6, 133.2, 131.6, 129.8, 125.2, 56.1, 51.6, 34.1, 29.0, 28.9, 28.8, 28.2, 24.9, 22.1. HRMS-ESI (m/z): calcd. for $C_{17}H_{24}N_4O_4SH^+$ $[M+H]^+$: 381.1591, found 381.1600.

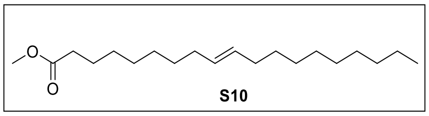

Compounds **S10** was synthesized following a similar procedure described for **S6**. Without purified for the next step.

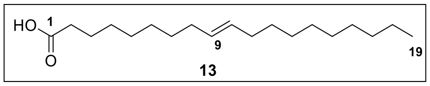

Compounds **13** was synthesized following a similar procedure described for **11**. White solid, yield: 70%.^{1}H NMR (400 MHz, $CDCl_3$) δ 5.41 – 5.34 (m, 2H), 2.34 (t, *J* = 7.5 Hz, 2H), 1.96 (q, *J* = 6.2 Hz, 4H), 1.63 (p, *J* = 7.2 Hz, 2H), 1.44 – 1.15 (m, 22H), 0.88 (t, *J* = 6.7 Hz, 3H). ^{13}C NMR (101 MHz, $CDCl_3$) δ 180.0, 130.5, 130.2, 34.0, 32.6, 32.6, 31.9, 29.7, 29.6, 29.6, 29.6, 29.4, 29.2, 29.1, 29.0, 28.9, 24.7, 22.7, 14.1. HRMS-ESI (m/z): calcd. for $C_{19}H_{36}O_2H^+$ $[M+H]^+$: 297.2788, found 297.2777.

Synthesis of compound 14

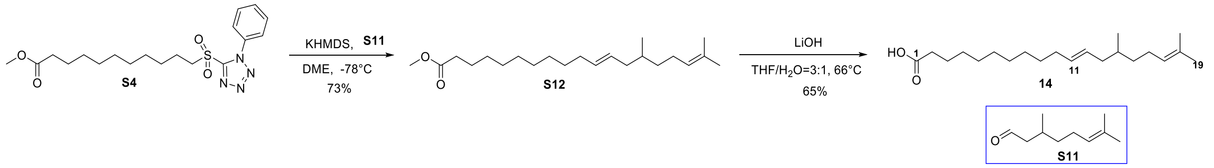

Scheme 5. Synthesis of compound 14

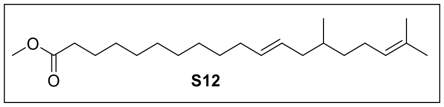

Compounds **S12** was synthesized following a similar procedure described for **S6**. Without purified for the next step.

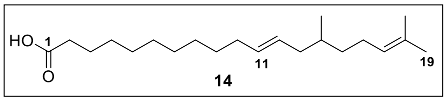

Compounds **14** was synthesized following a similar procedure described for **11**. White solid, yield: 65%.^{1}H NMR (400 MHz, $CDCl_3$) δ 5.44 – 5.29 (m, 2H), 5.10 (ddt, *J* = 8.6, 7.1, 1.4 Hz, 1H), 2.35 (t, *J* = 7.5 Hz, 2H), 1.97 (dh, *J* = 10.2, 4.4 Hz, 5H), 1.87 – 1.77 (m, 1H), 1.68 (s, 3H), 1.65 – 1.62 (m, 1H), 1.60 (s, 3H), 1.49 – 1.40 (m, 1H), 1.37 – 1.24 (m, 16H), 0.86 (d, *J* = 6.6 Hz, 3H). ^{13}C NMR (101 MHz, $CDCl_3$) δ 180.0, 131.7, 131.0, 128.7, 125.0, 40.0, 36.6, 34.0, 32.8, 32.6, 29.7, 29.6, 29.4, 29.4, 29.2, 29.1, 29.1, 25.7, 25.6, 24.7, 19.4, 17.6. HRMS-ESI (m/z): calcd. for $C_{21}H_{38}O_2H^+$ $[M+H]^+$: 323.2945, found 323.2947.

Synthesis of compound 15

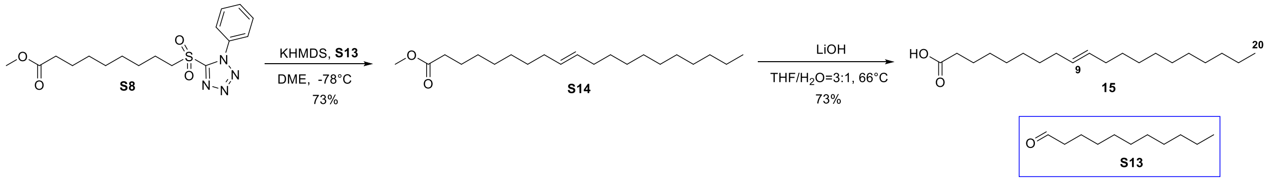

Scheme 6. Synthesis of compound 15

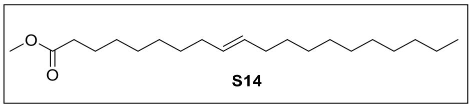

Compounds **S14** was synthesized following a similar procedure described for **S6**. Without purified for the next step.

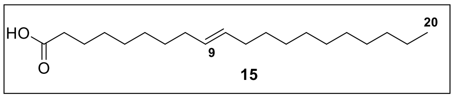

Compounds **15** was synthesized following a similar procedure described for **11**. White solid, yield: 73%.^{1}H NMR (400 MHz, $CDCl_3$) δ 5.42 – 5.34 (m, 2H), 2.40 – 2.30 (m, 2H), 2.01 – 1.92 (m, 4H), 1.63 (p, *J* = 7.4 Hz, 2H), 1.38 – 1.21 (m, 24H), 0.88 (t, *J* = 1.9 Hz, 3H). ^{13}C NMR (101 MHz, $CDCl_3$) δ 180.0, 130.5, 130.2, 34.0, 32.6, 32.6, 31.9, 29.7, 29.6, 29.5, 29.4, 29.2, 29.1, 29.0, 28.9, 24.7, 22.7, 14.1. HRMS-ESI (m/z): calcd. for $C_{20}H_{38}O_2H^+$ $[M+H]^+$: 311.2945, found 311.2938.

Synthesis of compound 16

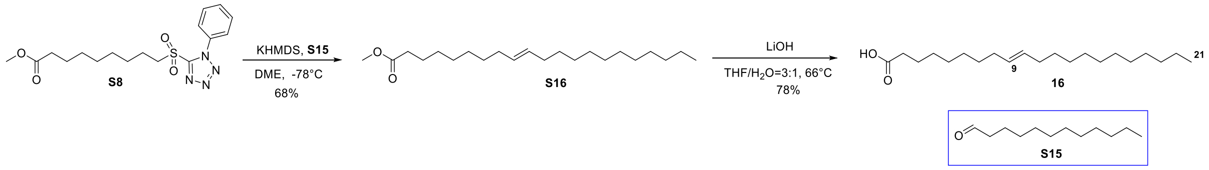

Scheme 7. Synthesis of compound 16

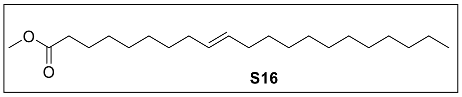

Compounds **S16** was synthesized following a similar procedure described for **S6**. Without purified for the next step.

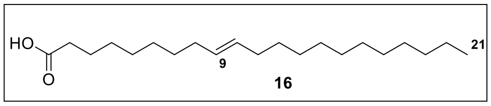

Compounds **16** was synthesized following a similar procedure described for **11**. White solid, yield: 78%.^{1}H NMR (400 MHz, $CDCl_3$) δ 5.42 – 5.34 (m, 2H), 2.35 (ddd, *J* = 9.2, 6.7, 1.5 Hz, 2H), 2.01 – 1.92 (m, 4H), 1.63 (p, *J* = 7.4 Hz, 2H), 1.37 – 1.25 (m,26H), 0.92 – 0.84 (m, 3H). 13C NMR (101 MHz, CDCl3) δ 179.8, 130.5, 130.2, 34.0, 32.6, 32.6, 31.9, 29.7, 29.7, 29.6, 29.5, 29.4, 29.2, 29.1, 29.0, 28.9, 24.7, 22.7, 14.1. HRMS-ESI (m/z): calcd. for $C_{21}H_{40}O_2H^+$ $[M+H]^+$: 325.3101, found 325.3097.

Synthesis of compound 17

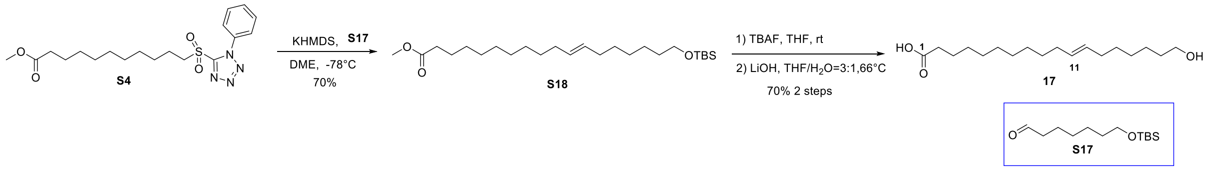

Scheme 8. Synthesis of compound 17

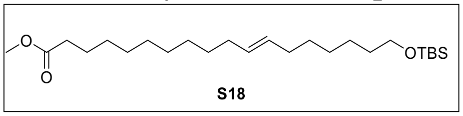

To a stirred solution of the **S4** (80 mg, 0.20 mmol) in DME (1.3 mL) at -78°C under nitrogen was added dropwise the KHMDS (0.5 M in toluene, 0.43 mL, 0.22 mmol) The mixture was then stirred for 30 min before addition of the aldehyde **S17** (prepared as reported, 53 mg, 0.22 mmol). After stirring for a further 1 h at -78°C the reaction mixture was quenched with sat. NH_4Cl (1.0 mL), then the mixture was extracted with EtOAc (3×10 mL). The combined organic phases were dried over Na_2SO_4 and evaporated. Without purified for the next step.

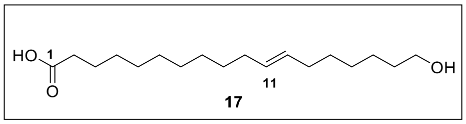

To a solution of sulfide **S18** (60 mg, 0.15 mmol) in THF (1.5 mL) was added a solution of TBAF (1.0 M in THF, 181 μL, 0.18 mmol) at room temperature. The reaction was stirred at room temperature for

30 min then quenched with H_2O (2.0 mL). The mixture was extracted with EtOAc (3×10 mL). The combined organic phases were dried over Na_2SO_4 and concentrated in vacuo to yield the product which was used directly in the follow reaction. The above product was dissolved in THF:H_2O=3:1 (2.4 mL), then LiOH (25 mg, 0.60 mmol) was added into the above solution, heated the mixture to 66°C and stirred for 3 h, cooled to room temperature, 2M HCl was added to the mixture to adjust the pH to 2, then the mixture was extracted with EtOAc (3×5.0 mL). The combined organic phases were dried over Na_2SO_4 and evaporated. Purification by silica gel column chromatography (EtOAc: hexane = 1:4 - 1:1) gave a white solid (31 mg, 70% for 2 steps). ^{1}H NMR (400 MHz, $CDCl_3$) δ 5.43 – 5.32 (m, 2H), 3.64 (t, *J* = 6.6 Hz, 2H), 2.34 (t, *J* = 7.5 Hz, 2H), 2.04 – 1.92 (m, 4H), 1.69 – 1.51 (m, 5H), 1.41 – 1.20 (m, 19H). ^{13}C NMR (101 MHz, $CDCl_3$) δ 179.3, 130.5, 130.2, 63.0, 34.0, 32.7, 32.5, 32.5, 29.6, 29.5, 29.4, 29.2, 29.0, 29.02, 29.0, 25.6, 24.7. HRMS-ESI (m/z): calcd. for $C_{18}H_{34}O_3H^+$ $[M+H]^+$: 299.2581, found 299.2582.

Synthesis of probe-1

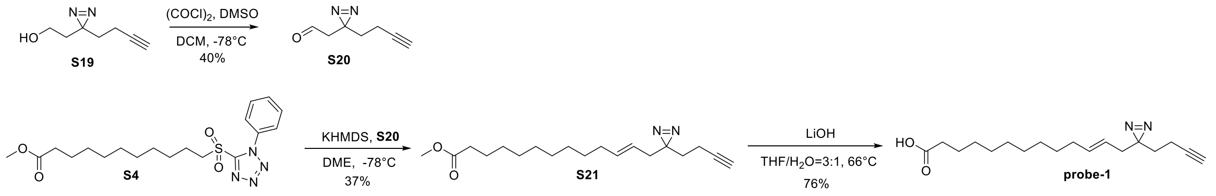

Scheme 9. Synthesis of probe-1

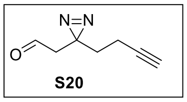

Oxalyl chloride (46 µL, 0.54 mmol) was dissolved in 2.0 mL DCM and brought to -78 °C. A solution of DMSO (78 µL, 1.08 mmol) in DCM (1.0 mL) was added dropwise, and the reaction was allowed to stir for 15 min. A solution of the alcohol **S19** (50 mg, 0.36 mmol) in DCM (1.0 mL) was added dropwise, and the reaction was allowed to stir an additional 15 min. Then Et_3N (300 µL, 2.2 mmol) was added dropwise. After 15 min, the reaction was allowed to warm to room temperature. The reaction mixture was transferred to a separatory funnel and washed with H_2O (2 mL). The phases were separated, and the aqueous phase was extracted with DCM (3×5.0 mL). The combined organic extracts were dried over Na_2SO_4, and concentrated. Purification by silica gel column chromatography (EtOAc: hexane = 1:6) gave a brown oil (20 mg, 40%).^{1}H NMR (400 MHz, $CDCl_3$) δ 9.74 (s, 1H), 2.46 (d, *J* = 1.6 Hz, 2H), 2.06 (td, *J* = 7.3, 2.7 Hz, 2H), 2.01 (d, *J* = 2.6 Hz, 1H), 1.73 (t, *J* = 7.2 Hz, 2H). ^{13}C NMR (101 MHz, $CDCl_3$) δ 197.5, 82.5, 69.8, 48.4, 32.4, 24.5, 13.3.

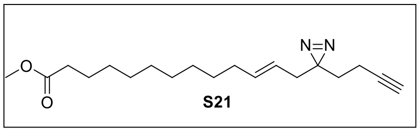

To a stirred solution of the **S4** (40 mg, 0.10 mmol) in DME (0.50 mL) at -78°C under nitrogen was added dropwise the KHMDS (0.5 M in toluene, 0.19 mL, 0.10 mmol) The mixture was then stirred for 30 min before addition of the aldehyde **S20** (12 mg, 0.09 mmol). After stirring for a further 1 h at -78°C the reaction mixture was quenched with sat. NH_4Cl (1.0 mL), then the mixture was extracted with EtOAc (3×10 mL). The combined organic phases were dried over Na_2SO_4 and evaporated. Purification by silica gel column chromatography (EtOAc: hexane = 1:30) gave a colorless oil (10 mg, 37%).^{1}H NMR (400 MHz, $CDCl_3$) δ 5.56 – 5.42 (m, 1H), 5.25 – 5.12 (m, 1H), 3.66 (s, 3H), 2.30 (t, *J* = 7.6 Hz,

2H), 2.08 – 1.89 (m, 6H), 1.68 – 1.54 (m, 4H), 1.44 – 1.12 (m, 13H). ^{13}C NMR (101 MHz, $CDCl_3$) δ 174.5, 135.5, 122.1, 83.0, 69.1, 51.6, 37.0, 34.3, 32.7, 31.9, 29.5, 29.4, 29.3, 29.2, 28.3, 25.1, 13.4. HRMS-ESI (m/z): calcd. for $C_{19}H_{30}N_2O_2H^+$ $[M+H]^+$: 319.2380, found 319.2381.

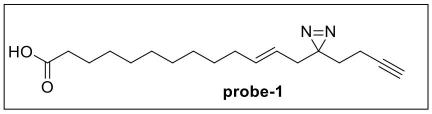

The above product **S21** (10 mg, 0.031mmol) was dissolved in THF:H_2O=3:1 (0.40 mL), then LiOH (5.4 mg, 0.13 mmol) was added into the above solution, heated the mixture to 66°C and stirred for 3 h, cooled to room temperature, 2M HCl was added to the mixture to adjust the pH to 2, then the mixture was extracted with EtOAc (3×5.0 mL). The combined organic phases were dried over Na_2SO_4 and evaporated. Purification by silica gel column chromatography (EtOAc : hexane = 1:3) gave a white solid (7.6 mg, 76%).^{1}H NMR (400 MHz, $CDCl_3$) δ 5.49 (dt, *J* = 15.3, 6.6 Hz, 1H), 5.25 – 5.13 (m, 1H), 2.35 (t, *J* = 7.5 Hz, 2H), 2.09 – 1.89 (m, 6H), 1.63 (td, *J* = 7.5, 3.9 Hz, 4H), 1.42 – 1.25 (m, 13H). ^{13}C NMR (101 MHz, $CDCl_3$) δ 179.7, 135.5, 122.1, 83.0, 69.1, 37.0, 32.7, 31.9, 29.8, 29.5, 29.5, 29.4, 29.3, 29.20, 28.3, 24.8, 13.4. HRMS-ESI (m/z): calcd. for $C_{18}H_{28}N_2O_2H^+$ $[M+H]^+$: 305.2224, found 305.2220.

Synthesis of probe-2

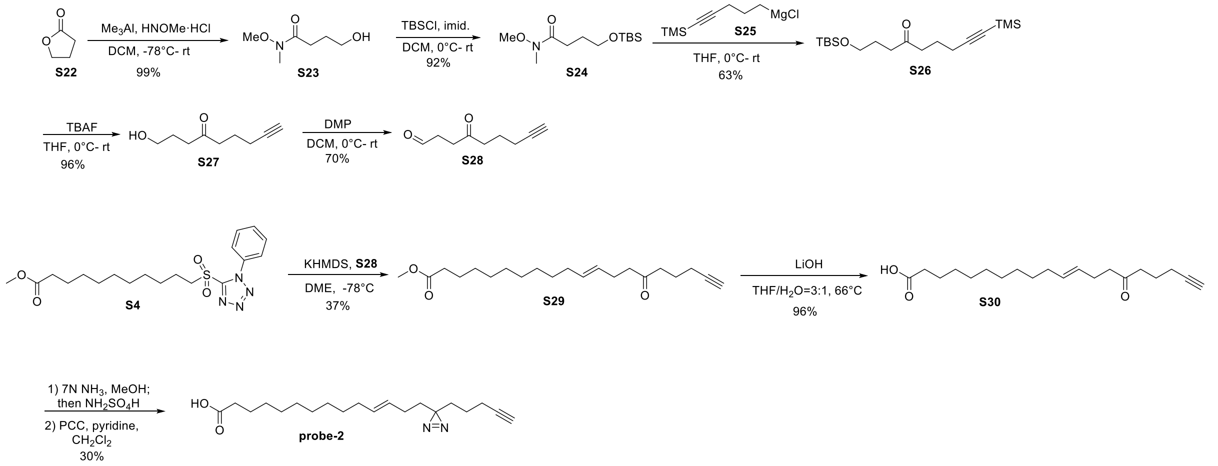

Scheme 10. Synthesis of probe-2

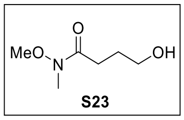

Trimethylaluminium (66 mL, 132 mmol, 2.0 M in toluene) was added over 1 h to a solution of N,O-dimethylhydroxylamine hydrochloride (13.6 g, 139.4 mmol) in DCM (50 mL) at -78 °C. The solution was warmed to room temperature and stirred for 4 h. The solution was then cooled to -5 °C, γ-butyrolactone **S22** (4.4 ml, 57.2 mmol) was added and the resulting mixture stirred for a further 1.5 h. After this time, the solution was carefully quenched at 0 °C by addition of a solution of potassium sodium L-tartrate tetrahydrate (16 g) in water (20 mL) and stirred overnight. The resulting precipitate was filtered through a plug of Celite and washed with DCM. The organic phase was dried over Na_2SO_4,

filtered, and the solvent removed in vacuo to give a light yellow oil (8.31 g, 99%). Obtained characterization data were in agreement with those published in the literature[2].

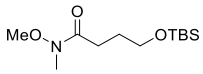

S24

To a solution of **S23** (3.4 g, 23.2 mmol) and imidazole (2.4 g, 34.8 mmol) in DCM (70 mL) was added TBSCl (3.8 g, 25.5 mmol) at 0 °C. The mixture was stirred at rt for 3 h. The mixture was then quenched by addition of H_2O (15 mL), and extracted with DCM (3 × 30 mL). The combined organic layers were washed with brine, dried over Na_2SO_4, and concentrated. The residue was purified by silica gel chromatography (EtOAc : hexane = 1:3) gave a colorless oil (5.6 g, 92%). Obtained characterization data were in agreement with those published in the literature[2].

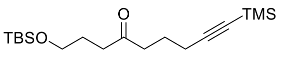

S26

Synthesis of (5-(trimethylsilyl)pent-4-yn-1-yl)magnesium chloride. This compound was prepared by following the reported procedure. Magnesium turnings (206 mg, 8.6 mmol) were etched with the back of a glass pipette and added to a flame-dried, two-neck RBF containing a stir bar and fitted with an oven-dried reflux condenser. After purging the reaction vessel with argon, a small bead of I_2 was added to the magnesium turnings followed by anhydrous THF (3.0 mL) and the resulting mixture was stirred for 15 min at room temperature. A few drops of (5-chloropent-1-yn-1yl)trimethylsilane (1.0 g, 5.7 mmol) dissolved in anhydrous THF (7.0 mL) was then added to the mixture and the mixture was heated to reflux. The remaining (5-chloropent-1yn-1-yl)trimethylsilane solution was then slowly added to the refluxing reaction mixture over 30 min. When the addition was complete, the reaction was refluxed for an additional 3 h before cooling to room temperature.

Compound **S24** (1.5 g, 5.7 mmol) was dissolved in anhydrous THF (30 mL) and cooled to 0 ºC under N_2. The above fresh prepared (5-(Trimethylsilyl)pent-4-yn-1-yl)magnesium chloride **S25** was then added dropwise and stirring for an additional 1 h at room temperature,. After stirring for 1 h, the reaction was quenched with the addition of sat. NH_4Cl (aq.) (10 mL) and the product was extracted with EtOAc (3 × 30 mL). The combined organic layers were washed with brine, then dried over Na_2SO_4 and concentrated under reduced pressure. The residue was purified by silica gel chromatography (EtOAc : hexane = 1:20) gave a yellow oil (1.2 g, 63%). ^{1}H NMR (400 MHz, $CDCl_3$) δ 3.60 (td, J = 5.7, 2.3 Hz, 2H), 2.59 – 2.45 (m, 4H), 2.25 (tt, J = 6.9, 1.2 Hz, 2H), 1.84 – 1.71 (m, 4H), 0.88 (s, 9H), 0.14 (s, 9H), 0.03 (s, 6H). ^{13}C NMR (101 MHz, $CDCl_3$) δ 210.2, 106.2, 85.2, 62.0, 41.1, 39.1, 26.7, 25.8, 22.3, 19.1, 18.2, -0.1, -5.5. HRMS-ESI (m/z): calcd. for $C_{18}H_{36}O_2Si_2H^+$ $[M+H]^+$: 341.2327, found 341.2331.

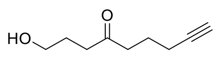

S27

To a solution of **S26** (300 mg, 0.88 mmol) in THF (5.0 mL) was added a solution of TBAF (1.0 M in THF, 2.2 mL, 2.2 mmol) at room temperature. The reaction was stirred at room temperature for 30 min then quenched with H_2O (5.0 mL). The mixture was extracted with EtOAc (3×20 mL). The combined organic phases were washed with brine, dried over Na_2SO_4 and concentrated in vacuo. The residue was purified by silica gel chromatography (EtOAc : hexane = 1:1) gave a yellow oil (130 mg, 96%). ^{1}H

NMR (400 MHz, $CDCl_3$) δ 3.64 (td, *J* = 6.1, 1.8 Hz, 2H), 2.59 (dtd, *J* = 8.9, 7.1, 1.7 Hz, 4H), 2.24 (ddd, *J* = 7.0, 4.7, 2.2 Hz, 2H), 2.16 (s, 1H), 1.97 (q, *J* = 2.1 Hz, 1H), 1.91 – 1.74 (m, 4H). ^{13}C NMR (101 MHz, $CDCl_3$) δ 210.9, 83.6, 69.1, 62.2, 41.2, 39.6, 26.5, 22.2, 17.7. HRMS-ESI (m/z): calcd. for $C_9H_{14}O_2H^+$ $[M+H]^+$: 155.1067, found 155.1067.

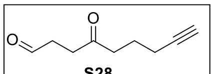

S28

Dess-Martin reagent (99 mg, 0.23 mmol) was added to the solution of **S27** (30 g, 0.2 mmol) in DCM (2.0 mL) at 0°C, and the mixture was stirred at room temperature for 4 hours. The reaction mixture was quenched with saturated $Na_2S_2O_3$: $NaHCO_3$ = 1:1 (5 mL), and the product was extracted with DCM (3×10 mL). The organic layer was washed with brine and dried over Na_2SO_4, concentrated in vacuo. The residue was purified by silica gel chromatography (EtOAc : hexane = 1:4) gave a yellow oil (20 mg, 70%). ^1H NMR (400 MHz, $CDCl_3$) δ 9.80 (s, 1H), 2.76 (p, *J* = 2.8 Hz, 4H), 2.64 (td, *J* = 7.2, 2.8 Hz, 2H), 2.24 (tt, *J* = 6.9, 2.3 Hz, 2H), 2.01 – 1.93 (m, 1H), 1.88 – 1.75 (m, 2H). ^{13}C NMR (101 MHz, $CDCl_3$) δ 208.0, 200.4, 83.5, 69.1, 41.0, 37.5, 34.8, 22.3, 17.7. HRMS-ESI (m/z): calcd. for $C_9H_{12}O_2Na^+$ $[M+Na]^+$: 175.0730, found 175.0735.

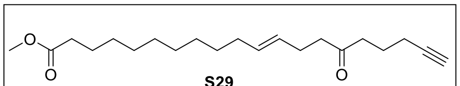

S29

To a stirred solution of the **S4** (54 mg, 0.13 mmol) in DME (2.0 mL) at -78°C under nitrogen was added dropwise the KHMDS (0.5 M in toluene, 0.26 mL, 0.13 mmol) The mixture was then stirred for 30 min before addition of the aldehyde **S28** (20 mg, 0.13 mmol). After stirring for a further 1 h at -78°C the reaction mixture was quenched with sat. NH_4Cl (2.0 mL), then the mixture was extracted with EtOAc (3×10 mL). The combined organic phases were washed with brine, dried over Na_2SO_4 and evaporated. Purification by silica gel column chromatography (EtOAc: hexane = 1:10) gave a yellow oil (16 mg, 37%). ^1H NMR (400 MHz, $CDCl_3$) δ 5.49 – 5.29 (m, 2H), 3.66 (s, 3H), 2.63 – 2.52 (m, 2H), 2.47 (t, *J* = 7.4 Hz, 2H), 2.34 – 2.16 (m, 6H), 2.06 – 1.90 (m, 3H), 1.79 (pd, *J* = 7.0, 1.0 Hz, 2H), 1.62 (p, *J* = 6.5 Hz, 2H), 1.37 – 1.23 (m, 12H). ^{13}C NMR (101 MHz, $CDCl_3$) δ 209.9, 174.3, 131.6, 128.2, 83.6, 69.0, 51.4, 42.8, 41.1, 34.1, 32.5, 29.5, 29.4, 29.4, 29.3, 29.1, 29.1, 26.9, 25.0, 22.2, 17.8. HRMS-ESI (m/z): calcd. for $C_{21}H_{34}O_3H^+$ $[M+H]^+$: 335.2581, found 335.2584.

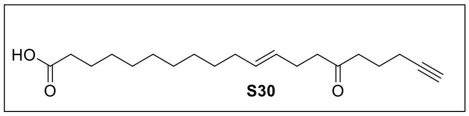

S30

The above product **S29** (16 mg, 0.048mmol) was dissolved in THF:H_2O=3:1 (0.40 mL), then LiOH (8.0 mg, 0.19 mmol) was added into the above solution, heated the mixture to 66°C and stirred for 3 h, cooled to room temperature, 2M HCl was added to the mixture to adjust the pH to 2, then the mixture was extracted with EtOAc (3×5.0 mL). The combined organic phases were washed with brine, dried over Na_2SO_4 and evaporated. Purification by silica gel column chromatography (EtOAc : hexane = 1:10) gave a white solid (15 mg, 96%). ^1H NMR (400 MHz, $CDCl_3$) δ 5.49 – 5.31 (m, 2H), 2.55 (t, *J* = 7.3 Hz, 2H), 2.48 (t, *J* = 7.5 Hz, 2H), 2.35 (t, *J* = 5.8 Hz, 2H), 2.30 – 2.16 (m, 4H), 2.06 – 1.90 (m, 3H), 1.85 – 1.73 (m, 2H), 1.63 (p, *J* = 7.6 Hz, 2H), 1.41 – 1.24 (m, 12H). ^{13}C NMR (101 MHz, $CDCl_3$) δ 210.0, 179.9, 131.6, 128.2, 83.6, 69.0, 42.8, 41.1, 34.0, 32.5, 29.4, 29.4, 29.4, 29.2, 29.1, 29.1, 26.9, 24.7, 22.2, 17.8. HRMS-ESI (m/z): calcd. for $C_{18}H_{32}O_3H^+$ $[M+H]^+$: 321.2424, found 321.2424.

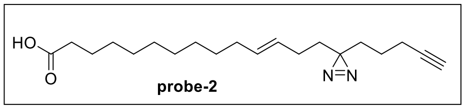

Ketone **S30** (16 mg, 0.050 mmol) was dissolved in a solution of NH_3 (7.0 N in MeOH, 0.19 mL, 1.3 mmol) at 0 ºC under N_2. After stirring for 3 h at 0 ºC, a solution of hydroxylamine-O-sulfonic acid (6.5 mg, 0.058 mmol) in MeOH (0.1 mL) was added dropwise. The reaction mixture was allowed to slowly warm to room temperature while stirring overnight. The reaction was then concentrated and the remaining residue was redissolved in anhydrous DCM (1.0 mL) and pyridine (0.1 mL) under the protection of N_2. PCC (11 mg, 0.05 mmol) was then added in small portions while the reaction mixture was cooled to 0 ºC. The reaction was then allowed to warm to room temperature and stirred for an additional 1 h, then 2 M HCl (1.0 mL) was added into above solution. The resulting solution was extracted with DCM (3×10 mL). The combined organic phases were washed with brine, dried over Na_2SO_4 and evaporated. Purification by silica gel column chromatography (EtOAc : hexane = 1:3) gave a oil (4.0 mg, 30%).^{1}H NMR (400 MHz, $CDCl_3$) δ 5.50 – 5.24 (m, 2H), 2.35 (t, J = 7.5 Hz, 2H), 2.16 (td, J = 7.0, 2.7 Hz, 2H), 2.02 – 1.91 (m, 3H), 1.79 (p, J = 6.6 Hz, 2H), 1.63 (p, J = 7.3 Hz, 2H), 1.56 – 1.34 (m, 2H), 1.34 – 1.17 (m, 16H). ^{13}C NMR (101 MHz, $CDCl_3$) δ 179.7, 131.7, 128.3, 83.4, 68.9, 34.0, 33.1, 32.6, 32.5, 31.8, 29.7, 29.6, 29.5, 29.5, 29.4, 29.4, 29.2, 29.1, 29.1, 28.3, 26.8, 24.7, 22.7, 18.0. HRMS-mixed (m/z): calcd. for $C_{20}H_{32}N_2O_2H^+$ $[M+H]^+$: 333.2537, found 333.2535.

Synthesis of probe-3

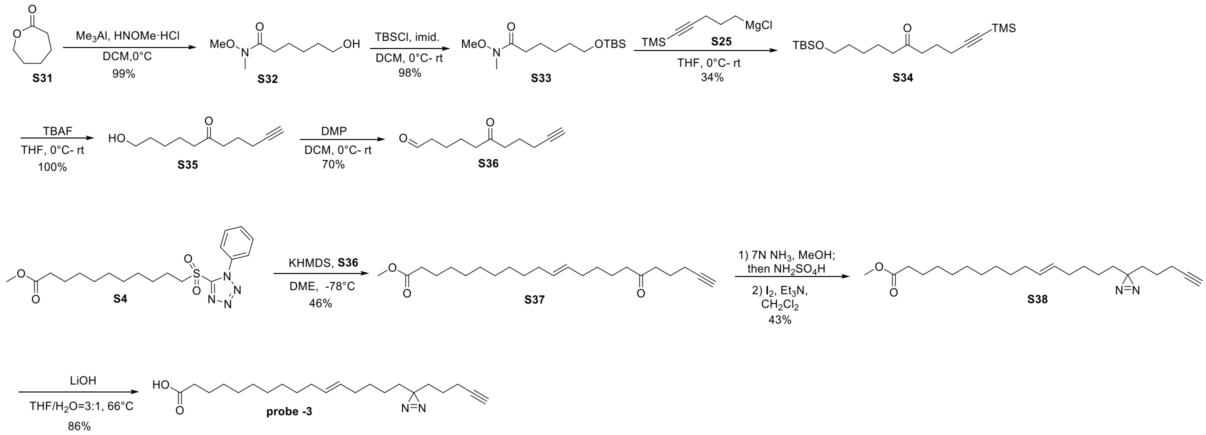

Scheme 11. Synthesis of probe-3

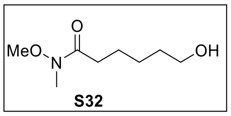

Trimethylaluminium (2.0 M in toluene, 7.5 mL, 35 mmol) was added dropwise to a solution of N,O-dimethylhydroxylamine hydrochloride (3.4 g, 35 mmol) and **S31** (2.0 g, 17.5 mmol) in DCM (60 mL) at 0 °C. The solution stirred for 24 h at 0 °C. After this time, the solution was carefully quenched at 0 °C by addition of a solution of potassium sodium L-tartrae tetrahydrate (3.9 g) in H_2O (5.9 mL). The resulting precipitate was filtered through a plug of Celite and washed with DCM. The organic phase was dried over Na_2SO_4, filtered, and the solvent removed in vacuo to give a light yellow oil (2.9 g, 99%). Obtained characterization data were in agreement with those published in the literature[3].

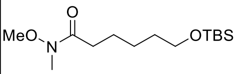

S33

To a solution of **S32** (2.9 g, 16.6 mmol) and imidazole (1.8 g, 26.4 mmol) in DCM (50 mL) was added TBSCl (2.8 g, 18.6 mmol) at 0 °C. The mixture was stirred at room temperature for 3 h. The mixture was then quenched by addition of H_2O (15 mL), and extracted with DCM (3 × 30 mL). The combined organic layers were washed with brine, dried over Na_2SO_4, and concentrated. The residue was purified by silica gel chromatography (EtOAc : hexane = 1:3) gave a colorless oil (4.7 g, 98%). Obtained characterization data were in agreement with those published in the literature[3].

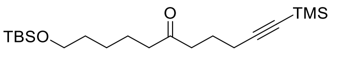

S34

Compound **S33** (1.6 g, 5.7 mmol) was dissolved in anhydrous THF (30 mL) and cooled to 0 ºC under N_2. The fresh prepared (5-(Trimethylsilyl)pent-4-yn-1-yl)magnesium chloride **S25** was then added dropwise and stirring for an additional 1 h at room temperature,. After stirring for 1 h, the reaction was quenched with the addition of sat. NH_4Cl (aq.) (10 mL) and the product was extracted with EtOAc (3 × 30 mL). The combined organic layers were washed with brine, then dried over Na_2SO_4 and concentrated under reduced pressure. The residue was purified by silica gel chromatography (EtOAc : hexane = 1:30) gave a yellow oil (0.7 g, 34%). ^{1}H NMR (400 MHz, $CDCl_3$) δ 3.45 (td, J = 6.5, 1.7 Hz, 2H), 2.39 (td, J = 7.3, 1.7 Hz, 2H), 2.28 (td, J = 7.5, 1.7 Hz, 2H), 2.11 (td, J = 6.9, 1.7 Hz, 2H), 1.63 (pd, J = 7.1, 1.7 Hz, 2H), 1.52 – 1.32 (m, 4H), 1.19 (ttd, J = 8.7, 6.3, 3.3 Hz, 2H), 0.74 (d, J = 1.7 Hz, 9H), 0.00 (d, J = 1.7 Hz, 9H), -0.10 (d, J = 1.6 Hz, 6H). ^{13}C NMR (101 MHz, $CDCl_3$) δ 210.4, 106.2, 85.2, 62.8, 42.8, 41.0, 32.5, 25.8, 25.4, 23.6, 22.3, 19.1, 18.2, -5.4. HRMS-ESI (m/z): calcd. for $C_{20}H_{40}O_2Si_2H^+$ $[M+H]^+$: 369.2640, found 369.2640.

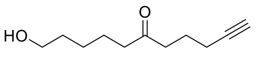

S35

To a solution of **S34** (710 mg, 1.9 mmol) in THF (8.0 mL) was added a solution of TBAF (1.0 M in THF, 4.8 mL, 4.8 mmol) at room temperature. The reaction was stirred at room temperature for 30 min then quenched with H_2O (5.0 mL). The mixture was extracted with EtOAc (3×30 mL). The combined organic phases were washed with brine, dried over Na_2SO_4 and concentrated in vacuo. The residue was purified by silica gel chromatography (EtOAc : hexane = 1:1) gave a yellow oil (350 mg, 100%). ^{1}H NMR (400 MHz, $CDCl_3$) δ 3.64 (td, J = 6.5, 4.4 Hz, 2H), 2.56 (td, J = 7.2, 3.1 Hz, 2H), 2.45 (td, J = 7.2, 3.2 Hz, 2H), 2.23 (tdd, J = 6.8, 4.2, 2.5 Hz, 2H), 1.97 (q, J = 2.6 Hz, 1H), 1.86 – 1.71 (m, 2H), 1.68 – 1.51 (m, 4H), 1.44 – 1.30 (m, 2H). ^{13}C NMR (101 MHz, $CDCl_3$) δ 210.6, 83.6, 69.1, 62.6, 42.8, 41.1, 32.4, 25.4, 23.5, 22.2, 17.8. HRMS-ESI (m/z): calcd. for $C_{11}H_{18}O_2Na^+$ $[M+Na]^+$: 205.1199, found 205.1202

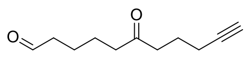

S36

Dess-Martin reagent (413 mg, 0.97 mmol) was added to the solution of **S35** (100 mg, 0.65 mmol) in DCM (6.5 mL) at 0°C, and the mixture was stirred at room temperature for 4 hours. The reaction

mixture was quenched with saturated $Na_2S_2O_3$: $NaHCO_3$ = 1:1 (10 mL), and the product was extracted with DCM (3×20 mL). The organic layer was washed with brine and dried over Na_2SO_4, concentrated in vacuo. The residue was purified by silica gel chromatography (EtOAc : hexane = 1:3) gave a yellow oil (70 mg, 70%). ^{1}H NMR (400 MHz, $CDCl_3$) δ 9.77 (s, 1H), 2.56 (t, 2H), 2.48 – 2.41 (m, 4H), 2.23 (tdd, *J* = 6.9, 2.7, 1.2 Hz, 2H), 1.97 (dq, *J* = 2.7, 1.3 Hz, 1H), 1.79 (d, 2H), 1.70 – 1.55 (m, 4H). ^{13}C NMR (101 MHz, $CDCl_3$) δ 209.8, 202.2, 83.5, 77.4, 77.1, 76.8, 69.1, 43.7, 42.5, 41.1, 23.2, 22.2, 21.6, 17.7. HRMS-ESI (m/z): calcd. for $C_{11}H_{16}O_2H^+$ $[M+H]^+$: 181.1213, found 181.1218

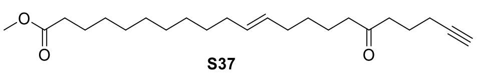

S37

To a stirred solution of the **S4** (174 mg, 0.43 mmol) in DME (2.0 mL) at -78°C under nitrogen was added dropwise the KHMDS (0.5 M in toluene, 0.85 mL, 0.43 mmol) The mixture was then stirred for 30 min before addition of the aldehyde **S36** (70 mg, 0.39 mmol). After stirring for a further 1 h at -78°C the reaction mixture was quenched with sat. NH_4Cl (3.0 mL), then the mixture was extracted with EtOAc (3×10 mL). The combined organic phases were washed with brine, dried over Na_2SO_4 and evaporated. Purification by silica gel column chromatography (EtOAc: hexane = 1:10) gave a yellow oil (65 mg, 46%). ^{1}H NMR (400 MHz, $CDCl_3$) δ 5.46 – 5.30 (m, 2H), 3.66 (s, 3H), 2.55 (t, *J* = 7.2 Hz, 2H), 2.41 (t, *J* = 7.4 Hz, 2H), 2.30 (t, *J* = 7.5 Hz, 2H), 2.23 (td, *J* = 6.9, 2.6 Hz, 2H), 2.08 – 1.91 (m, 5H), 1.79 (d, *J* = 6.4 Hz, 2H), 1.69 – 1.52 (m, 4H), 1.40 – 1.24 (m, 15H). ^{13}C NMR (101 MHz, $CDCl_3$) δ 210.5, 174.3, 130.9, 129.6, 83.6, 69.0, 51.4, 42.8, 41.0, 34.1, 32.6, 32.3, 29.6, 29.4, 29.4, 29.3, 29.2, 29.2, 25.0, 23.4, 22.3, 17.8. HRMS-ESI (m/z): calcd. for $C_{23}H_{38}O_3H^+$ $[M+H]^+$: 363.2894, found 363.2890

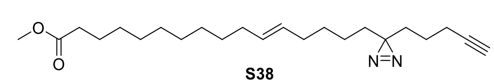

S38

To a solution of **S37** (65 mg, 0.18 mmol) in MeOH (0.10 mL) was added an ammonia solution in MeOH (7 N, 2.0 mL) at 0 °C under Ar. The solution was stirred at that temperature for 1 h then warmed to room temperature stirred for 2 h. Cooled to 0 °C again, to this solution was then added hydroxylamine-O-sulfonic acid (24.3 mg, 0.22 mmol) slowly at 0 °C. The resulting mixture was warmed to rt and stirred for 16 h. The white precipitate was removed by filtration and the remaining solution was concentrated by vacuo. The residue was re-dissolved in DCM (1.0 mL). To this solution was added Et_3N (0.043 mL, 0.3 mmol) and a solution of I_2 (82 mg, 0.32 mmol) in DCM (1.0 mL) dropwise until the solution stayed red-brown. The reaction mixture was quenched by saturated $Na_2S_2O_3$, and extracted with DCM (3×10 mL). The combined organic phases were washed with brine, dried over Na_2SO_4 and evaporated. Purification by silica gel column chromatography (EtOAc: hexane = 1:30) gave a yellow oil (29 mg, 43%). ^{1}H NMR (400 MHz, $CDCl_3$) δ 5.46 – 5.27 (m, 2H), 3.66 (s, 3H), 2.30 (t, *J* = 7.7 Hz, 3H), 2.16 (tdd, *J* = 6.8, 2.6, 1.0 Hz, 2H), 2.02 – 1.89 (m, 5H), 1.67 – 1.56 (m, 2H), 1.56 – 1.44 (m, 2H), 1.41 – 1.20 (m, 18H), 1.15 – 1.03 (m, 1H). ^{13}C NMR (101 MHz, $CDCl_3$) δ 174.3, 130.9, 129.6, 83.4, 68.9, 51.4, 34.1, 32.7, 32.6, 32.3, 31.8, 29.6, 29.4, 29.3, 29.2, 29.1, 29.1, 28.4, 25.0, 23.3, 22.8, 18.0. HRMS-mixed (m/z): calcd. for $C_{23}H_{38}N_2O_2H^+$ $[M+H]^+$: 375.3006, found 375.3018.

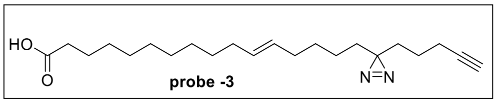

The above product **S38** (also named as control probe N-1 for Fig. 4e,, 28 mg, 0.076mmol) was dissolved in THF:H_2O=3:1 (2.4 mL), then LiOH (13 mg, 0.30 mmol) was added into the above solution, heated the mixture to 66°C and stirred for 3 h, cooled to room temperature, 2M HCl was added to the mixture to adjust the pH to 2, then the mixture was extracted with EtOAc (3×10 mL). The combined organic phases were washed with brine, dried over Na_2SO_4 and evaporated. Purification by silica gel column chromatography (EtOAc : hexane = 1:6) gave a colorless oil (24 mg, 96%).^{1}H NMR (400 MHz, $CDCl_3$) δ 5.47 – 5.23 (m, 2H), 2.35 (t, *J* = 7.5 Hz, 3H), 2.20 – 2.06 (m, 2H), 1.98 – 1.87 (m, 5H), 1.69 – 1.57 (m, 2H), 1.57 – 1.43 (m, 2H), 1.41 – 1.23 (m, 18H), 1.15 – 1.05 (m, 1H). ^{13}C NMR (101 MHz, $CDCl_3$) δ 180.1, 130.9, 129.6, 83.5, 68.9, 34.1, 32.7, 32.6, 32.3, 31.8, 29.6, 29.4, 29.4, 29.2, 29.1, 29.1, 29.1, 28.4, 24.7, 23.3, 22.8, 18.0. HRMS-ESI (m/z): calcd. for $C_{22}H_{36}N_2O_2H^+$ $[M+H]^+$: 361.2850, found 361.2847

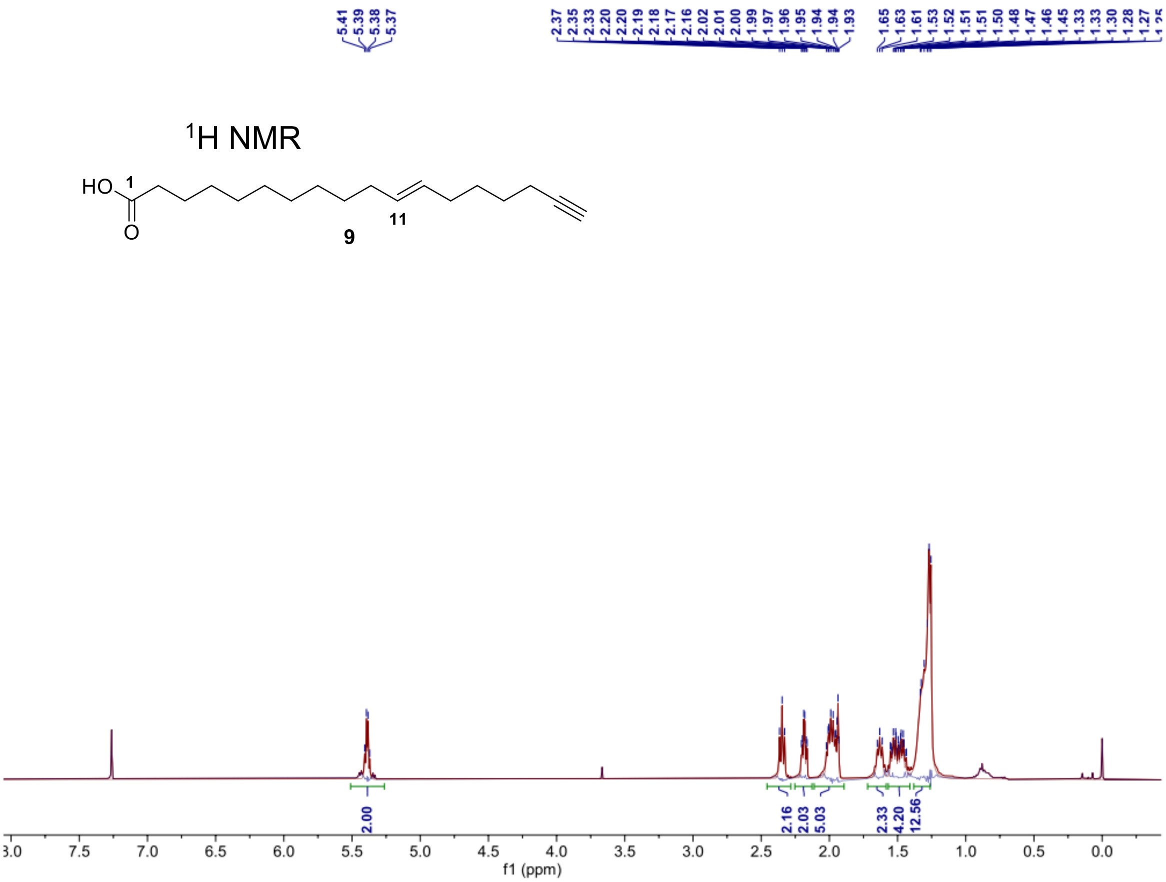
1H NMR
HO
1
O
9
11
2.00
2.16
2.03
5.03
2.33
4.20
12.56
f1 (ppm)

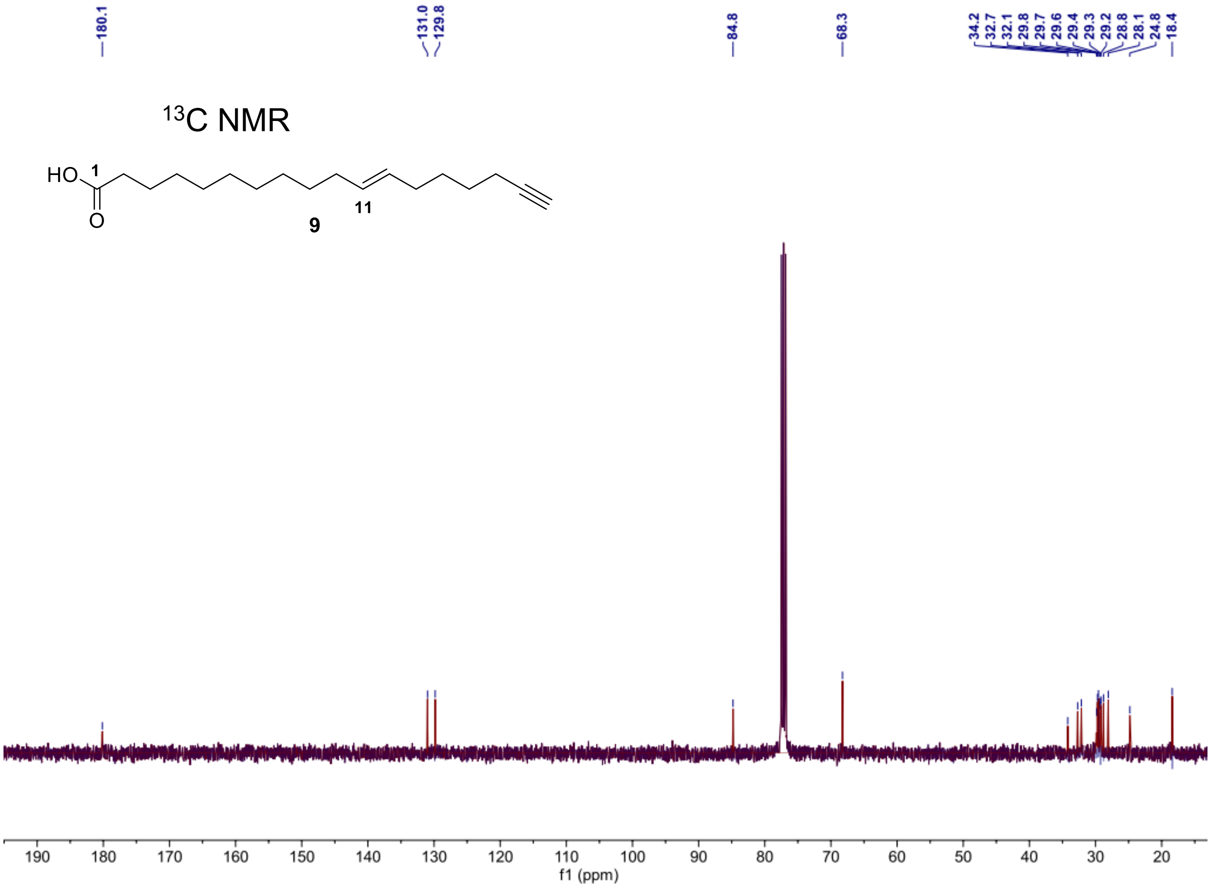

180.1
131.0
129.8
84.8
68.3
34.2
32.7
32.1
29.8
29.7
29.6
29.4
29.3
29.2
28.8
28.1
24.8
18.4
13C NMR
HO
1
O
9
11
190 180 170 160 150 140 130 120 110 100 90 80 70 60 50 40 30 20
f1 (ppm)

^{1}H NMR

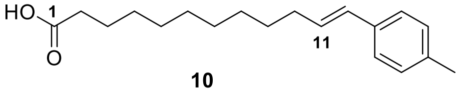

10

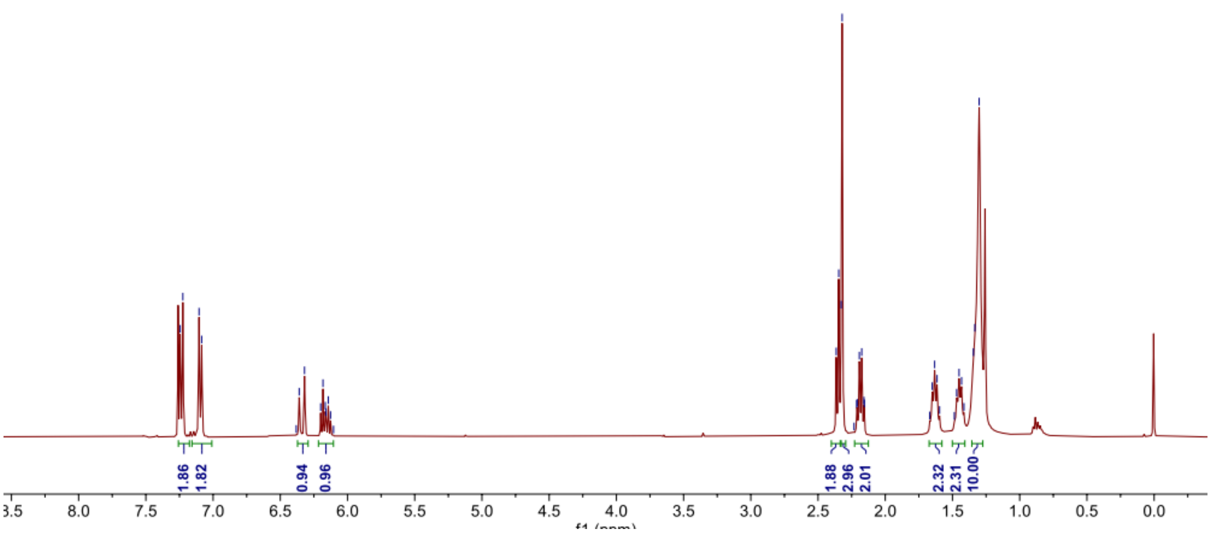

^{13}C NMR

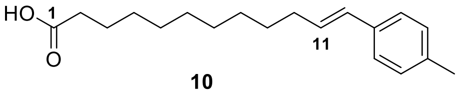

10

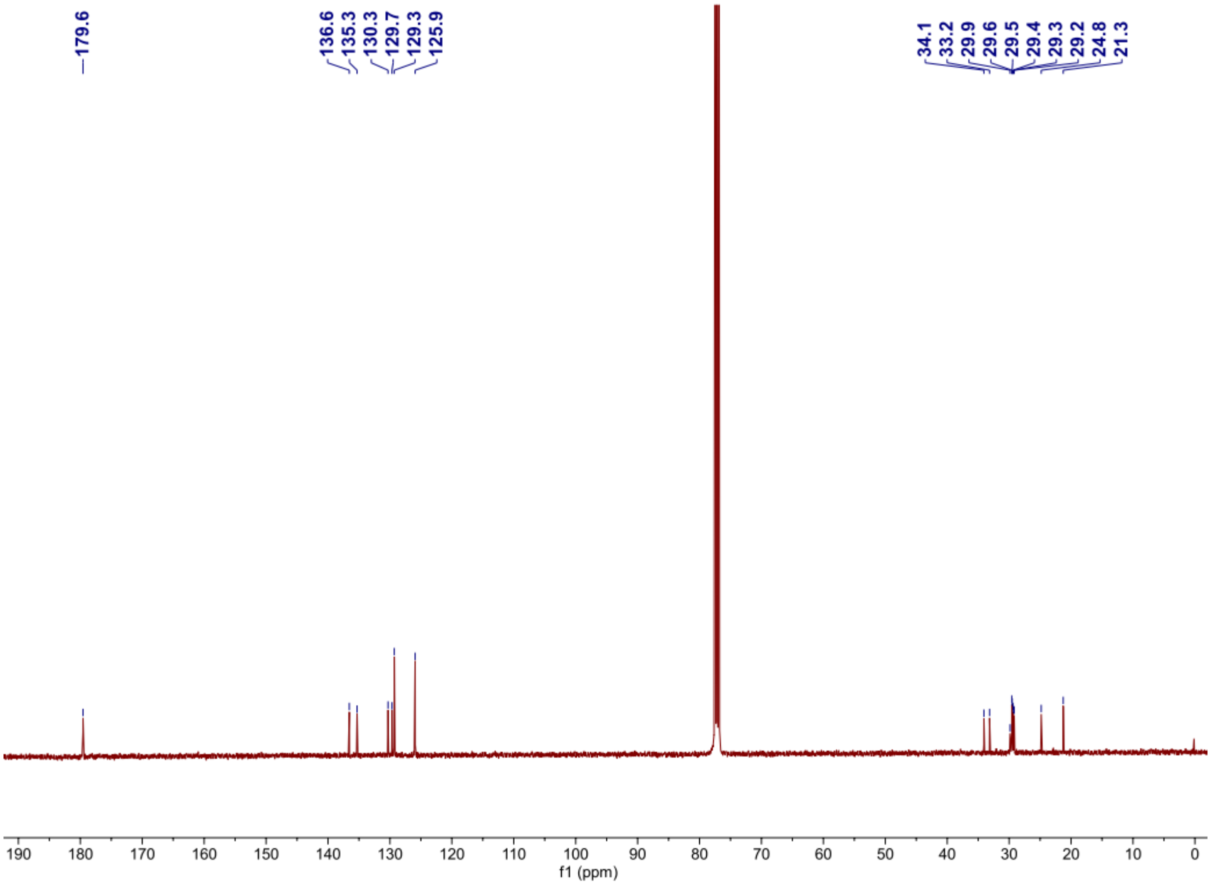
179.6
136.6
135.3
130.3
129.7
129.3
125.9
34.1
33.2
29.9
29.6
29.5
29.4
29.3
29.2
24.8
21.3
190 180 170 160 150 140 130 120 110 100 90 80 70 60 50 40 30 20 10 0
f1 (ppm)

483

^{1}H NMR

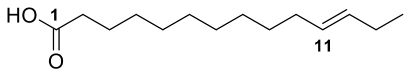

11

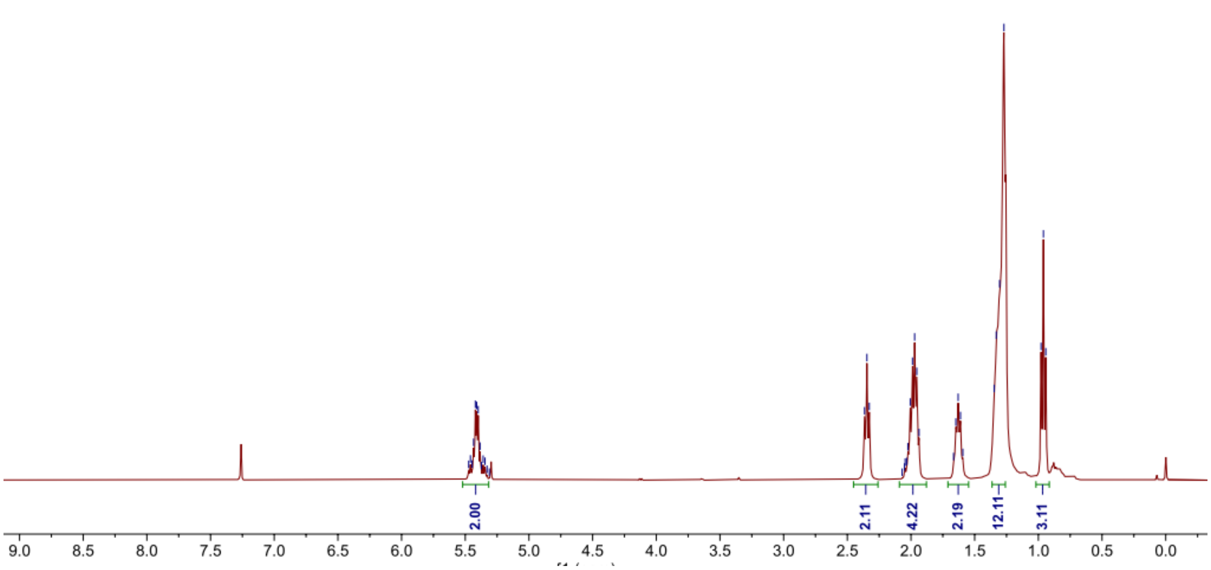

^{13}C

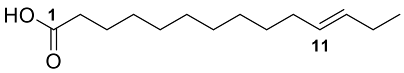

11

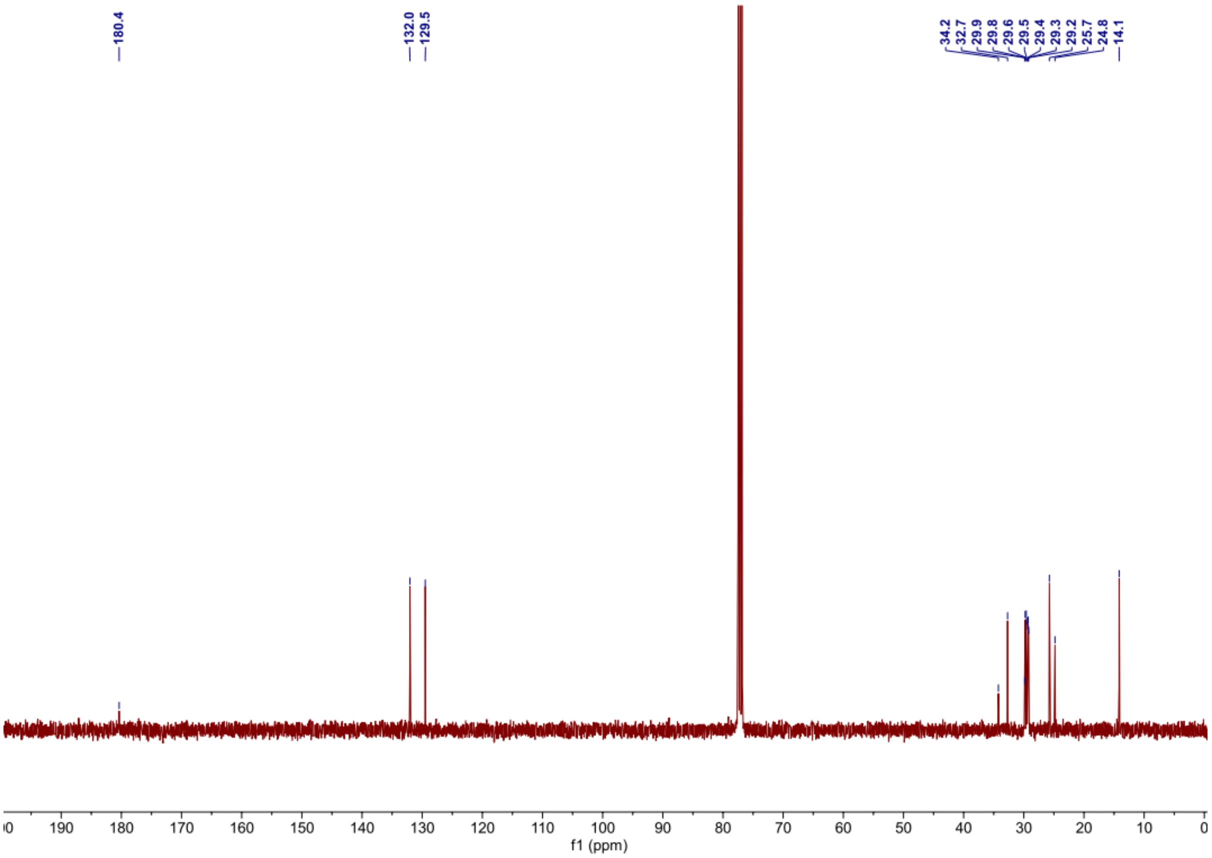
180.4
132.0
129.5
34.2
32.7
29.9
29.8
29.6
29.5
29.4
29.3
29.2
25.7
24.8
14.1
190 180 170 160 150 140 130 120 110 100 90 80 70 60 50 40 30 20 10 0
f1 (ppm)

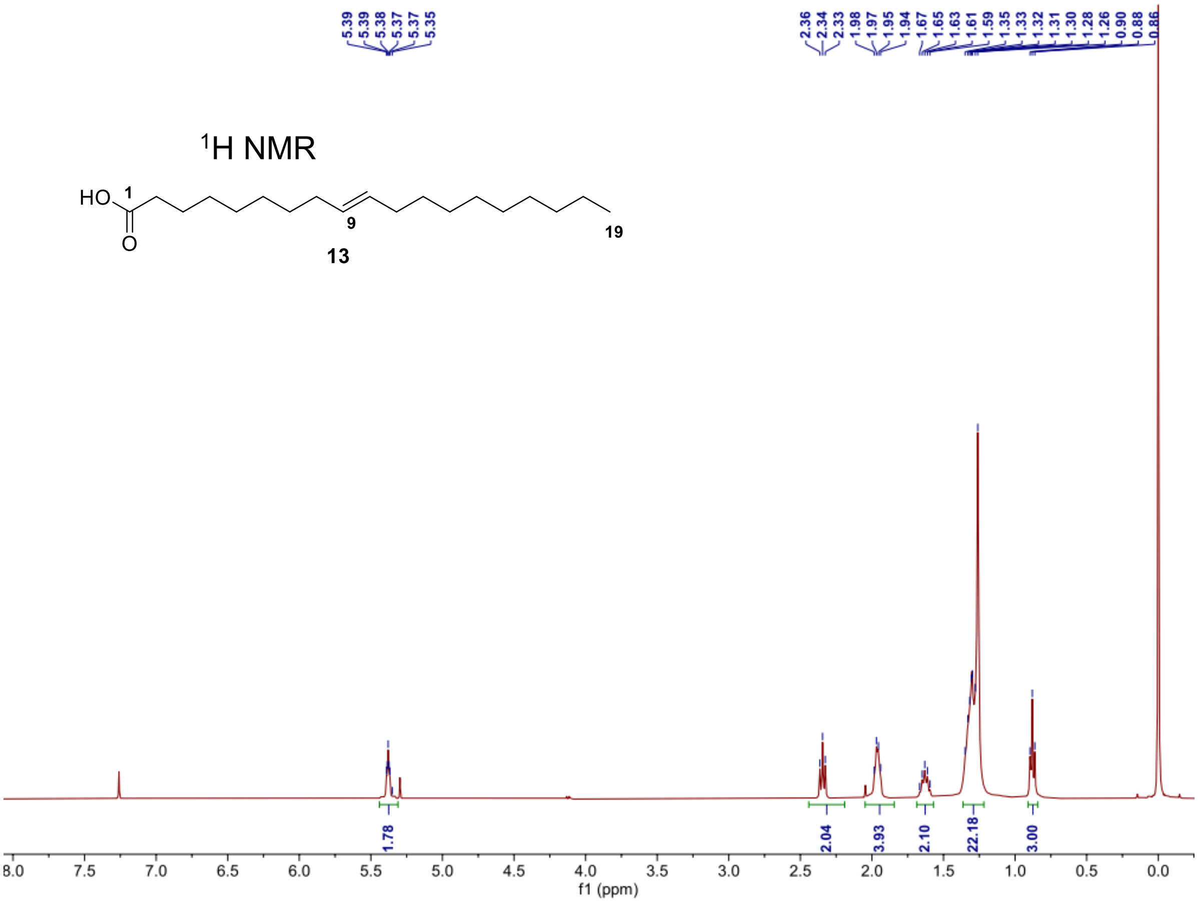

^{13}C

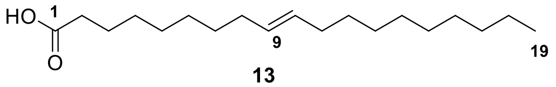

13

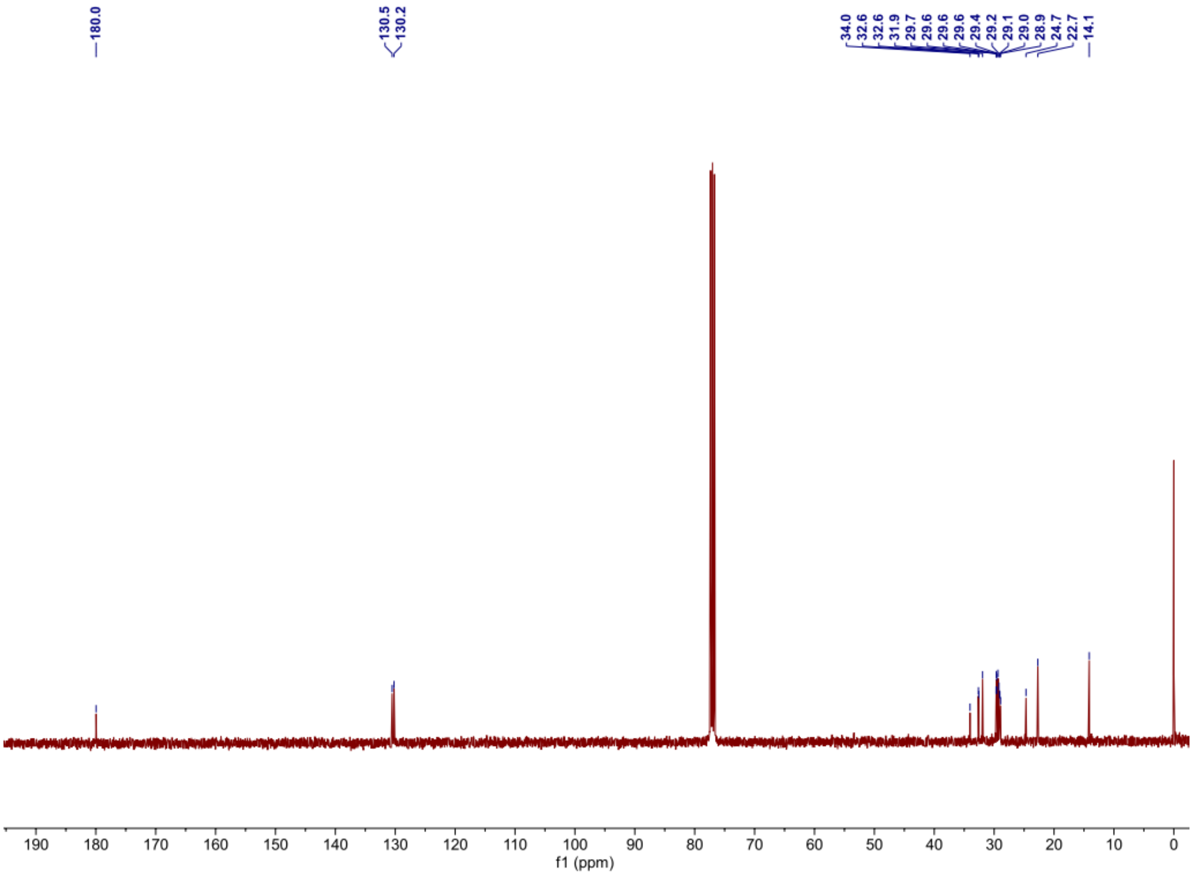
180.0
130.5
130.2
34.0
32.6
32.6
31.9
29.7
29.6
29.6
29.6
29.4
29.2
29.1
29.0
28.9
24.7
22.7
14.1
190 180 170 160 150 140 130 120 110 100 90 80 70 60 50 40 30 20 10 0
f1 (ppm)

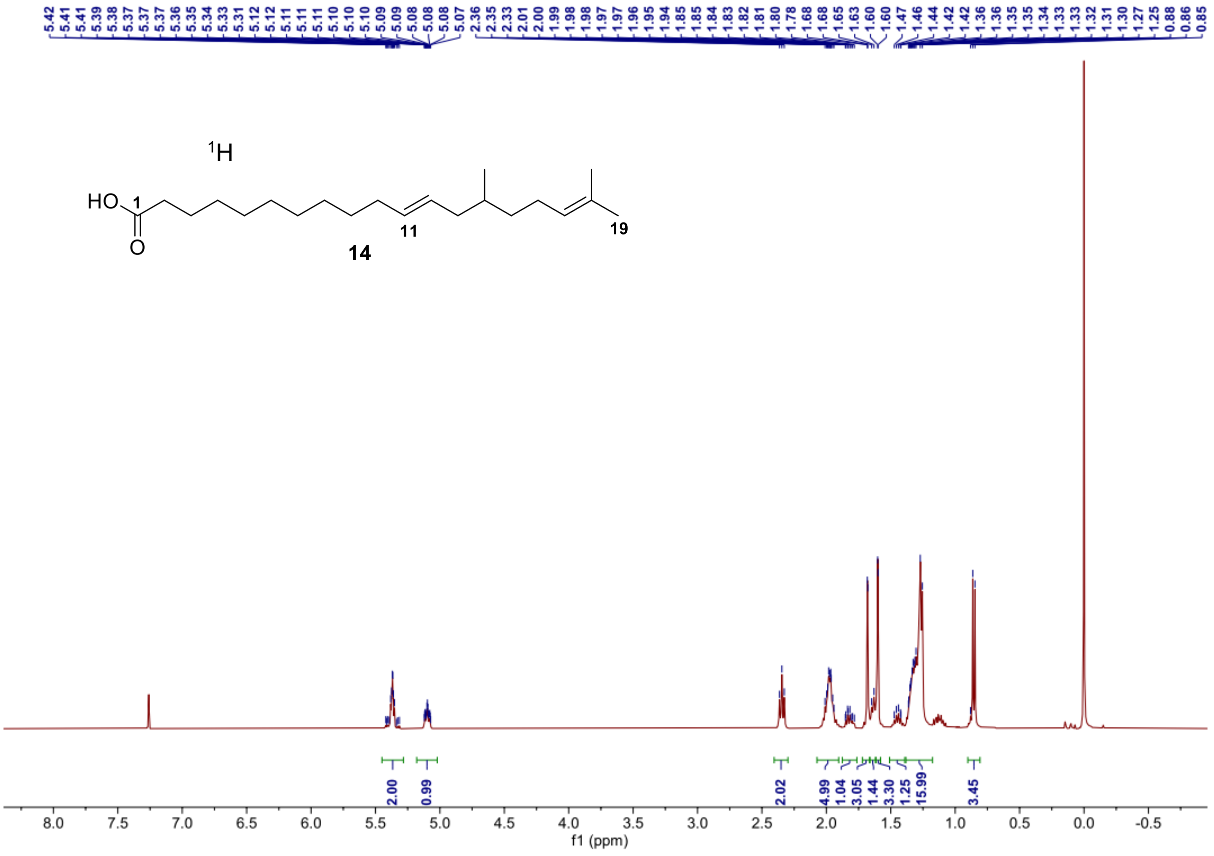

5.42 5.41 5.39 5.38 5.37 5.37 5.36 5.35 5.34 5.33 5.31 5.12 5.12 5.11 5.11 5.10 5.10 5.09 5.09 5.08 5.08 5.08 5.07 2.36 2.35 2.33 2.01 2.00 1.99 1.98 1.98 1.97 1.96 1.95 1.94 1.85 1.85 1.84 1.83 1.82 1.81 1.80 1.78 1.68 1.68 1.65 1.63 1.60 1.60 1.60 1.47 1.46 1.44 1.42 1.42 1.36 1.36 1.35 1.34 1.33 1.33 1.32 1.31 1.30 1.27 1.25 0.88 0.86 0.85
^{1}H
HO
1
O
11
19
14
2.00
0.99
2.02
4.99
1.04
3.05
1.44
3.30
1.25
15.99
3.45
8.0 7.5 7.0 6.5 6.0 5.5 5.0 4.5 4.0 3.5 3.0 2.5 2.0 1.5 1.0 0.5 0.0 -0.5
f1 (ppm)

489

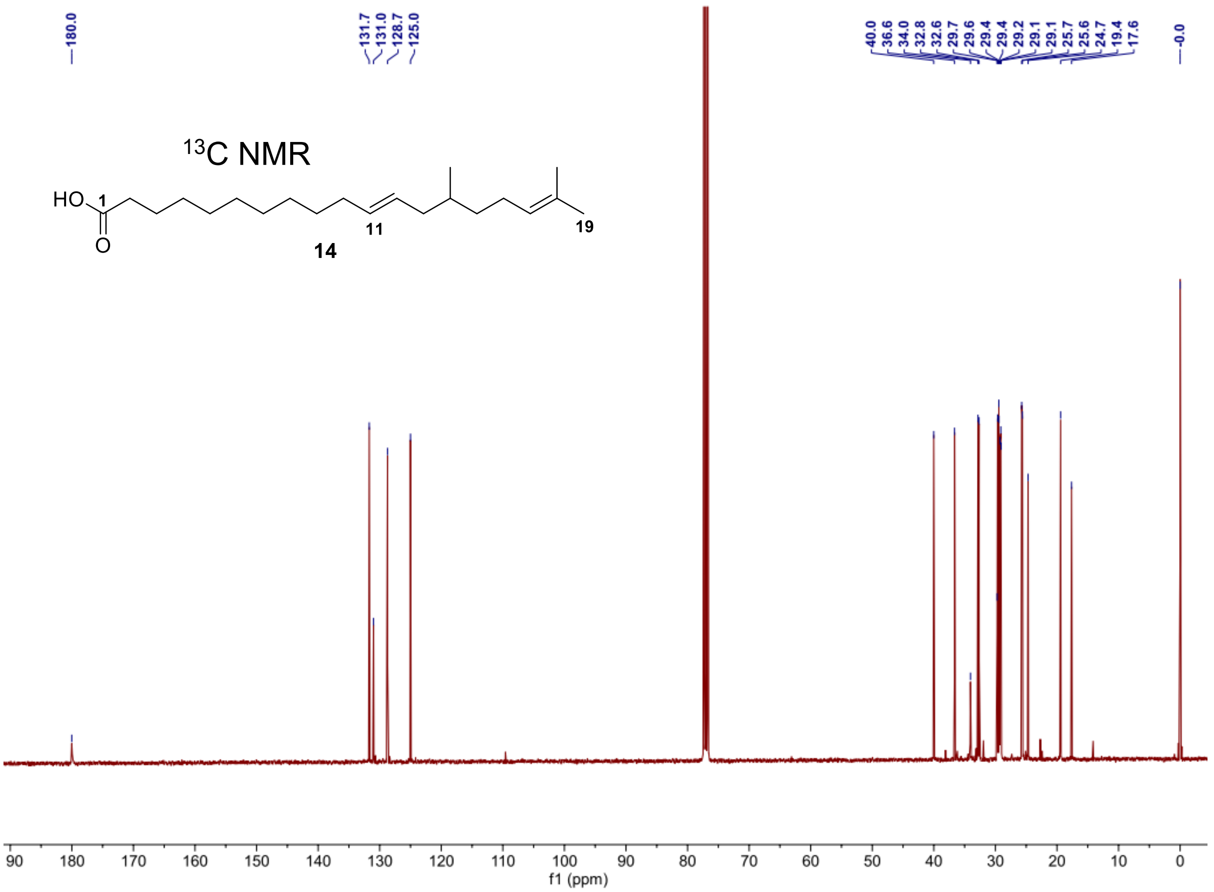
180.0
131.7
131.0
128.7
125.0
40.0
36.6
34.0
32.8
32.6
29.7
29.6
29.4
29.4
29.2
29.1
29.1
25.7
25.6
24.7
19.4
17.6
-0.0
13C NMR
HO
1
O
11
19
14
90 180 170 160 150 140 130 120 110 100 90 80 70 60 50 40 30 20 10 0
f1 (ppm)

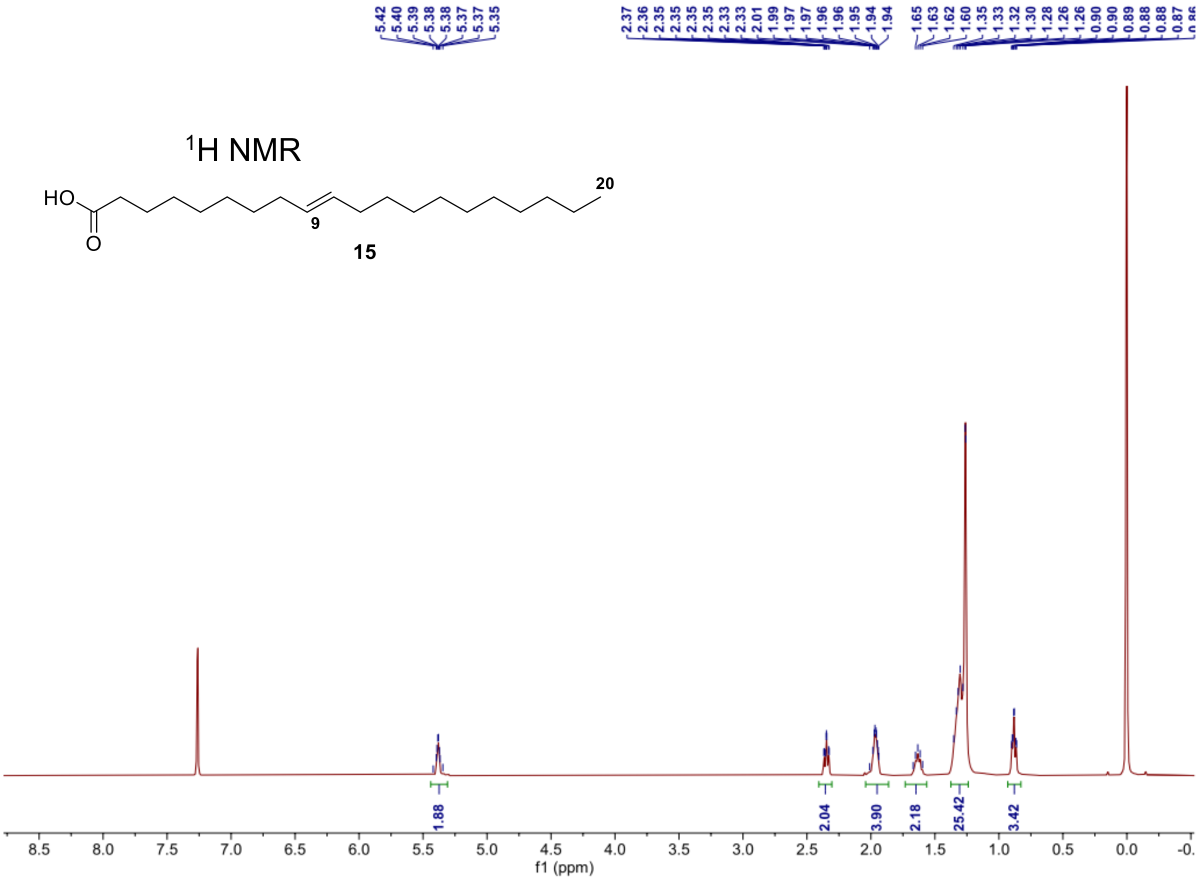

1H NMR
HO
O
9
20
15
5.42
5.40
5.39
5.38
5.38
5.37
5.35
2.37
2.36
2.35
2.35
2.35
2.35
2.33
2.33
2.01
1.99
1.97
1.97
1.96
1.96
1.95
1.94
1.94
1.65
1.63
1.62
1.60
1.35
1.33
1.32
1.30
1.28
1.26
1.26
0.90
0.90
0.89
0.88
0.88
0.87
1.88
2.04
3.90
2.18
25.42
3.42
8.5
8.0
7.5
7.0
6.5
6.0
5.5
5.0
4.5
4.0
3.5
3.0
2.5
2.0
1.5
1.0
0.5
0.0
f1 (ppm)

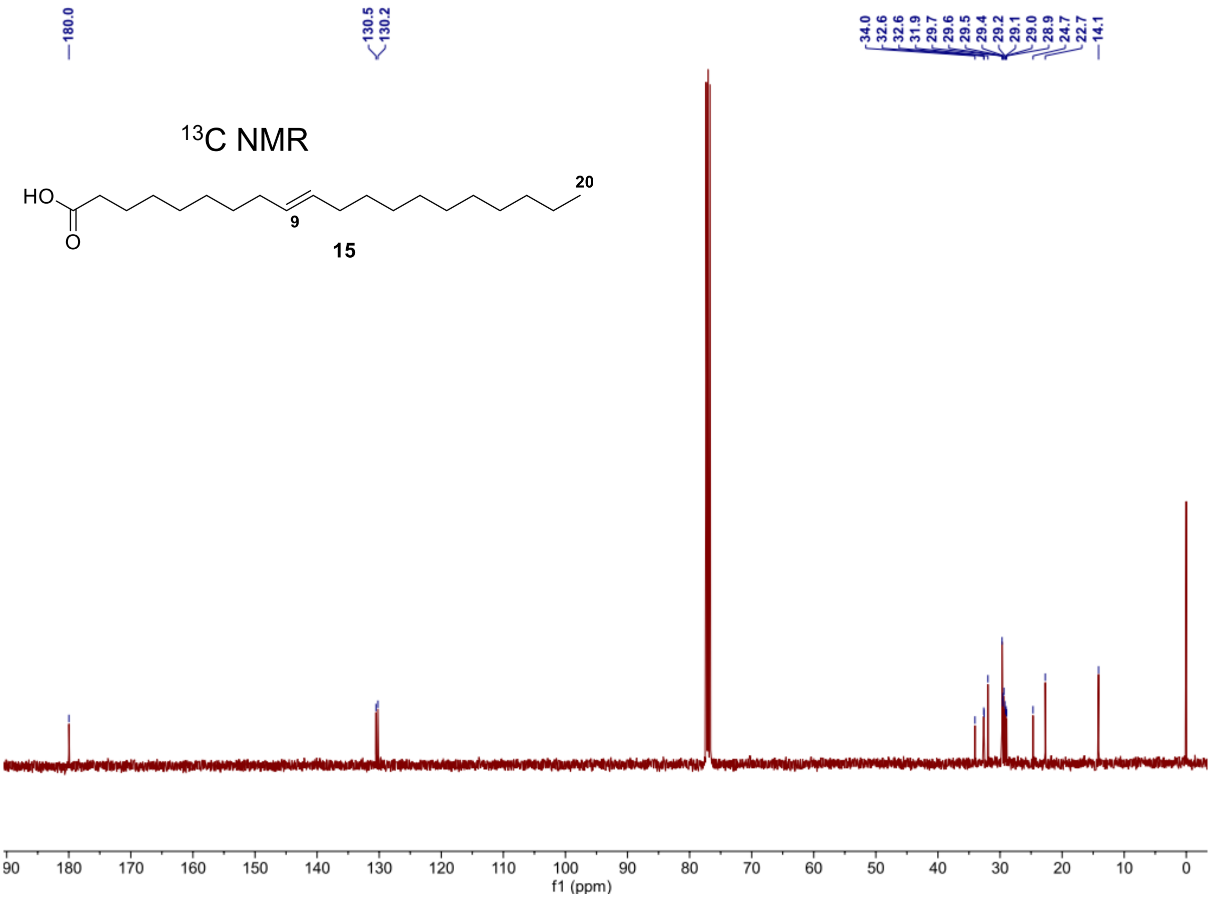
180.0
130.5
130.2
34.0
32.6
32.6
31.9
29.7
29.6
29.5
29.4
29.2
29.1
29.0
28.9
24.7
22.7
14.1
^{13}C NMR
HO
O
9
20
15
90 180 170 160 150 140 130 120 110 100 90 80 70 60 50 40 30 20 10 0
f1 (ppm)

^{1}H NMR

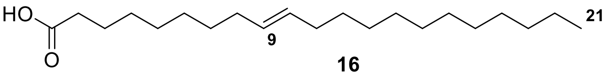

16

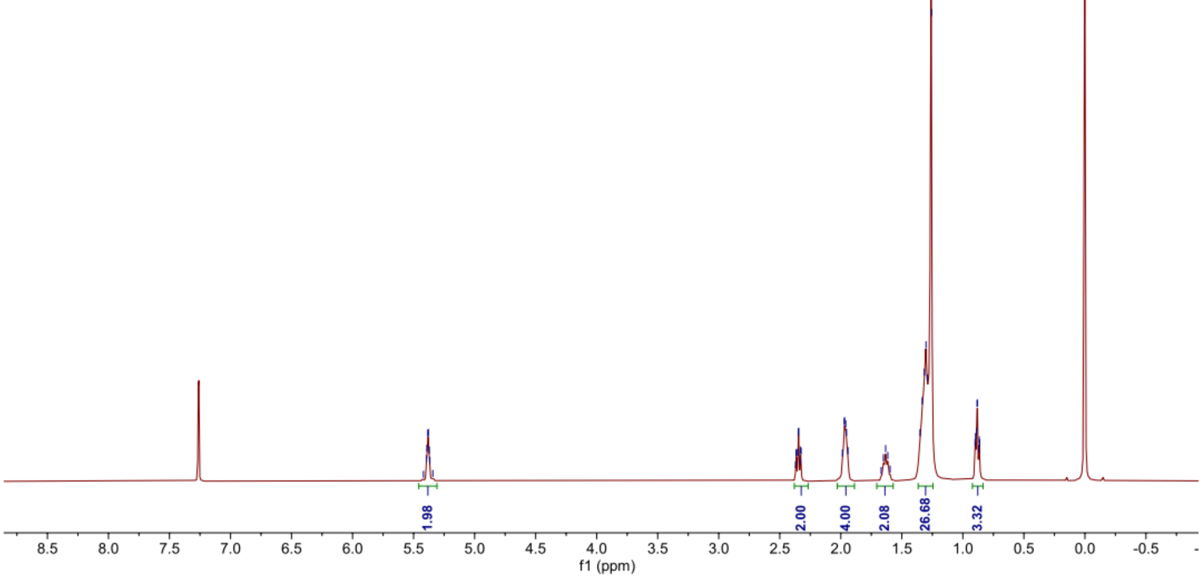

^{13}C NMR

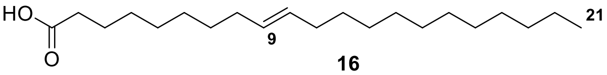

16

494

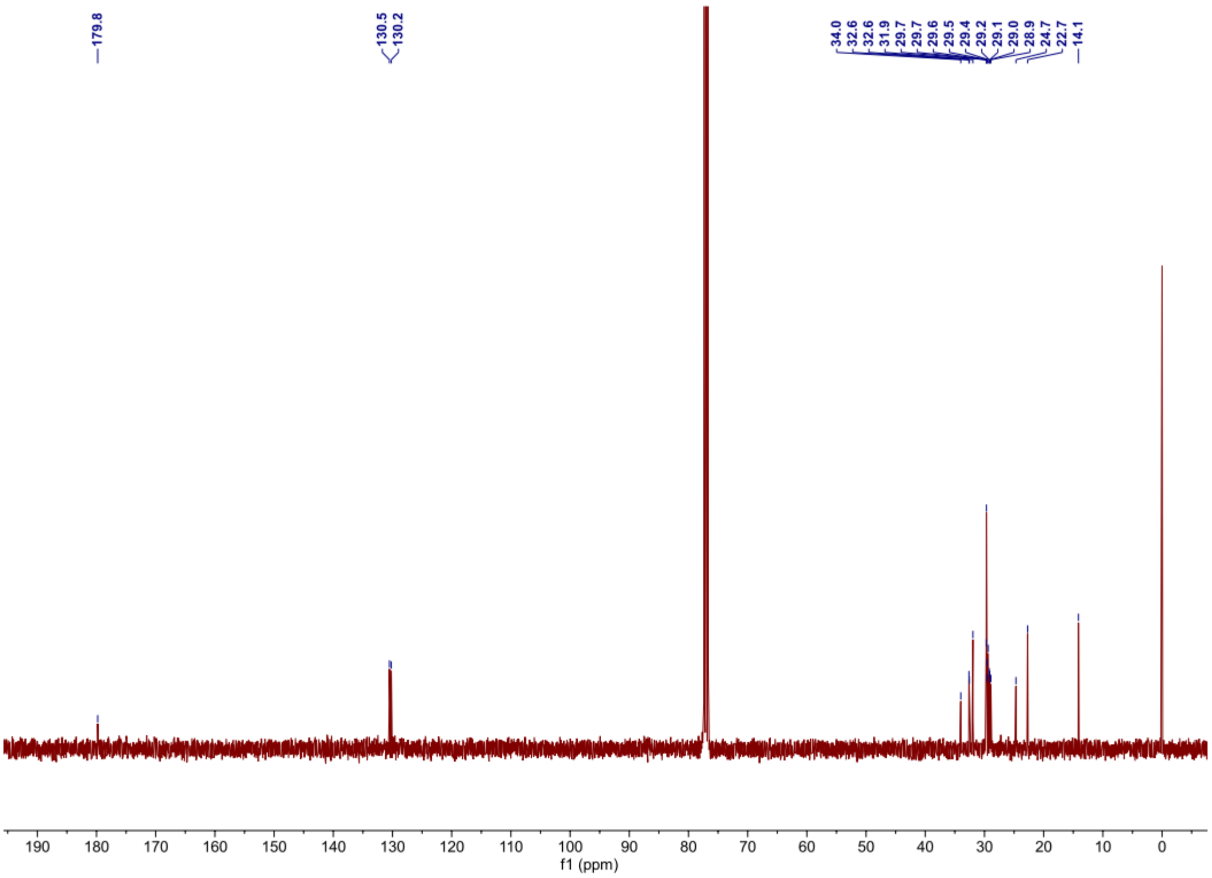

179.8
130.5
130.2
34.0
32.6
32.6
31.9
29.7
29.7
29.6
29.5
29.4
29.2
29.1
29.0
28.9
24.7
22.7
14.1
190
180
170
160
150
140
130
120
110
100
90
80
70
60
50
40
30
20
10
0
f1 (ppm)

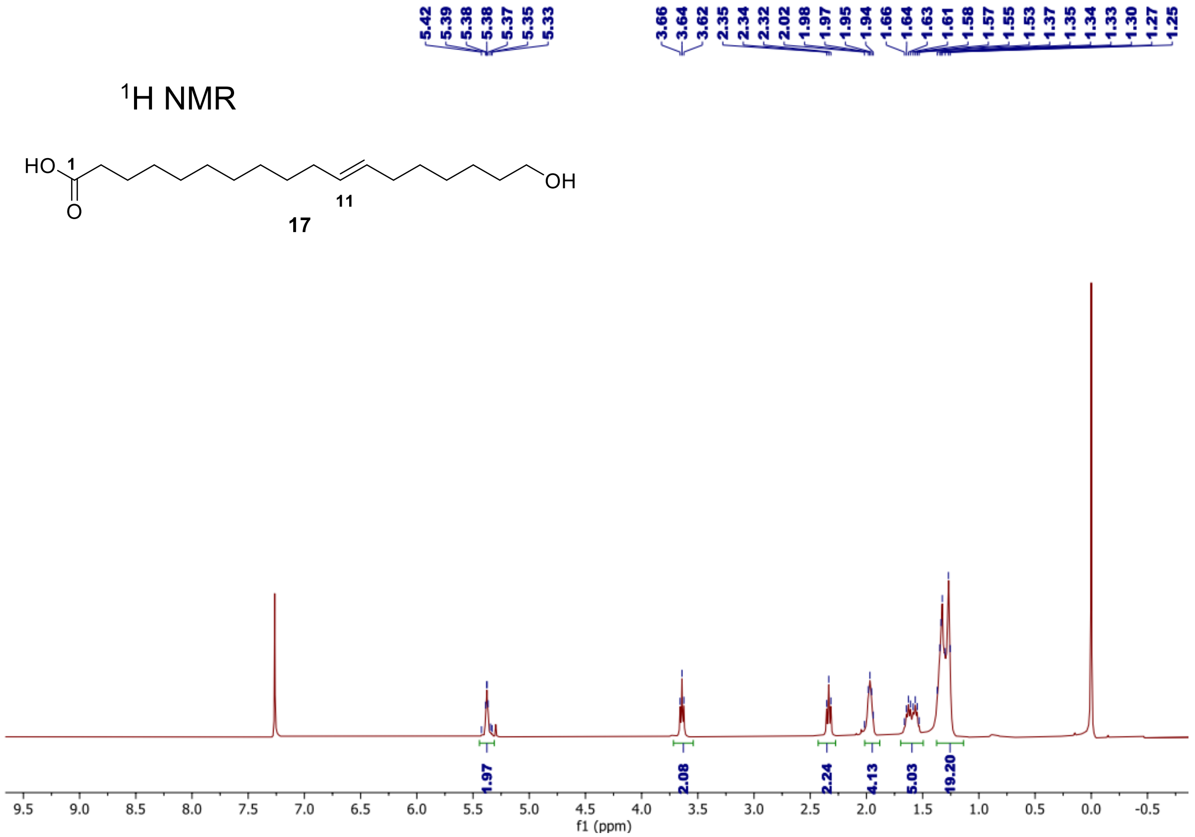

^{1}H NMR
HO
1
O
11
OH
17
5.42 5.39 5.38 5.38 5.37 5.35 5.33
3.66 3.64 3.62 2.35 2.34 2.32 2.02 1.98 1.97 1.95 1.94 1.66 1.64 1.63 1.61 1.58 1.57 1.55 1.53 1.37 1.35 1.34 1.33 1.30 1.27 1.25
1.97 2.08 2.24 4.13 5.03 19.20
9.5 9.0 8.5 8.0 7.5 7.0 6.5 6.0 5.5 5.0 4.5 4.0 3.5 3.0 2.5 2.0 1.5 1.0 0.5 0.0 -0.5
f1 (ppm)

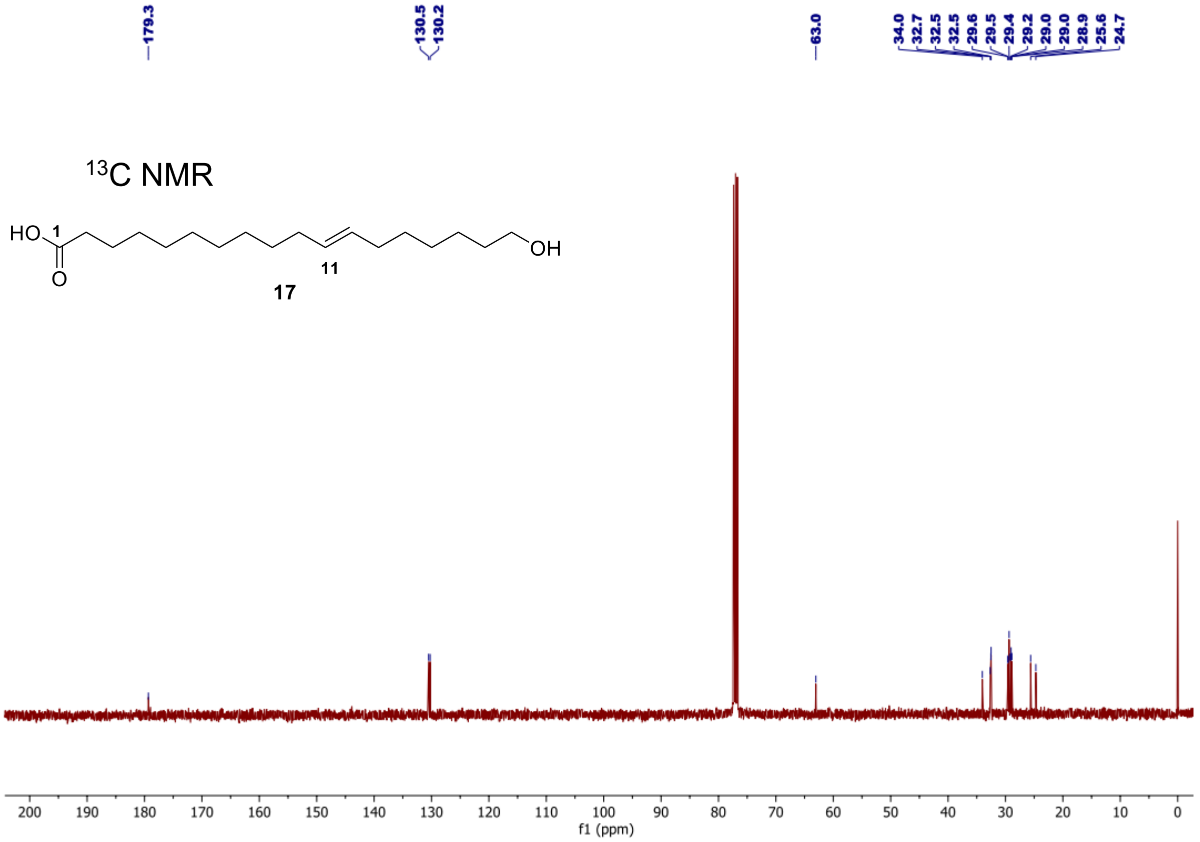

^{13}C NMR
HO
1
O
11
OH
17
179.3
130.5 130.2
63.0
34.0 32.7 32.5 32.5 29.6 29.5 29.4 29.2 29.0 29.0 28.9 25.6 24.7
200 190 180 170 160 150 140 130 120 110 100 90 80 70 60 50 40 30 20 10 0
f1 (ppm)

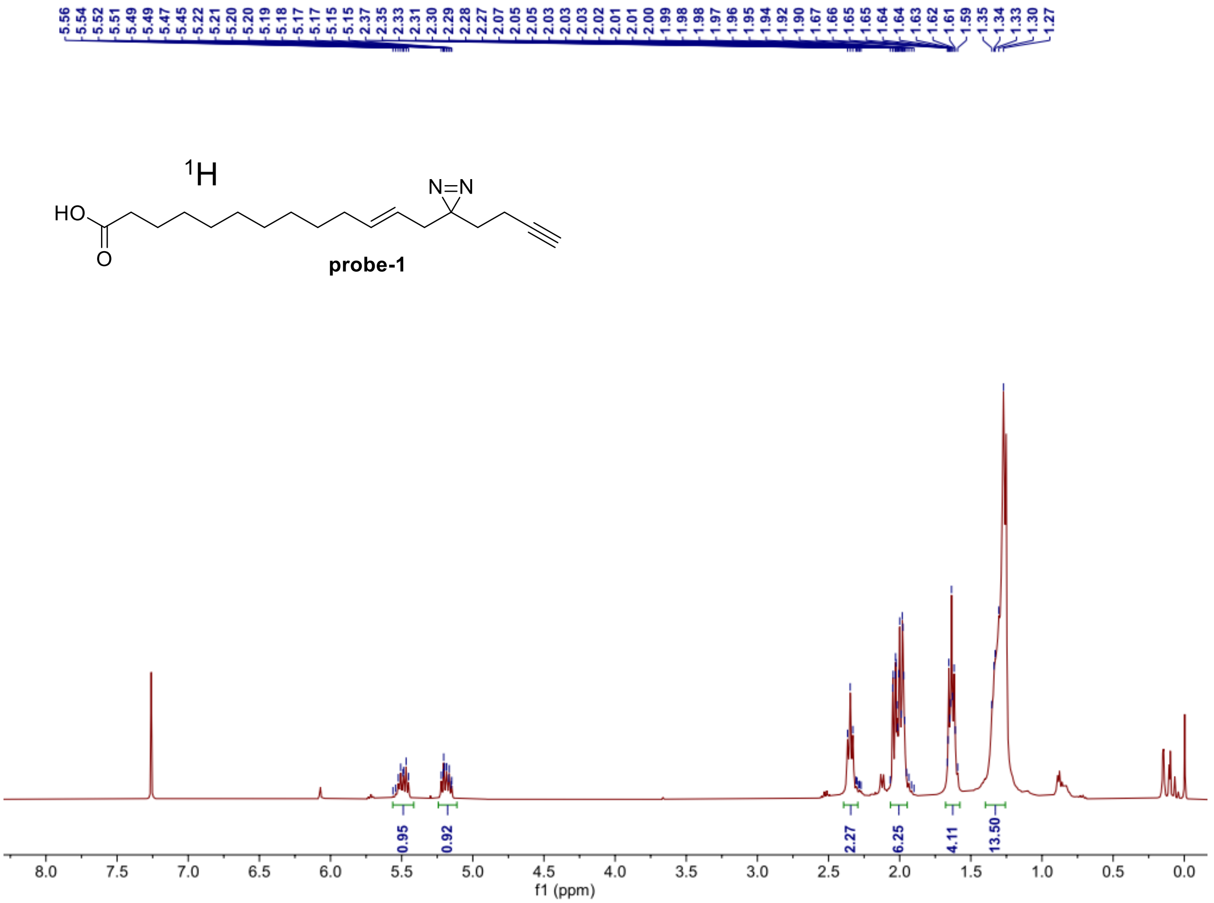

1H
HO
O
N=N
probe-1
0.95
0.92
2.27
6.25
4.11
13.50
8.0 7.5 7.0 6.5 6.0 5.5 5.0 4.5 4.0 3.5 3.0 2.5 2.0 1.5 1.0 0.5 0.0
f1 (ppm)

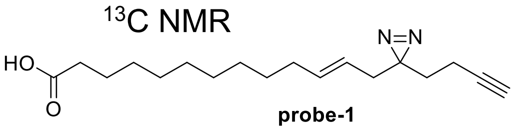

13C NMR
HO
O
N=N
probe-1

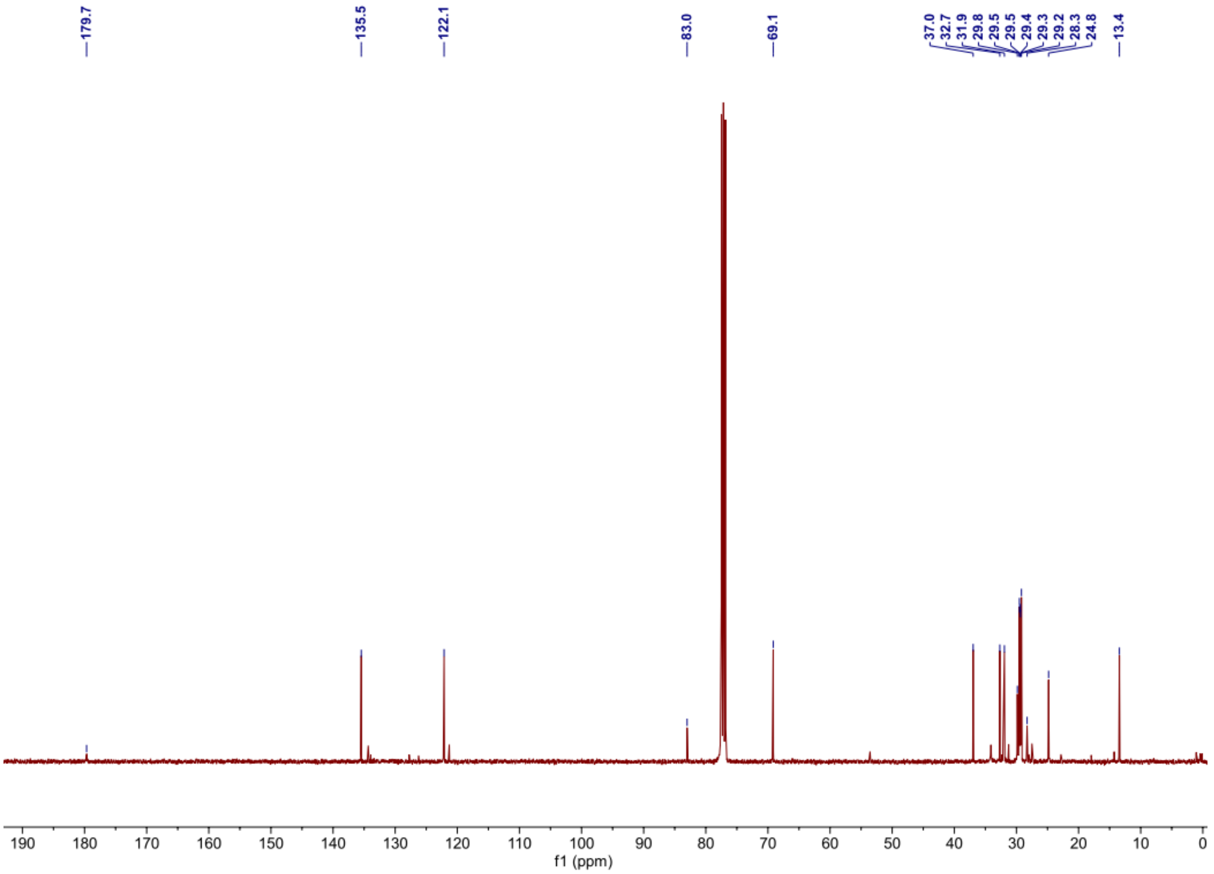
179.7
135.5
122.1
83.0
69.1
37.0
32.7
31.9
29.8
29.5
29.5
29.4
29.3
29.2
28.3
24.8
13.4
190 180 170 160 150 140 130 120 110 100 90 80 70 60 50 40 30 20 10 0
f1 (ppm)

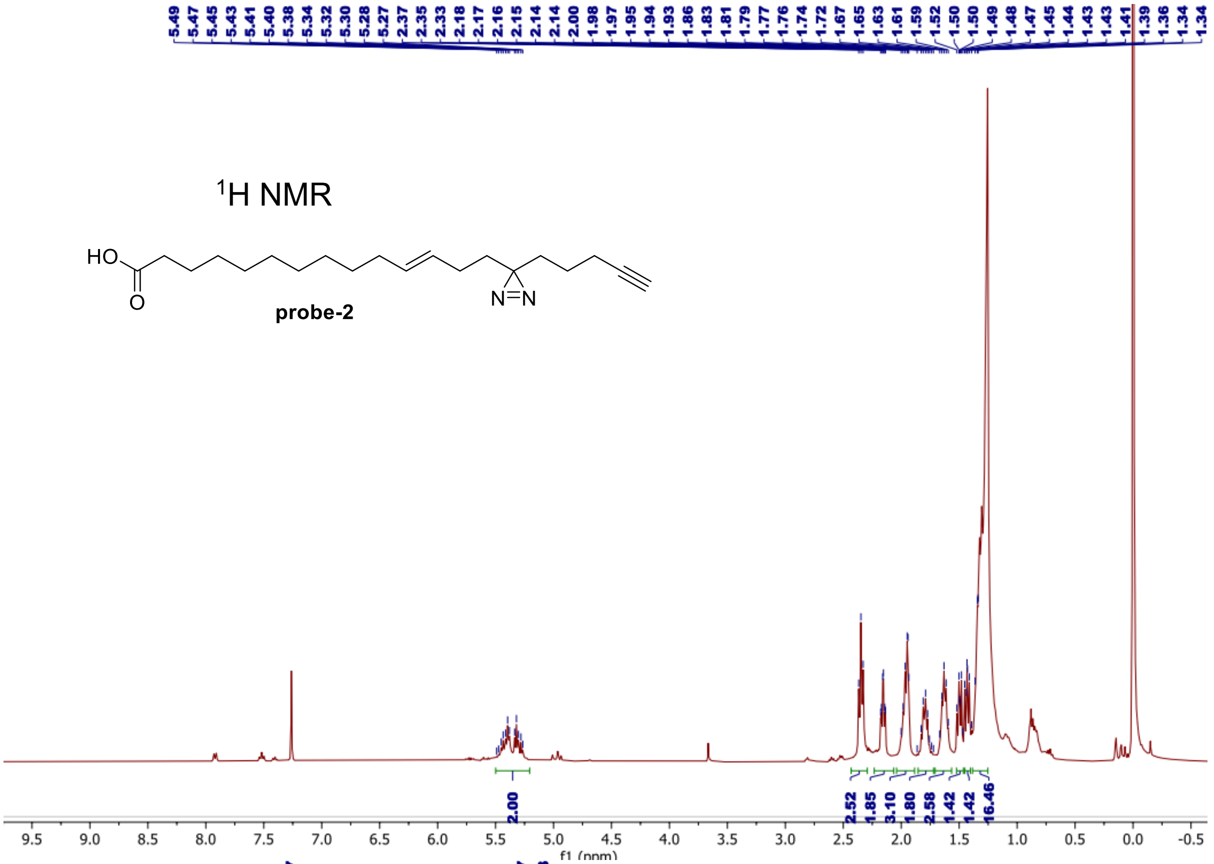

¹H NMR
HO
O
probe-2
N=N
f1 (ppm)

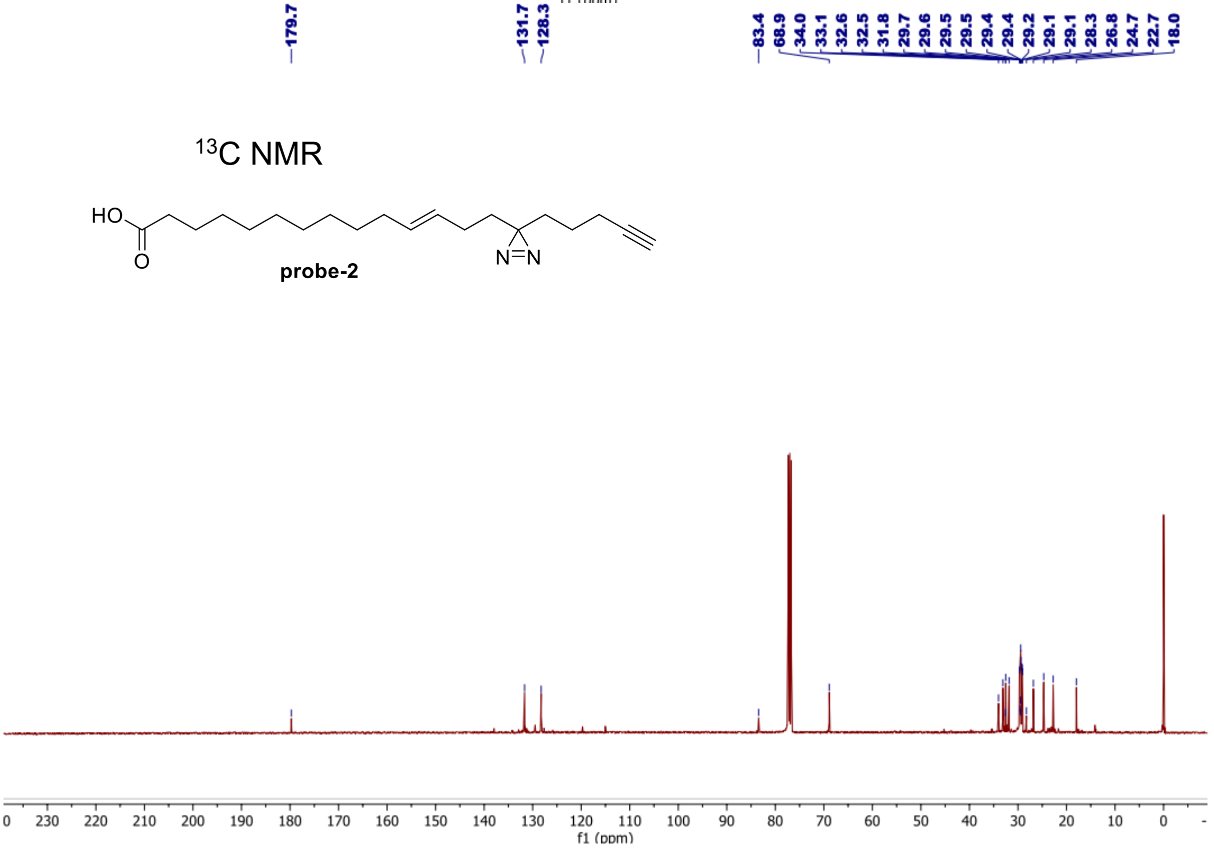

¹³C NMR
HO
O
probe-2
N=N
f1 (ppm)

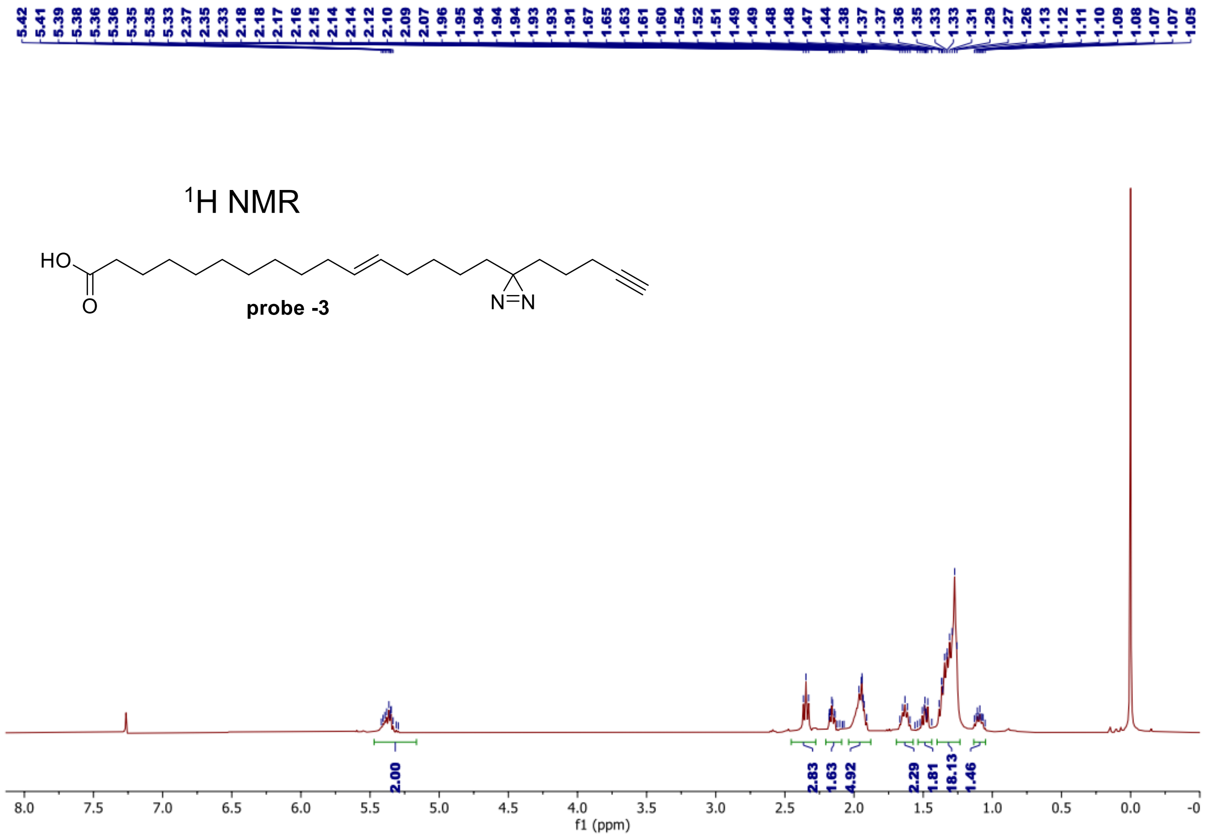
1H NMR
HO
O
probe -3
N=N
2.00
2.83
1.63
4.92
2.29
1.81
18.13
1.46
f1 (ppm)

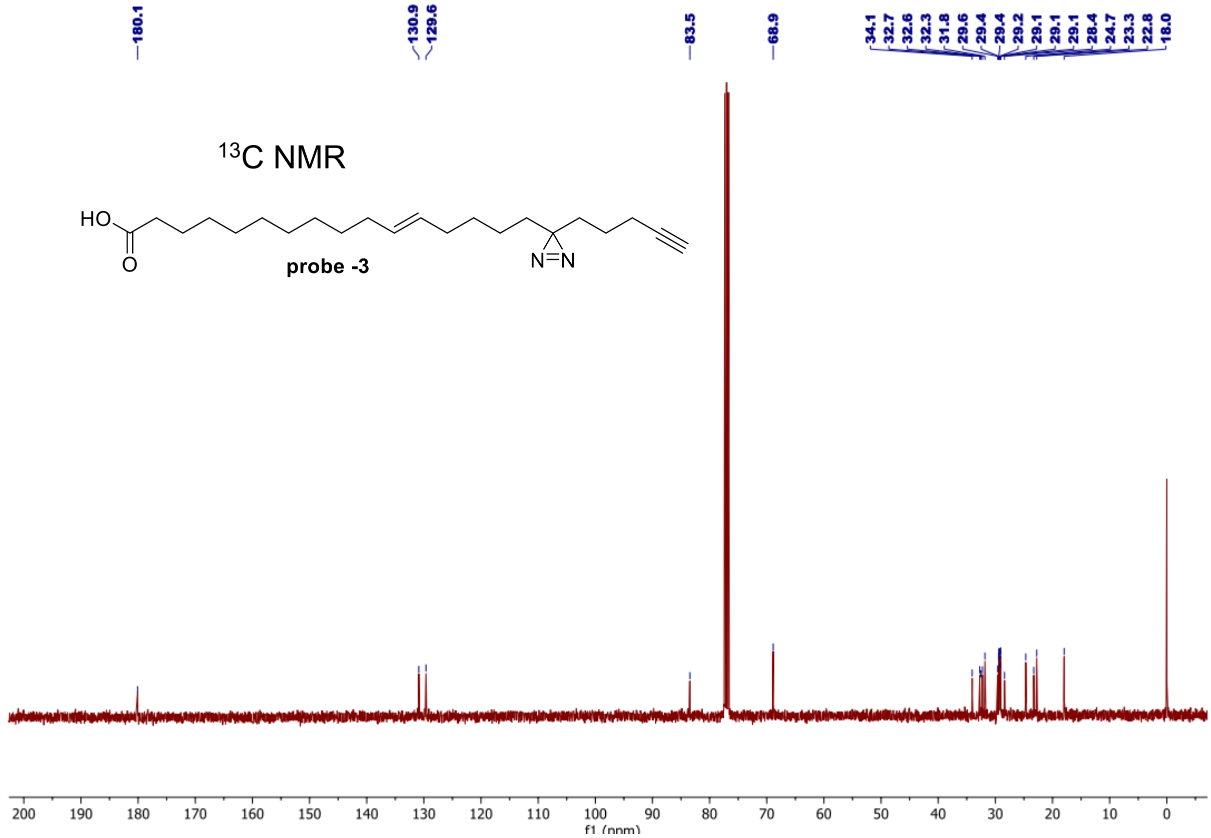
13C NMR
HO
O
probe -3
N=N
180.1
130.9
129.6
83.5
68.9
34.1
32.7
32.6
32.3
31.8
29.6
29.4
29.2
29.1
29.1
29.1
28.4
24.7
23.3
22.8
18.0

1 Jana, S., Sarpe, V. A. & Kulkarni, S. S. Total Synthesis of Emmyguyacins A and B, Potential Fusion Inhibitors of Influenza Virus. *Org Lett* **20**, 6938-6942 (2018). https://doi.org:10.1021/acs.orglett.8b03073

2 W.J. Kerr, A. J. M., and L.C. Paterson. Synthesis of α-methylene propellanone via the strategic employment of metal-mediated cyclisation chemistry. *Tetrahedron* **71**, 5356-5361 (2015). https://doi.org:https://doi.org/10.1016/j.tet.2015.06.003

3 Inui, M., Nakazaki, A. & Kobayashi, S. Highly stereoselective construction of spiro[4.5]decanes by SmI(2)-promoted ketyl radical mediated tandem cyclization. *Org Lett* **9**, 469-472 (2007). https://doi.org:10.1021/ol0628255
