# Supplementary material for: Trans-vaccenic acid reprograms CD8+ T cells and anti-tumour immunity
Source: Nature. 2023 Nov 22;623(7989):1034–43. doi: 10.1038/s41586-023-06749-3 (PMC10686835; doi:10.1038/s41586-023-06749-3)
Supplement: Supplementary file 2 — Reporting Summary [file 41586_2023_6749_MOESM2_ESM.pdf]

nature portfolio

Corresponding author(s): Jing Chen
Chuan He
Hongbo Chi

Last updated by author(s): Sep 27, 2023

# Reporting Summary

Nature Portfolio wishes to improve the reproducibility of the work that we publish. This form provides structure for consistency and transparency in reporting. For further information on Nature Portfolio policies, see our Editorial Policies and the Editorial Policy Checklist.

## Statistics

For all statistical analyses, confirm that the following items are present in the figure legend, table legend, main text, or Methods section.

| n/a | Confirmed | |
|---|---|---|
| ☐ | ☒ | The exact sample size (*n*) for each experimental group/condition, given as a discrete number and unit of measurement |
| ☐ | ☒ | A statement on whether measurements were taken from distinct samples or whether the same sample was measured repeatedly |
| ☐ | ☒ | The statistical test(s) used AND whether they are one- or two-sided<br>*Only common tests should be described solely by name; describe more complex techniques in the Methods section.* |
| ☐ | ☒ | A description of all covariates tested |
| ☐ | ☒ | A description of any assumptions or corrections, such as tests of normality and adjustment for multiple comparisons |
| ☐ | ☒ | A full description of the statistical parameters including central tendency (e.g. means) or other basic estimates (e.g. regression coefficient) AND variation (e.g. standard deviation) or associated estimates of uncertainty (e.g. confidence intervals) |
| ☐ | ☒ | For null hypothesis testing, the test statistic (e.g. *F*, *t*, *r*) with confidence intervals, effect sizes, degrees of freedom and *P* value noted<br>*Give P values as exact values whenever suitable.* |
| ☒ | ☐ | For Bayesian analysis, information on the choice of priors and Markov chain Monte Carlo settings |
| ☒ | ☐ | For hierarchical and complex designs, identification of the appropriate level for tests and full reporting of outcomes |
| ☒ | ☐ | Estimates of effect sizes (e.g. Cohen's *d*, Pearson's *r*), indicating how they were calculated |

*Our web collection on statistics for biologists contains articles on many of the points above.*

## Software and code

Policy information about availability of computer code

| | |
|---|---|
| Data collection | BD LSR-Fortessa oftware (v8) was used to collect flow cytometry data |
| Data analysis | ImageJ (Java 8) for westernblot results;<br>FlowJo 10.4 for FCAS results;<br>GraphPad Prism 9 for statistics;<br>Mass spectrometry analysis: Skyline (v21.2) |

For manuscripts utilizing custom algorithms or software that are central to the research but not yet described in published literature, software must be made available to editors and reviewers. We strongly encourage code deposition in a community repository (e.g. GitHub). See the Nature Portfolio guidelines for submitting code & software for further information.

## Data

Policy information about availability of data

All manuscripts must include a data availability statement. This statement should provide the following information, where applicable:

- Accession codes, unique identifiers, or web links for publicly available datasets
- A description of any restrictions on data availability
- For clinical datasets or third party data, please ensure that the statement adheres to our policy

16S amplicon sequencing data have been deposited in the GEO repository with the accession number GSE202266 and are publicly available as of the date of

publication. The KAS-seq data have been deposited in the GEO repository with the accession number GSE202730. The RNA-sequencing data have been deposited in the GEO repository with the accession number GSE202276 and GSE202274. Project number and accession links are listed in the "Data availability" section. Human metabolome database: https://hmdb.ca/

## Human research participants

Policy information about studies involving human research participants and Sex and Gender in Research.

| | |
|---|---|
| Reporting on sex and gender | N/A |
| Population characteristics | N/A |
| Recruitment | N/A |
| Ethics oversight | N/A |

Note that full information on the approval of the study protocol must also be provided in the manuscript.

# Field-specific reporting

Please select the one below that is the best fit for your research. If you are not sure, read the appropriate sections before making your selection.

☒ Life sciences ☐ Behavioural & social sciences ☐ Ecological, evolutionary & environmental sciences

For a reference copy of the document with all sections, see nature.com/documents/nr-reporting-summary-flat.pdf

# Life sciences study design

All studies must disclose on these points even when the disclosure is negative.

| | |
|---|---|
| Sample size | No statical methods were used to predetermine sample size. The sample size was chosen based on preliminary experiments and previous publications (Bachem et al. Immunity 2019; Chen et al. Nature Immunology 2022) |
| Data exclusions | No data were excluded. |
| Replication | All the experimental findings were reliably reproduced as validated by at least two independent experiments. |
| Randomization | All mice and cells been used were randomly divided into groups. |
| Blinding | The investigators were not blinded to group allocation during data collection or analysis. This approach is considered standard for experiments of the type performed in this study, as genetic background of mice must be predetermined prior to analysis. |

# Reporting for specific materials, systems and methods

We require information from authors about some types of materials, experimental systems and methods used in many studies. Here, indicate whether each material, system or method listed is relevant to your study. If you are not sure if a list item applies to your research, read the appropriate section before selecting a response.

### Materials & experimental systems

| n/a | Involved in the study | |
|---|---|---|
| ☐ | ☒ | Antibodies |
| ☐ | ☒ | Eukaryotic cell lines |
| ☒ | ☐ | Palaeontology and archaeology |
| ☐ | ☒ | Animals and other organisms |
| ☒ | ☐ | Clinical data |
| ☒ | ☐ | Dual use research of concern |

### Methods

| n/a | Involved in the study | |
|---|---|---|
| ☒ | ☐ | ChIP-seq |
| ☐ | ☒ | Flow cytometry |
| ☒ | ☐ | MRI-based neuroimaging |

## Antibodies

| | |
|---|---|
| Antibodies used | Rat anti-IgG2b isotype (BioXCell, Cat#BE0090; Clone#LTF-2; RRID:AB_1107780); Mouse anti-CD8α (BioXCell, Cat#BE0061; Clone#2.43; RRID:AB_1125541); Mouse anti-PD-1(CD279) (BioXCell, Cat#BE0146; Clone# RMP1-14; RRID:AB_10949053); Mouse PerCP/Cyanine5.5 anti-Ki-67 Antibody (Biolegend, Cat#652423; Clone#16A8; RRID:AB_2629530); Brilliant Violet 605™ anti-T-bet Antibody |

(Biolegend, Cat#644817; Clone#4B10; RRID:AB_11219388); Mouse APC anti-CD223 (LAG-3) Antibody (Biolegend, Cat#125209; Clone#C9B7W; RRID:AB_10639935); Mouse PerCP/Cyanine5.5 anti-CD366 (Tim-3) Antibody (Biolegend, Cat#134012; Clone#B8.2C12; RRID:AB_2632736); PE anti-TCF1 (TCF7) Antibody (Biolegend, Cat#655207; Clone#7F11A10; RRID:AB_2728491); Human/mouse/rat FITC anti-CD278 (ICOS) Antibody (Biolegend, Cat#313505; Clone#C398.4A; RRID:AB_416329); Human/mouse FITC anti-Granzyme B Recombinant Antibody (Biolegend, Cat#372205; Clone#QA16A02; RRID:AB_2687029); Mouse PE/Cyanine5 anti-CD69 Antibody (Biolegend, Cat#104509; Clone#H1.2F3; RRID:AB_313112); FITC anti-mouse CD63 Antibody (Biolegend, Cat#143919; Clone#NVG-2; RRID:AB_2876488); Mouse APC anti-CD152 Antibody (Biolegend, Cat#106309; Clone#UC10-4B9; RRID:AB_2230158); Mouse APC anti-CD279 (PD-1) Antibody (Biolegend, Cat#135209; Clone#29F.1A12; RRID:AB_2251944); Mouse PE/Cyanine5 anti-CD4 Antibody (Biolegend, Cat#100409; Clone#GK1.5; RRID:AB_312694); Mouse Brilliant Violet 421™ anti-IL-2 Antibody (Biolegend, Cat#503825; Clone#JES6-5H4; RRID:AB_10895901); Mouse APC anti-CD45.2 Antibody (Biolegend, Cat#109813; Clone#104; RRID:AB_389210); Mouse APC anti-IFN-γ Antibody (Biolegend, Cat#505810; Clone#XMG1.2; RRID:AB_315404); Human/mouse PE/Cyanine7 anti-Granzyme B Recombinant Antibody (Biolegend, Cat#372213; Clone#QA16A02; RRID:AB_2728380); Mouse PerCP/Cyanine5.5 anti-TNF-α Antibody (Biolegend, Cat#506321; Clone#MP6-XT22; RRID:AB_961435); Mouse Brilliant Violet 711™ anti-CD8a Antibody (Biolegend, Cat#100747; Clone#53-6.7; RRID:AB_11219594); Mouse Brilliant Violet 421™ anti-FOXP3 Antibody (Biolegend, Cat#126419; Clone#MF-14; RRID:AB_2565933); Mouse APC anti-CD3 Antibody (Biolegend, Cat#100235; Clone#17A2; RRID:AB_2561455); FITC anti-Bcl-2 (Biolegend, Cat#633503; Clone#BCL/10C4; RRID:AB_2028392); Mouse APC anti-CD98 (4F2) (Biolegend, Cat#128211; Clone#RL388; RRID:AB_2750544); Mouse FITC anti-F4/80 Recombinant Antibody (Biolegend, Cat#157309; Clone#QA17A29; RRID:AB_2876535); Mouse APC anti-Ly-6G (Gr1) Antibody (Biolegend, Cat#127613; Clone#1A8; RRID:AB_1877163); Mouse/human APC anti-CD11b Antibody (Biolegend, Cat#101211; Clone#M1/70; RRID: AB_312794); Mouse PerCP anti-CD11c Antibody (Biolegend, Cat#117325; Clone#N418; RRID: AB_893236); Alexa Fluor® 647 anti-mouse CD16 Antibody (Biolegend, Cat#158021; Clone#S17014E; RRID: AB_2904300); PE/Cyanine5 anti-mouse CD28 Antibody (Biolegend, Cat#102108; Clone#37.51; RRID: AB_312873); Mouse PE/Cyanine7 anti-CD14 Antibody (Biolegend, Cat#123315; Clone#Sa14-2; RRID:AB_10641133); Mouse/human PE anti-Ki-67 Antibody (Biolegend, Cat#151210; Clone#11F6; RRID:AB_2716008); PE anti-Lck Phospho (Tyr394) (Biolegend, Cat#933103; Clone#A18002D; RRID:AB_2820203); PE TOX Monoclonal Antibody (TXRX10) (Thermo Fisher Scientific, Cat#12-6502-82; Clone#TXRX10; RRID:AB_10855034); APC Phospho-CREB (Ser133) Recombinant Rabbit Monoclonal Antibody (Thermo Fisher Scientific, Cat#MA5-36992; Clone#CREBS133-4D11; RRID:AB_2896927); Rabbit PE Active Caspase-3 (Thermo Fisher Scientific, Cat#BDB561011; Clone#C92-605; RRID:AB_2033931); Rabbit PE Phospho-Stat1 (Tyr701) Recombinant Monoclonal Antibody (Thermo Fisher Scientific, Cat#MA5-37039; Clone#Stat1Y701-3E6; RRID:AB_2896974); GPR43 Polyclonal Antibody (Thermo Fisher Scientific, Cat#PA5-111780; Clone#N/A; RRID:AB_2857189); Biotin Monoclonal Antibody (Z021) (Thermo Fisher Scientific, Cat#03-3700; Clone#Z021; RRID:AB_2532265); PKA C-α Antibody (Cell Signaling Technology, Cat#4782S; Clone#N/A; RRID:AB_2170170); Rabbit Stat1 (D1K9Y) mAb (Cell Signaling Technology, Cat#14994S; Clone#D1K9Y; RRID:AB_2737027); Mouse monoclonal anti-β-actin antibody (Sigma-Aldrich, Cat#A1978; Clone#AC-15; RRID:AB_476692); Goat anti-Mouse IgG (H+L) Secondary Antibody,HRP (Thermo Fisher Scientific, Cat#31430; Clone#N/A; RRID:AB_228307); Goat anti-Rabbit IgG (H+L) Secondary Antibody, HRP (Thermo Fisher Scientific, Cat#31460; Clone#N/A; RRID:AB_228341); Goat Polyclonal IFN-alpha/beta R1 Antibody (Novus, Cat#AF3039-SP; Clone#N/A; RRID:AB_664107); Hamster Monoclonal TNF RI/TNFRSF1A Antibody (Novus, Cat#MAB430-SP; Clone#55R170; RRID:AB_2208782).

Validation

The specificities of listed FACS antibodies have been validated by the manufacturer by flow cytometry.
Mouse PerCP/Cyanine5.5 anti-Ki-67 Antibody: https://www.biolegend.com/en-us/antibodies-and-more/percp-cyanine5-5-anti-mouse-ki-67-antibody-13463?GroupID=GROUP26
Brilliant Violet 605™ anti-T-bet Antibody: https://www.biolegend.com/en-ie/products/brilliant-violet-605-anti-t-bet-antibody-7907
Mouse APC anti-CD223 (LAG-3) Antibody: https://www.biolegend.com/en-us/products/apc-anti-mouse-cd223-lag-3-antibody-6926?GroupID=BLG5408
Mouse PerCP/Cyanine5.5 anti-CD366 (Tim-3) Antibody: https://www.biolegend.com/nl-be/products/percp-cyanine5-5-anti-mouse-cd366-tim-3-antibody-13043?GroupID=BLG10787
PE anti-TCF1 (TCF7) Antibody: https://www.biolegend.com/en-us/products/pe-anti-tcf1-tcf7-antibody-15529?GroupID=GROUP26
Human/mouse/rat FITC anti-CD278 (ICOS) Antibody: https://www.biolegend.com/en-ie/products/fitc-anti-human-mouse-rat-cd278-icos-antibody-2481
Human/mouse FITC anti-Granzyme B Recombinant Antibody: https://www.biolegend.com/en-gb/cell-health/fitc-anti-human-mouse-granzyme-b-recombinant-antibody-14430?GroupID=GROUP28
FITC anti-mouse CD63 Antibody: https://www.biolegend.com/en-us/search-results/fitc-anti-mouse-cd63-antibody-18771?GroupID=BLG10807&gclid=Cj0KCQjwpc-oBhCGARIsAH6ote-aQdT8Vkq1cXPCsjfNUUYuf69yZSkqUJB4kXS5eqKXf04vVJqHPzAaAqUKEALw_wcB
Mouse APC anti-CD152 Antibody: https://www.biolegend.com/en-us/explore-new-products/apc-anti-mouse-cd152-antibody-5455?GroupID=BLG10448
Mouse APC anti-CD279 (PD-1) Antibody: https://www.biolegend.com/nl-nl/products/apc-anti-mouse-cd279-pd-1-antibody-6497?GroupID=BLG7928
Mouse PE/Cyanine5 anti-CD4 Antibody: https://www.biolegend.com/en-us/products/pe-cyanine5-anti-mouse-cd4-antibody-251?GroupID=BLG4745
Mouse Brilliant Violet 421™ anti-IL-2 Antibody: https://www.biolegend.com/en-gb/products/brilliant-violet-421-anti-mouse-il-2-antibody-7202?GroupID=GROUP24
Mouse APC anti-CD45.2 Antibody: https://www.biolegend.com/en-ie/productstab/apc-anti-mouse-cd45-2-antibody-2759
Mouse APC anti-IFN-γ Antibody: https://www.biolegend.com/en-us/products/apc-anti-mouse-ifn-gamma-antibody-993?GroupID=GROUP24
Human/mouse PE/Cyanine7 anti-Granzyme B Recombinant Antibody:https://www.biolegend.com/en-us/sean-tuckers-tests/pe-cyanine7-anti-humanmouse-granzyme-b-recombinant-antibody-15582?GroupID=GROUP28
Mouse PerCP/Cyanine5.5 anti-TNF-α Antibody: https://www.biolegend.com/nl-be/products/percp-cyanine5-5-anti-mouse-tnf-alpha-antibody-4438
Mouse Brilliant Violet 711™ anti-CD8a Antibody: https://www.biolegend.com/nl-be/products/brilliant-violet-711-anti-mouse-cd8a-antibody-7926
Mouse Brilliant Violet 421™ anti-FOXP3 Antibody: https://www.biolegend.com/nl-be/products/brilliant-violet-421-anti-mouse-foxp3-antibody-12143
Mouse APC anti-CD3 Antibody: https://www.biolegend.com/nl-be/products/apc-anti-mouse-cd3-antibody-8055
FITC anti-Bcl-2: https://www.biolegend.com/nl-be/products/fitc-anti-bcl-2-antibody-6345
Mouse APC anti-CD98 (4F2): https://www.biolegend.com/nl-be/products/apc-anti-mouse-cd98-4f2-antibody-16555
Mouse FITC anti-F4/80 Recombinant Antibody: https://www.biolegend.com/nl-be/products/fitc-anti-mouse-f480-recombinant-antibody-19715

Mouse APC anti-Ly-6G (Gr1) Antibody: https://www.biolegend.com/nl-be/products/apc-anti-mouse-ly-6g-antibody-6115
Mouse/human APC anti-CD11b Antibody: https://www.biolegend.com/nl-be/products/apc-anti-mouse-human-cd11b-antibody-345
Mouse PerCP anti-CD11c Antibody: https://www.biolegend.com/nl-be/products/percp-anti-mouse-cd11c-antibody-4259
Alexa Fluor® 647 anti-mouse CD16 Antibody: https://www.biolegend.com/nl-be/products/alexa-fluor-647-anti-mouse-cd16-antibody-21516
PE/Cyanine5 anti-mouse CD28 Antibody: https://www.biolegend.com/nl-be/products/pe-cyanine5-anti-mouse-cd28-antibody-116
Mouse PE/Cyanine7 anti-CD14 Antibody: https://www.biolegend.com/en-gb/products/pe-cyanine7-anti-mouse-cd14-antibody-6924
Mouse/human PE anti-Ki-67 Antibody: https://www.biolegend.com/en-gb/products/pe-anti-mouse-human-ki-67-antibody-14888
PE anti-Lck Phospho (Tyr394): https://www.biolegend.com/en-gb/products/pe-anti-lck-phospho-tyr394-antibody-18616
PE TOX Monoclonal Antibody (TXRX10): https://www.thermofisher.com/antibody/product/TOX-Antibody-clone-TXRX10-Monoclonal/12-6502-82
APC Phospho-CREB (Ser133) Recombinant Rabbit Monoclonal Antibody:https://www.thermofisher.com/antibody/product/Phospho-CREB-Ser133-Antibody-clone-CREBS133-4D11-Recombinant-Monoclonal/MA5-36992
Rabbit PE Active Caspase-3: https://www.thermofisher.com/antibody/primary/target/caspase%203
Rabbit PE Phospho-Stat1 (Tyr701) Recombinant Monoclonal Antibody: https://www.thermofisher.com/antibody/product/Phospho-Stat1-Tyr701-Antibody-clone-Stat1Y701-3E6-Recombinant-Monoclonal/MA5-37038
Mouse PE/Cyanine5 anti-CD69 Antibody: https://www.biolegend.com/en-us/products/pe-cyanine5-anti-mouse-cd69-antibody-266

The specificities of listed WB antibodies have been validated by the manufacturer by western blot.
Rat anti-IgG2b isotype: https://bioxcell.com/invivomab-rat-igg2b-isotype-control-anti-keyhole-limpet-hemocyanin-be0090
Mouse anti-CD8α: https://bioxcell.com/invivomab-anti-mouse-cd8a-be0061
Mouse anti-PD-1(CD279): https://bioxcell.com/invivomab-anti-mouse-pd-1-cd279-be0146
GPR43 Polyclonal Antibody: https://www.fishersci.com/shop/products/gpr43-polyclonal-antibody-invitrogen-5/PIPA5111780
Biotin Monoclonal Antibody (Z021): https://www.thermofisher.com/antibody/product/Biotin-Antibody-clone-Z021-Monoclonal/03-3700
Rabbit Stat1 (D1K9Y) mAb: https://www.cellsignal.com/products/primary-antibodies/stat1-d1k9y-rabbit-mab/14994
PKA C-α Antibody: https://www.cellsignal.com/products/primary-antibodies/pka-c-a-antibody/4782?N=4294956287&Ntt=pka&fromPage=plp&_requestid=4398756
Mouse monoclonal anti-β-actin antibody: https://www.sigmaaldrich.com/US/en/product/sigma/a1978
Goat anti-Mouse IgG (H+L) Secondary Antibody,HRP: https://www.thermofisher.com/antibody/product/Goat-anti-Mouse-IgG-H-L-Secondary-Antibody-Polyclonal/31430
Goat anti-Rabbit IgG (H+L) Secondary Antibody, HRP: https://www.thermofisher.com/antibody/product/Goat-anti-Rabbit-IgG-H-L-Secondary-Antibody-Polyclonal/31460
Goat Polyclonal IFN-alpha/beta R1 Antibody: https://www.novusbio.com/products/ifn-alpha-beta-r1-antibody_af3039
Hamster Monoclonal TNF RI/TNFRSF1A Antibody: https://www.novusbio.com/products/tnf-ri-tnfrsf1a-antibody-55r170_mab430

## Eukaryotic cell lines

Policy information about cell lines and Sex and Gender in Research

**Cell line source(s)**: Jurkat T cells (ATCC, Cat#TIB-152; RRID:CVCL_0367), LLC1 cells (ATCC, Cat#CRL-1642; RRID:CVCL_4358), B16F10 cells (ATCC, Cat#CRL-6475; RRID:CVCL_0159), E0771cells (ATCC, Cat#CRL-3461; RRID:CVCL_GR23) were purchased from ATCC. MC38 cells (Kerafast, Cat#ENH204-FP; RRID:CVCL_B288) were purchased from Kerafast. Plat-E cells and B16-Ova cells were kindly provided by Dr. Hongbo Chi. RS4;11 cells were kindly provided by Dr. Wendy Stock. Jurkat T cell line expressing PD-1 was generated in-house.

**Authentication**: The cell lines used were not authenticated.

**Mycoplasma contamination**: Cell lines were not tested for mycoplasma contamination.

**Commonly misidentified lines (See ICLAC register)**: No commonly misidentified cell lines were used.

## Animals and other research organisms

Policy information about studies involving animals; ARRIVE guidelines recommended for reporting animal research, and Sex and Gender in Research

**Laboratory animals**: Mouse were housed and bred at the University of Chicago Animal Resource Center in specific pathogen-free conditions. Mice were on 12-hour light/dark cycles that coincide with daylight in Chicago, IL, USA, housing facility was maintained at 20-25 °C and 30-70% humidity. C57BL/6J (The Jackson Laboratory, JAX:000664; RRID:IMSR_JAX:000664), C57BL/6 nude (B6.Cg-Foxn1nu/J) ((The Jackson Laboratory, JAX:000819; RRID:IMSR_JAX:000819), TCRα Knock-out (B6.129S2-Tcratm1Mom/J) (The Jackson Laboratory, JAX:002116; RRID:IMSR_JAX:002116), Pmel-1 (B6.Cg-Thy1a/CyTg(TcraTcrb)8Rest/J) (The Jackson Laboratory, JAX:005023; RRID:IMSR_JAX:0050), OT-I (C57BL/6-Tg(TcraTcrb)1100Mjb/J) (The Jackson Laboratory, JAX:003831; RRID:IMSR_JAX:003831), Cd8acre (C57BL/6-Tg(Cd8a-cre)1Itan/J) (The Jackson Laboratory, JAX:008766; RRID:IMSR_JAX:008766) mice were purchased from The Jackson Laboratory. Gpr43-/- and Gpr43fl/fl mice were kindly provided by Dr. Brian Layden. Sex-and age-matched mice were used throughout the study at 7-12 weeks old, and both male and female mice were used. The genetically modified mice were viable and developed normally.

**Wild animals**: The study did not involve wild animals.

**Reporting on sex**: Both male and female mice were included in all analyses reported in this manuscript, as there were no differences between sexes observed in any of our experiments.

| | |
|---|---|
| Field-collected samples | The study did not involve samples collected from the field. |
| Ethics oversight | Animal experiments were conducted and designed according to protocols approved by the Institutional Animal Care and Use Committee of The University of Chicago. |

Note that full information on the approval of the study protocol must also be provided in the manuscript.

# Flow Cytometry

## Plots

Confirm that:

☒ The axis labels state the marker and fluorochrome used (e.g. CD4-FITC).

☒ The axis scales are clearly visible. Include numbers along axes only for bottom left plot of group (a 'group' is an analysis of identical markers).

☒ All plots are contour plots with outliers or pseudocolor plots.

☒ A numerical value for number of cells or percentage (with statistics) is provided.

## Methodology

| | |
|---|---|
| Sample preparation | Tumor tissue were dissected from euthanized tumor-bearing mice, minced into small pieces (≤ 2 mm) using a scalpel in a dish, and then transferred to a 14 mL round-bottom tube containing 5 mL tumor digestion medium (500 µL Collagenase/ Hyaluronidase Solution, 750 µL 1 mg/mL DNase I Solution, and 3.75 mL RPMI 1640 Medium). After incubation at 37°C for 25 min on a shaking platform, the digested tumor tissues were transferred into a 70 µm mesh nylon strainer on a 50 mL conical tube, pushed through the strainer using the rubber end of a syringe plunger, and rinsed with the recommended medium. After centrifugation at 300×g for 10 min at room temperature with the brake on low, the resulting cell pellets were added 10 mL of ammonium chloride solution for incubation at room temperature for 5 minutes, followed by centrifugation at 300×g for 10 minutes at room temperature with the brake on low. The resulting cell pellets were re-suspended at 1-10 ×106 cells/ mL in PBS and then subjected to CD45+ tumor-infiltrating leukocytes isolation by magnetic bead purification using EasySep™ Mouse TIL (CD45) Positive Selection Kit according to the manufacturer's instructions (Stemcell Technologies). |
| Instrument | LSRII, LSR Fortessa (BD Biosciences), or Attune NxT |
| Software | Flowjo 10.4 or later (Tree Star) |
| Cell population abundance | The purities of the sorted cells were more than 98%. |
| Gating strategy | For all experiments FSC-A vs. SSC-A gates of the starting cell population were used to identify viable cells. Singlet cells were identified using FSC-A vs. FSC-H gating. Positive populations were determined by the specific antibodies, which were distinct from negative populations. Isotype control was used to distinguish between background and marker-positive events. |

☒ Tick this box to confirm that a figure exemplifying the gating strategy is provided in the Supplementary Information.
