# Supplementary material for: Trans-vaccenic acid reprograms CD8+ T cells and anti-tumour immunity
Source: Nature. 2023 Nov 22;623(7989):1034–43. doi: 10.1038/s41586-023-06749-3 (PMC10686835; doi:10.1038/s41586-023-06749-3)
Supplement: Supplementary file 11 — Diet formula of control, TVA and CVA diets. [file 41586_2023_6749_MOESM11_ESM.docx]

**Supplementary Table 9 |** **Diet formula**

| **Groups** | **CON** | | **TVA** | | **CVA** | |
| --- | --- | --- | --- | --- | --- | --- |
| % | gm | *kcal* | gm | *kcal* | gm | *kcal* |
| **Protein** | 19 | ***20*** | 19 | ***20*** | 19 | ***20*** |
| **Carbohydrate** | 67 | ***70*** | 67 | ***70*** | 67 | ***70*** |
| **Fat** | 4 | ***10*** | 4 | ***10*** | 4 | ***10*** |
| Total |  | *100* |  | *100* |  | *100* |
| kcal/gm | 3.8 |  | 3.8 |  | 3.8 |  |
|  |  |  |  |  |  |  |
| **Ingredient** | **gm** | ***kcal*** | **gm** | ***kcal*** | **gm** | ***kcal*** |
| Casein | 200 | *800* | 200 | *800* | 200 | *800* |
| L-Cystine | 3 | *12* | 3 | *12* | 3 | *12* |
|  |  |  |  |  |  |  |
| Corn Starch | 506.2 | *2025* | 506.2 | *2025* | 506.2 | *2025* |
| Maltodextrin 10 | 125 | *500* | 125 | *500* | 125 | *500* |
| Sucrose | 68.8 | *275* | 68.8 | *275* | 68.8 | *275* |
|  |  |  |  |  |  |  |
| Cellulose | 50 | *0* | 50 | *0* | 50 | *0* |
|  |  |  |  |  |  |  |
| Soybean Oil | 25 | *225* | 25 | *225* | 25 | *225* |
| Lard | 20 | *180* | 20 | *180* | 20 | *180* |
|  |  |  |  |  |  |  |
| Mineral Mix S10026 | 10 | *0* | 10 | *0* | 10 | *0* |
| DiCalcium Phosphate | 13 | *0* | 13 | *0* | 13 | *0* |
| Calcium Carbonate | 5.5 | *0* | 5.5 | *0* | 5.5 | *0* |
| Potassium Citrate, 1 H2O | 16.5 | *0* | 16.5 | *0* | 16.5 | *0* |
|  |  |  |  |  |  |  |
| Vitamin Mix V10001 | 10 | *40* | 10 | *40* | 10 | *40* |
| Choline Bitartrate | 2 | *0* | 2 | *0* | 2 | *0* |
|  |  |  |  |  |  |  |
| Trans Vaccenic Acid | 0 | *0* | 10.66 | *0* | 0 | *0* |
| Cis Vaccenic Acid | 0 | *0* | 0 | *0* | 10.66 | *0* |
|  |  |  |  |  |  |  |
| FD&C Yellow Dye #5 | 0.04 | *0* | 0 | *0* | 0.05 | *0* |
| FD&C Red Dye #40 | 0 | *0* | 0.05 | *0* | 0 | *0* |
| FD&C Blue Dye #1 | 0.01 | *0* | 0 | *0* | 0 | *0* |
|  |  |  |  |  |  |  |
| **Total** | **1055.05** | ***4057*** | **1065.71** | ***4057*** | **1065.71** | ***4057*** |
|  |  |  |  |  |  |  |
| % Compound | **0** |  | **1.00** |  | **1.00** |  |
